# MicroRNAs show a wide diversity of expression profiles in the developing and mature central nervous system

**DOI:** 10.1186/gb-2007-8-8-r173

**Published:** 2007-08-21

**Authors:** Marika Kapsimali, Wigard P Kloosterman, Ewart de Bruijn, Frederic Rosa, Ronald HA Plasterk, Stephen W Wilson

**Affiliations:** 1 Department of Anatomy and Developmental Biology, UCL, Gower Street, London WC1E 6BT, UK; 2 DEPSN, UPR2197, CNRS, avenue de la Terrasse, 91198, Gif-sur-Yvette, France; 3 Génétique Moléculaire du Développement, INSERM U784, Ecole Normale Supérieure, 46, rue d'Ulm, 75230 Paris, France; 4 Hubrecht Laboratory, Centre for Biomedical Genetics, Uppsalalaan 8, 3584 CT Utrecht, the Netherlands; 5 Génétique Moléculaire du Développement, INSERM U784, Ecole Normale Supérieure, 46, rue d'Ulm, 75230 Paris, France

## Abstract

**Background:**

MicroRNA (miRNA) encoding genes are abundant in vertebrate genomes but very few have been studied in any detail. Bioinformatic tools allow prediction of miRNA targets and this information coupled with knowledge of miRNA expression profiles facilitates formulation of hypotheses of miRNA function. Although the central nervous system (CNS) is a prominent site of miRNA expression, virtually nothing is known about the spatial and temporal expression profiles of miRNAs in the brain. To provide an overview of the breadth of miRNA expression in the CNS, we performed a comprehensive analysis of the neuroanatomical expression profiles of 38 abundant conserved miRNAs in developing and adult zebrafish brain.

**Results:**

Our results show miRNAs have a wide variety of different expression profiles in neural cells, including: expression in neuronal precursors and stem cells (for example, *miR-92b*); expression associated with transition from proliferation to differentiation (for example, *miR-124*); constitutive expression in mature neurons (*miR-124 *again); expression in both proliferative cells and their differentiated progeny (for example, *miR-9*); regionally restricted expression (for example, *miR-222 *in telencephalon); and cell-type specific expression (for example, *miR-218a *in motor neurons).

**Conclusion:**

The data we present facilitate prediction of likely modes of miRNA function in the CNS and many miRNA expression profiles are consistent with the mutual exclusion mode of function in which there is spatial or temporal exclusion of miRNAs and their targets. However, some miRNAs, such as those with cell-type specific expression, are more likely to be co-expressed with their targets. Our data provide an important resource for future functional studies of miRNAs in the CNS.

## Abstract 2

A comprehensive analysis of the neuroanatomical expression profiles of 38 abundant conserved miRNAs in developing and adult zebrafish brain was performed.

## Background

The expression of 30% or more animal genes is regulated by microRNAs (miRNAs) [1,2]. Genes encoding miRNAs are transcribed as polyadenlyated transcripts that are subject to processing mediated by the nuclear RNAseIII Drosha [3] and cytoplasmic RNAseIII Dicer [4], which release a 20-23 nucleotide (nt)-long RNA duplex. One strand of the duplex forms a guide for the RNA induced silencing complex (RISC) to target the 3' untranslated region (UTR) of mRNAs. Binding of mature miRNAs to imperfectly complementary mRNA target sites triggers relocalization of the mRNA to P-bodies [5]. Although the precise mechanism of miRNA-mediated gene silencing remains uncertain, miRNAs can promote de-adenylation that likely destabilizes mRNAs, leads to their clearance [6-8] and/or induces translational repression of target mRNAs [9,10].

Target mRNAs are usually transcribed at low levels whenever their targeting miRNAs are expressed [11,12]. This complementarity can occur through spatial or temporal reciprocity of miRNA and target mRNA gene expression. Thus, miRNAs can promote the clearance of mRNAs remaining from earlier time points or present due to imperfect transcriptional silencing [6,12]. However, other roles for miRNAs are likely, and may involve contemporaneous expression and function of miRNAs and their targets [13,14].

Different experimental approaches indicate hundreds of miRNAs in vertebrate genomes [15-17]. Many miRNAs show spatially and/or temporally restricted expression patterns (for example, [18,19]), including the central nervous system (CNS) (for example, [20-26]). However, spatial and temporal expression profiles and functions of very few vertebrate miRNAs have been examined in detail. Within the vertebrate CNS, proposed roles for miRNAs include neurogenesis [27], regulation of morphogenesis [28], dendrite formation [29], and silencing of non-neural mRNAs [30-32]. miRNAs are also implicated in neurological diseases [33-35]. Although these studies point to the importance of miRNAs in brain development, function and disease, we still have little idea of the range of miRNA activities in neural cells.

Given the known modes of action of miRNAs, knowledge of the temporal and spatial expression profiles of miRNA genes is an important initial step in elucidating their functions. Based on our previous miRNA expression analyses [18,24], we selected 38 conserved vertebrate miRNAs from different families and with distinct expression profiles in the CNS and studied their expression in zebrafish neural tissue from development into adulthood. This analysis reveals a wide diversity in miRNA expression, ranging from single cell types to the majority of CNS cells and from transient to constitutive expression. We describe several classes of expression profile and discuss these in terms of known and predicted modes of action of miRNAs. Our study provides a broad overview of miRNA expression in the brain and a foundation for future functional analyses.

## Results

In order to survey the expression patterns of miRNAs in the brain, we performed *in situ *hybridizations with locked nucleic acid (LNA) probes to 38 different miRNAs (Table J in Additional data file 28,) at 3 and/or 5 days and/or 6 weeks (young adult ('Y-Ad' in Additional data files 1-29)) and/or adult zebrafish (adult ('A' in Additional data files 1-29)). Some of the miRNAs we analyzed belong to the same family or cluster and can differ in only one nucleotide located in, or outside, the 'seed' sequence (Table K in Additional data file 28). To examine if the LNA probes can discriminate between miRNAs having only one or more different nucleotides, we performed *in situ *hybridization for four miRNAs using one or two internal mismatches (Table L in Additional data file 28, and Additional data file 29). We observe that in the case of *let-7a*, *miR-92b *and *miR-153a*, one mismatch strongly reduces the hybridization signal. Since there is still some staining left, we cannot exclude some cross-hybridization with other members of the respective miRNA families. In contrast, two mismatches in the *miR-181a *probe are sufficient to eliminate specific *in situ *hybridization signal, supporting the conclusion that probes with two or more different internal nucleotides detect signal from a single miRNA and not others with similar sequence [36].

We describe the range of different spatially and/or temporally restricted profiles of miRNA expression with illustrative examples below and more comprehensive neuroanatomical documentation in the figures, text and tables in Additional data files 1-27.

### miRNA expression can be restricted to proliferating cells in the larval zebrafish brain

With very few exceptions, the fish CNS is organized such that proliferative cells line the ventricles whereas differentiated neurons migrate away from this zone towards the basal, or pial surface of the brain. At larval (3 and 5 day post-fertilization (dpf)) stages, proliferative cells are present throughout the brain, including the periventricular telencephalic, thalamic and hypothalamic zones, tectal proliferative zone, cerebellar valvula and rhombic lip [37].

**Figure 1:**
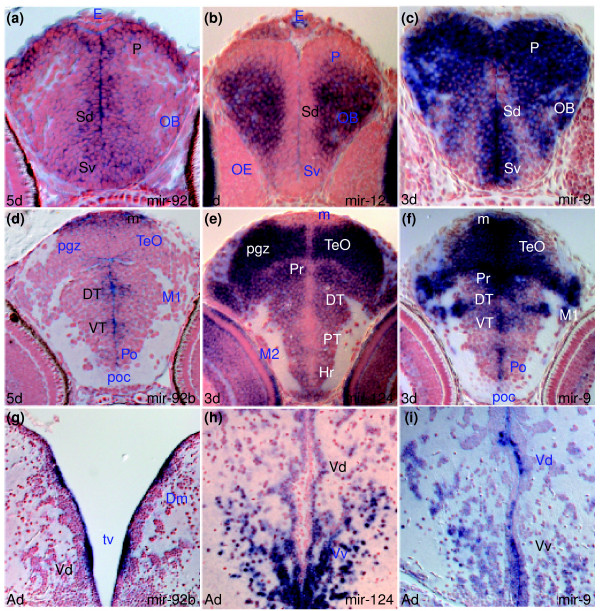
miRNAs expressed in proliferating and/or differentiating cells in the developing and adult zebrafish brain. In this and other figures, unless otherwise mentioned, sections are transverse with dorsal on the top, stage is shown bottom left and miRNA analyzed by *in situ *hybridization bottom right, *in situ *staining is in blue and cell nuclei are visualized with nuclear red counterstaining. Abbreviations used in the Results section of the text are denoted in black. For other abbreviations, see Additional data file 26. **(a,d,g) ***miR-92b *expression in periventricular and adjacent cells of the telencephalon (a,g), diencephalon and optic tectum (d). **(b,e,h) ***miR-124 *expression in differentiating cells in the telencephalon (b,h), diencephalon and optic tectum (e). **(c,f,i) ***miR-9 *expression in periventricular/proliferating and differentiating cells of the telencephalon (c,i), diencephalon and optic tectum (f).

**Figure 2:**
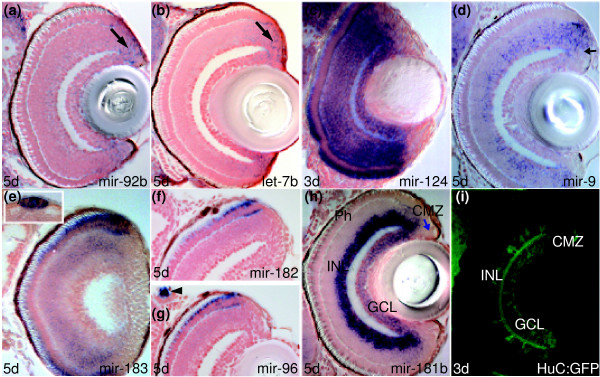
Several miRNAs expressed in discrete retinal cell populations. **(a-h) **Transverse sections through retinae *in situ *hybridized with *miR-92b*, *let-7b*, *miR-124*, *miR-9*, *miR-183*, *miR-182*, *miR-96 and miR-181b *probes. Arrows point at proliferative ciliary marginal zone (CMZ) cells in (a,b,d,h). The inset in (e) shows pineal cells. The arrowhead in (g) indicates *miR-96 *expression in peripheral sensory neuromasts. **(i) **Confocal section through the retina of a transgenic line Tg(huC:GFP) immunostained for GFP. Other miRNAs with expression in the retina include *miR-454a *(Figure C in Additional data file 25), *miR-132 *(Figure E in Additional data file 25), *miR-125b *(Figure F in Additional data file 25) and *miR-181a *(Figure G in Additional data file 13).

*miR-92b *is expressed in proliferative zones throughout the 5 dpf embryonic zebrafish brain. Transcripts are detected in periventricular and adjacent cells of the ventral and dorsal subpallium (Sv and Sd, respectively, in Figure 1a) and pallium (P in Figure 1a), thalamus (dorsal (DT) and ventral (VT) in Figure 1d), hypothalamus, pretectum, tegmentum and hindbrain as well as in the tectal proliferative zone (m in Figure 1d), rhombic lip and retinal ciliary marginal zone (CMZ (with arrow) in Figure 2a; Additional data file 6, and Table A in Additional data file 27). This pattern indicates expression is present in most proliferative neural cells, irrespective of the fates of the progeny of these cells.

Like *miR-92b*, *let-7b *expression is restricted to the CMZ of the retina, with expression absent from all mature retinal neurons (arrow in Figure 2b); *let-7b *has broader expression elsewhere in the larval brain (Additional data file 23). *let-7a *and *let-7c*, which differ in their sequence from *let-7b *by two and one nucleotide, respectively, located outside the seed region, appear to lack this retinal expression, although we cannot exclude that these LNA probes cross-hybridize to various *let-7 *family members in different brain areas (Additional data files 22-24 and 27-29).

### miRNAs can be widely expressed in differentiating CNS cells

In contrast to the restricted expression of *miR-92b *in proliferating neural cells, *miR-124*, *miR-138 *and other miRNAs are expressed in differentiating cells of the larval brain. Among these, *miR-124 *is expressed in virtually all differentiating cells throughout the larval zebrafish brain and retina (Figures 1b,e and 2c; Additional data file 7, and Table B in Additional data file 27) whereas *miR-138 *(Additional data file 11, and Table B in Additional data file 27) shows a widespread but more restricted pattern of expression. Such patterns indicate expression is associated with differentiation with little specificity regarding the identity of the differentiating neural cells.

In mammalian neurons, it is proposed that *miR-124 *targets non-neural transcripts [32,38] and induces neurogenesis [27] and our observation of widespread expression in most CNS neurons would be consistent with this. However, targeting only non-neural transcripts does not fit with the tight association of onset of *miR-124 *expression with the transition from neural progenitor to differentiated neuron. The full range of *miR-124 *targets is unknown and one might predict that in addition to non-neural transcripts, targets may also include genes associated with the neural progenitor state. In support of this, predicted targets [39,40] for zebrafish *miR-124 *include many 'early' neural genes, such as *zic2a*, *pou5f1*, *otx2 *and *slit2 *(see [41] for expression).

### miRNAs can be widely expressed in both proliferating and differentiating CNS cells

In addition to miRNAs with expression restricted to either proliferating or differentiating cells, *miR-9*, *miR-135c*, *miR-153a*, *miR-219 *and members of the *let-7 *family (*let-7a*, *let-7b *and *let-7c*) show expression in both proliferating and differentiating cells of the larval brain (Figures 1c,f; Additional data files 2, 3, 9, 12, 18, and 22-24, and Tables A, B and C in Additional data file 27). For example, *miR-9 *is expressed in telencephalic, diencephalic and tectal periventricular proliferative zones as well as the mature neurons that arise from these domains (Figure 1c,f; Additional data files 2 and 3, and Table A in Additional data file 27). Expression is not ubiquitous in neural cells as some areas such as the epithalamus and hypothalamic lateral torus are devoid of expression (Additional data files 2 and 3, and Table A in Additional data file 27). Additionally, within the retina *miR-9 *is expressed in maturing cells of the CMZ (which are likely to still be proliferative) but expression is maintained only in amacrine cells of the inner nuclear layer (INL in Figure 2d). These patterns indicate expression of some miRNAs is not associated with a transition in the maturation state of the expressing cells.

### miRNAs can show spatially localized expression in the larval brain

**Figure 3:**
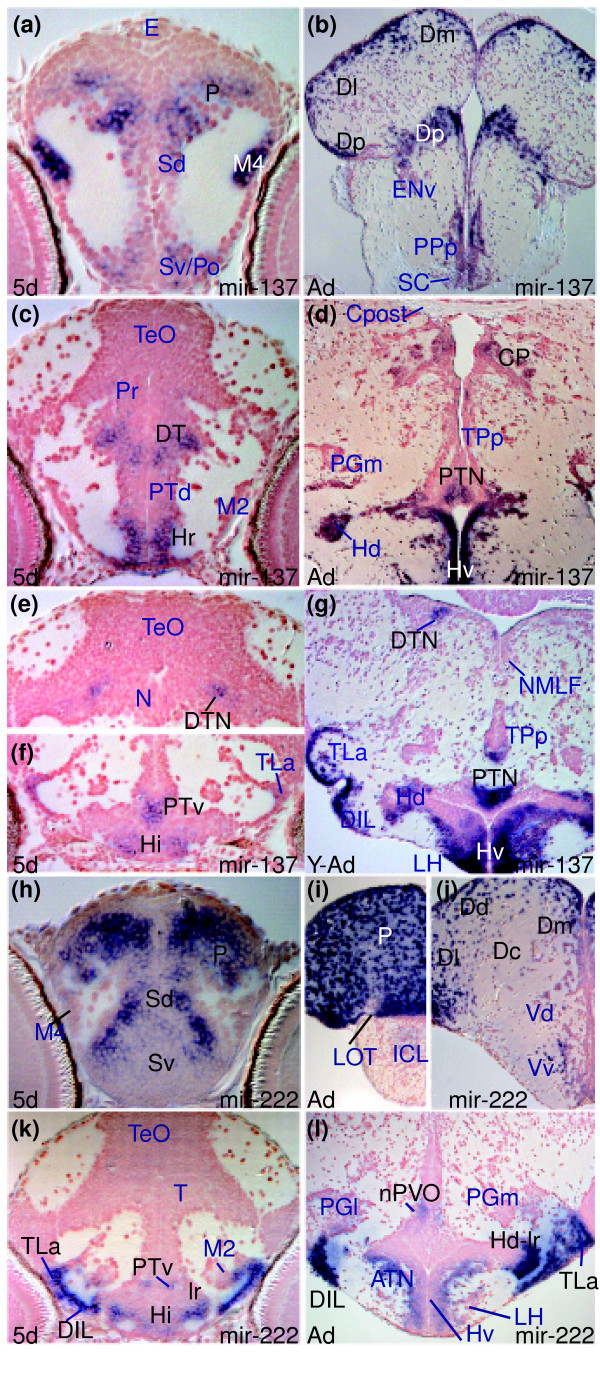
*miR-137 *and *miR-222 *expression is conserved between larval and adult brain. **(a,c,e,f) ***miR-137 *expression in the larval caudal telencephalon (a), diencephalon (c), dorsal midbrain (e) and hypothalamus (f). **(b,d,g) ***miR-137 *expression in adult brain sections at levels corresponding to the embryonic sections shown in (a), (c) and (e/f), respectively. **(h,k) ***miR-222 *expression in the larval telencephalic pallium (P) and subpallium (Sd, Sv), hypothalamus (Hi, TLa, DIL, lr) and posterior tuberculum (PTv, M2). **(i,j,l) ***miR-222 *expression in corresponding adult nuclei in the pallium (P, Dm, Dl, Dd, Dc), subpallium (Vd, Vv), hypothalamus (ATN, LH, TLa, DIL, Hd-lr) and posterior tuberculum (nPVO, PGl).

In contrast to the miRNAs that are broadly expressed in proliferative or differentiated CNS cells, many others, including *miR-128 *and *miR-137*, have larval expression restricted to specific brain areas/nuclei (Additional data files 8 and 10, and Table C in Additional data file 27). For example, at 5 dpf, *miR-137 *expression is restricted to domains of the pallium (P in Figure 3a), dorsal thalamus (DT in Figure 3c), rostral and intermediate hypothalamus (Hr and Hi, respectively, in Figure 3c,f), ventral posterior tubercular area (PTv in Figure 3f) and specific nuclei in the tegmentum (midbrain dorsal tegmental nucleus (DTN in Figure 3e)).

**Figure 4:**
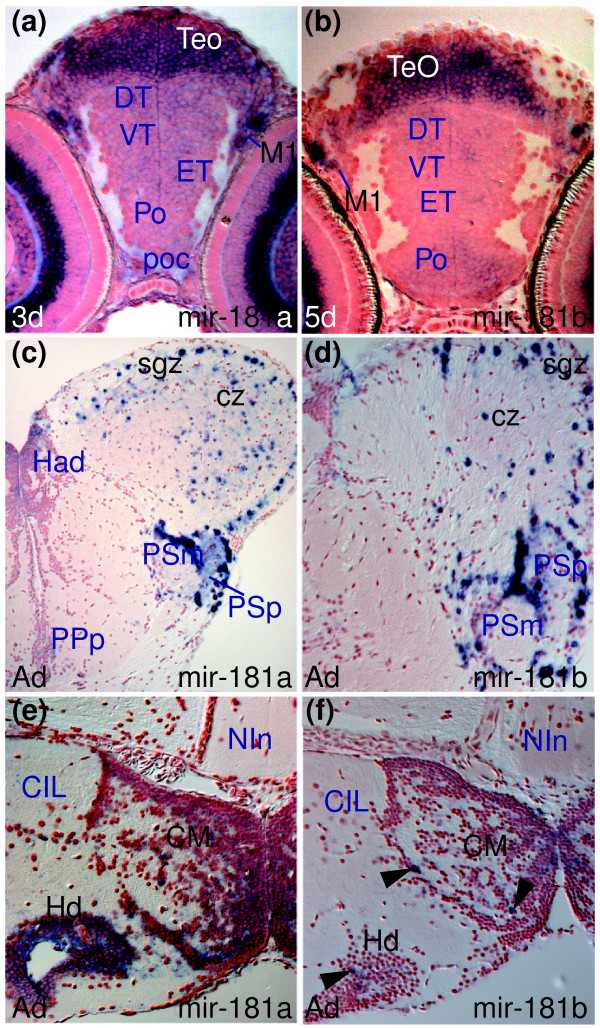
Conserved and divergent expression of *miR-181a *and *miR-181b*. **(a,b) ***miR-181a *and *miR-181b *expression in larval tectal (TeO) and migrated pretectal area cells (M1). **(c,d) **Comparable *miR-181a *and *miR-181b *expression in the adult optic tectum (sgz, cz) and pretectal nuclei (PSm, PSp). **(e) ***miR-181a *is expressed in more cells than **(f) ***miR-181b *(arrowheads) in the adult hypothalamic mamillary body (CM) and dorsal periventricular zone (Hd).

*miR-181a *and *miR-181b *belong to the same family but differ in three nucleotides outside the seed region, suggesting that LNA probes can discriminate between their transcript expression profiles (Tables K and L in Additional data file 28, and Additional data file 29). We observe that both are expressed in cells associated with the visual system, including retinal amacrine cells (INL) and ganglion cells (GCL in Figure 2h; Additional data files 13 and 14). This pattern is highly reminiscent of expression of the *huC *gene (Figure 2i), which encodes an RNA binding protein expressed in nearly all CNS neurons but the same subsets of retinal cells as *miR-181a *and *miR-181b*. Both miRNAs are also expressed in migrated pretectal (M1 in Figure 4a,b) and tectal cells (TeO in Figure 4a,b) and more weakly in many differentiating cells throughout the brain, with some stronger sites of expression in central pallium and medulla oblongata (Additional data files 13 and 14, and Table C in Additional data file 27).

**Figure 5:**
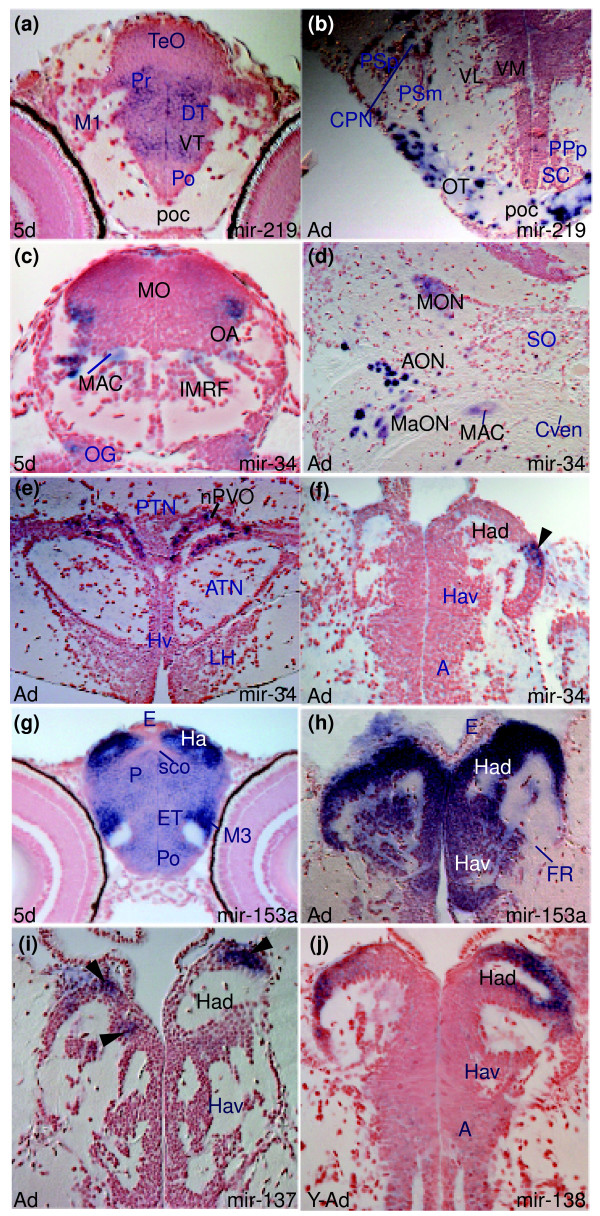
Examples of miRNAs showing differences in expression between larval and adult stages. **(a,b) ***miR-219 *expression in the diencephalon at the level of the post-optic commissure (poc) of the larval and adult brain. In the larval brain (a), *miR-219 *is widely expressed in the ventral (VT) and dorsal (DT) thalamus and periventricular pretectum (Pr) whereas cells of the poc are devoid of expression. In contrast, in the adult (b), *miR-219 *is expressed in cells in the poc and optic tract (OT) whereas ventrolateral (VL) and ventromedial (VM) thalamic nuclei are devoid of expression. **(c-f) ***miR-34 *expression: (c,d) show conserved *miR-34 *expression in the octaval area (OA, MON, AON, MaON) and Mauthner neuron (MAC) in the larval and adult zebrafish brain, respectively; (e) shows *miR-34 *in the adult nucleus of the paraventricular organ (nPVO) and the arrow in (f) points to *miR-34 *expressing cells in the lateral part of the adult left habenula. **(g,h) **Conserved *miR-153a *expression throughout the larval (Ha) and adult (Hav, Had) habenulae. **(i) ***miR-137 *expression in groups of dorsal habenular cells (Had, arrowheads). **(j) ***miR-138 *expression in groups of dorsal habenular cells (Had).

*miR-222 *and *miR-34 *are expressed in neural cells in restricted subdivisions along the rostro-caudal axis of the larval brain. *miR-222 *expression is restricted to specific groups of differentiating cells of the forebrain and midbrain (see also [19]), including telencephalon (P, Sd, and Sv, Figure 3h) eminentia thalami (ET in Figure C of Additional data file 20) and hypothalamic areas (Hi, lateral recess area (lr), diffuse nucleus of inferior lobe (DIL) and lateral torus (TLa) in Figure 3k; Additional data file 20, and Table D in Additional data file 27). In contrast, *miR-34 *expression is absent from forebrain and midbrain and present only in the caudal ventral and lateral isthmus and hindbrain, including ventral and lateral medulla oblongata cells (MO in Figure 5c), presumptive octaval area (OA in Figure 5c), reticular formation cells (intermediate reticular formation-(IMRF) and Mauthner cell (MAC) in Figure 5c; Additional data file 5, and Table D in Additional data file 27).

Together these results show that miRNA expression is frequently region or nucleus specific and can vary in the levels of expression from one group of cells to another.

### *miRNA *expression can be cell type specific

**Figure 6:**
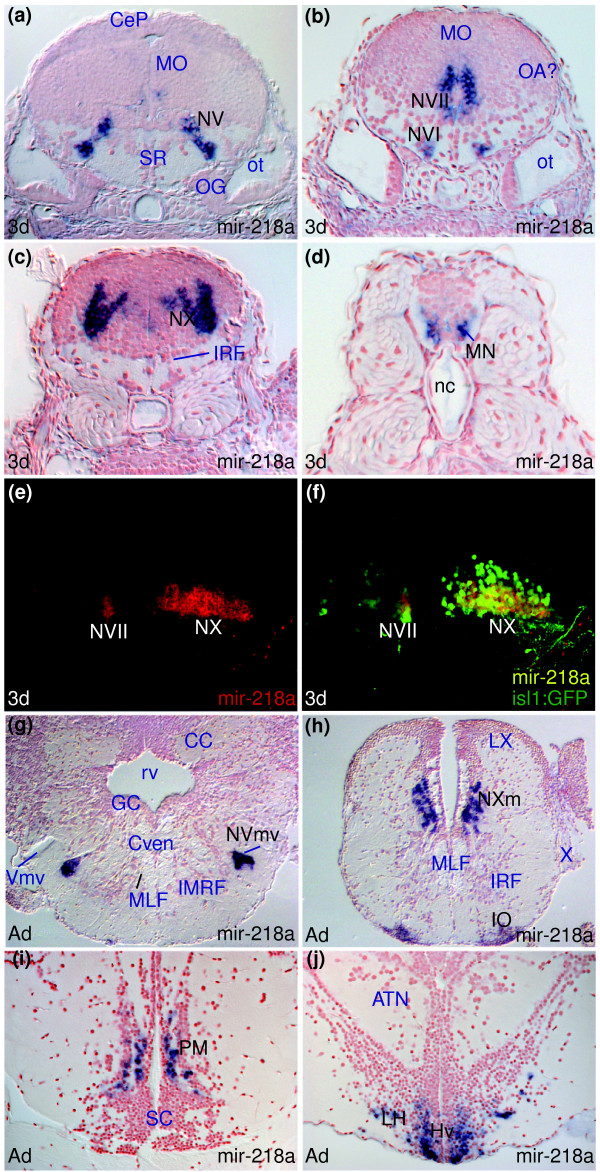
*miR-218a *is expressed in embryonic cranial and spinal motor-neurons. **(a-d,g-h) **Larval and adult *miR-218a *expression in the motor nuclei of the fifth (NV, NVmv), sixth (NVI), seventh (NVII), tenth (NX, NXm) cranial nerves and spinal motor neurons (MN). **(e,f) **Confocal sagittal sections through the hindbrain of an embryo expressing the Tg(isl1:GFP) transgene with anterior to the left. *miR-218a *expression is shown in red in (e) and (f) is a superimposition of the *miR-218a *staining (red) and anti-GFP immunostaining (green). Yellow cells express both *miR-218a *and GFP in the NVII and NX cranial motor nuclei. **(h-j) **Additional sites of expression of *miR-218a *in the adult inferior olive (IO), preoptic magnocellular area (PM) and hypothalamus (Hv, LH).

Several of the miRNAs we examined are expressed in specific cell types at larval stages. For instance, *miR-218a *is exclusively expressed in cranial motor nuclei (NIII, NV, NVI, NVII, NX) and spinal motor neurons (MN, Figure 6a-f; Additional data file 17, and Table E in Additional data file 27).

*miR-183 *is expressed in retinal photoreceptors and weakly in some inner nuclear layer cells, pineal cells that are again likely to be photoreceptors (Figure 2e) and perhaps also in parapineal photoreceptors (Figure C in Additional data file 15). Outside of the CNS, *miR-183 *is expressed in cells that include peripheral sensory neuromasts, olfactory sensory neurons and hair cells of the ear (Additional data file 15, and Table E in Additional data file 27). *miR-182 *and *miR-96 *show almost identical expression patterns to *miR-183 *(Figure 2f,g; and Table E in Additional data file 27), although expression is not as robust. Thus, predominant sites of *miR-183*, *miR-182 *and *miR-96 *expression are sensory cells with modified apical structures. Most/all of these cell types depend upon intraflagellar transport proteins for their development and function [42] and one possibility is that these miRNAs function in intraflagellar transport or cilia function. Many of the genes in these pathways are implicated in human diseases [43]. The highly conserved expression of the three miRNAs is likely due to all being located within about 1 kb of each other on chromosome 4 and, hence, all subject to the same transcriptional regulatory elements [44].

All three miRNAs are also expressed in neurons of the cranial ganglia (Figures D-F in Additional data file 15; and data not shown). The expression of these miRNAs in peripheral sensory neural cells overlaps with *miR-200a *(Additional data file 16), although *miR-200a *lacks the CNS and cranial ganglia expression sites common to *miR-183*, *miR-182 *and *miR-96 *(Table E in Additional data file 27).

Finally, within the brain, *miR-375 *is exclusively expressed in the pituitary and a few scattered weakly labeled hypothalamic cells (Additional data file 21, and Table E in Additional data file 27). Although expression of mouse *miR-375 *in the pituitary has not been ascertained, it is expressed in pancreatic beta cells [45] and appears to function in the regulation of insulin secretion. Given the functional similarities between secretory pituitary cells and pancreatic cells, one may speculate that *miR-375 *has a similar function in both tissues.

In conclusion, in the larval brain, miRNAs show widely divergent profiles of expression, varying from wide to very restricted expression either in particular brain subdivisions/areas or cell types.

### miRNA expression can be largely conserved between larval and adult stages

miRNAs preferentially target mRNAs with spatially or temporally complementary expression [12], raising the possibility that the requirement for such miRNAs may be limited to times when there are spatial or temporal transitions in gene expression. We therefore examined the temporal regulation of miRNA expression between larval and adult stages to determine if miRNAs could maintain expression in the same cells or cell types throughout life.

*miR-92b *is expressed in periventricular cells and proliferative zones in the adult brain as it is in the larval brain. For instance, in both larva and adult, periventricular cells in the medial dorsal subpallium express *miR-92b *(compare Sd in Figure 1a with Vd in Figure 1g; Additional data file 6, and Tables A and F in Additional data file 27). These observations are consistent with the fact that proliferation and production of neurons continues into adulthood in the CNS of zebrafish [46,47]. Several other miRNAs show robust expression in restricted populations of adult ventricular or periventricular cells, including *miR-34b *(Figures I and L in Additional data file 25). Similarly showing conserved expression over time, *miR-124 *expression is excluded from periventricular cells and is detected in most differentiated cells throughout the adult brain as in the larval brain (compare Vd in Figure 1h and Sd in Figure 1b; Additional data file 7, and Tables B and G in Additional data file 27). Such expression in neurons of the adult brain is shared with mouse *miR-124 *[30]. Likewise, *miR-9 *is expressed widely in periventricular zones and in many differentiated cells throughout the adult brain as in the larval brain (for instance, compare Vv in Figure 1i with Sv in Figure 1c; Additional data files 2-4, and Tables A and F in Additional data file 27).

miRNAs with spatially localized expression can also maintain their expression profile into adulthood. For instance, *miR-137 *shows conserved expression in larval and adult brain in cells of the pallium (P in Figure 3a, and Dm, Dl, Dp in Figure 3b), dorsal thalamus (DT in Figure 3c and CP in Figure 3d), posterior tubercular area (PTv in Figure 3f and PTN in Figure 3d,g) and other areas (Additional data file 10). *miR-137 *is expressed in the adult midbrain DTN (Figure 3g) and given the good correspondence of localized tegmental expression between 5 dpf and adult brains, we suggest that the cells in the larval tegmentum correspond to the presumptive midbrain DTN (Figure 3e,g; for other nuclei, see Additional data file 10, and Tables C and H in Additional data file 27). As illustrated by this case, conservation of spatially localized miRNA expression throughout life is helpful for annotation of brain structures at larval stages when neuroanatomical designations have yet to be assigned.

Like *miR-137*, *miR-222 *shows conserved restricted expression in the rostral brain throughout life with domains in the telencephalon (for instance, compare P in Figure 3h with P, Dm, Dd, Dl, and Dc in Figure 3i,j), hypothalamus (DIL, TLa, lr/Hd-lr in Figure 3k,l) and posterior tubercular area (PTv in Figure 3k and nPVO in Figure 3l; see also Additional data file 20, and Tables D and I in Additional data file 27).

These results show that subsequent to their initial induction, some miRNAs conserve their expression in similar proliferating, differentiated or both cell groups throughout life. Although we cannot formally prove that expression is in the same cells over time, our results almost certainly mean that many miRNAs that are induced upon neuronal differentiation maintain constitutive expression throughout the life of the neurons.

### miRNAs of the same family or cluster can show subtle differences in expression in the adult brain

We compared the adult brain expression of miRNAs belonging to the same family, such as *miR-181a *and *miR-181b*, or cluster, such as *miR-221 *and *miR-222*, that differ in three and four nucleotides, respectively; LNA probes should, therefore, discriminate each of them.

*miR-181a *and *miR-181b *show similar expression in the larval brain, and this is largely conserved to adult stages (although there is down-regulation in some areas such as thalamus and tegmentum; Additional data files 13 and 14, and Tables C and H in Additional data file 27). For example, they are expressed in tectal cells (TeO in Figure 4a,b; superficial gray zone (sgz) and central zone (cz) in Figure 4c,d) in both larval and adult brains. Despite overall conservation, we noticed differences in expression of *miR-181a *and *miR-181b *that were not obvious at larval stages. For instance, although both are expressed in the caudal hypothalamus, expression appears to be in different cells (mammilary body (CM) and dorsal periventricular hypothalamus (Hd) in Figure 4e,f; Additional data files 13 and 14). This difference in expression may again be due to genomic duplication of the miRNAs. A cluster on chromosome 8 contains both *miR-181a *and *miR-181b *but there is an additional copy of *miR-181a *on chromosome 22 and of *miR-181b *on chromosome 20 [19,44].

In a similar manner, other miRNAs belonging to a particular cluster seem to largely share expression patterns but also have subtle differences in transcript localization. For instance, *miR-222 *and *miR-221 *share largely similar expression in the adult hypothalamus (ATN, LH, Hd in Figure G of Additional data file 20 and Figure O of Additional data file 19) but only *miR-222 *is expressed in the ventral intermediate hypothalamus at the larval stage (Figure D of Additional data file 20 and Figure B of Additional data file 19; see also Table K in Additional data file 28 for other miRNAs belonging to a single cluster and Additional data file 27 tables for their expression). It is not obvious why there should be differences in *miR-222 *and *miR-221 *expression as they are present in the same cluster and one would predict that they are co-transcribed. There is a precedent for post-transcriptional regulation of miRNA expression [48,49], although this has not been demonstrated for different miRNAs from the same transcript.

### miRNA expression can change between larval and adult stages

Patterns of miRNA expression can change dramatically between larval and adult brain. For instance, in contrast to strong and widespread expression in some domains of the larval brain, adult expression of *miR-219 *is restricted to relatively few cells. For example, larval ventral thalamus (VT in Figure 5a) expresses *miR-219 *but the corresponding adult thalamic nuclei do not (ventrolateral (VL) and ventromedial (VM) in Figure 5b). Conversely, in adults, *miR-219 *is expressed in cells, possibly glia, associated with major tracts such as the lateral olfactory tract, post-optic commissure/optic chiasma and tract (poc and OT, respectively, in Figure 5b) whereas the equivalent pathways in larvae are devoid of staining (Figure 5a). These differences between larva and adult brain suggest downregulation of expression in most cells in the adult brain and either conserved or *de novo *expression in a few discrete cell populations (Additional data file 18, Tables B and G in Additional data file 27). Loss of expression sites is consistent with roles for miRNAs in the regulation of genes that are only transcribed during restricted developmental phases.

Several miRNAs show *de novo *expression in adults that may reflect expression in late differentiating cell types not present or not fully differentiated in larval stages. For instance, *miR-34 *shows conserved expression in the larval and adult hindbrain (Mauthner neuron, (MAC) and presumptive octavolateral area (OA) in Figure 5c; MAC and octavolateral nuclei (medial octavolateral (MON), anterior octaval (AON), magnocellular octaval (MaON) in Figure 5d) but also expands to include forebrain and midbrain cells of the habenulae (Had in Figure 5f), posterior tuberculum (nucleus of paraventricular organ (nPVO) in Figure 5e), pretectum (magnocellular superficial (PSm), accessory (APN) in Figures M and N in Additional data file 5), optic tectum as well as novel areas of the dorsal hindbrain (cerebellar granular layer, facial and vagal lobes; Additional data file 5, and Tables D and I in Additional data file 27). As described above, *miR-222 *expression is also largely conserved between larvae and adults (Figure 3h-l) but *de novo *expression in the adult facial and vagal lobes is also observed (LVII, LX, Figure I in Additional data file 20). Similar to *miR-34*, *miR-218a *expression expands rostrally in the adult brain. In addition to conserved expression in motor nuclei (NVmv, NXm, Figure 6g,h, Additional data file 17), there is adult expression in the ventral telencephalon, preoptic area (magnocellular (PM) in Figure 6i), ventral and lateral hypothalamic nuclei (Hv and LH in Figure 6j), optic tectum and inferior olive (IO in Figure 6h; Additional data file 17, and Table I in Additional data file 27).

In addition to *miR-34 *(Figure 5f), several other miRNAs show spatially restricted expression within the habenulae of adults. *miR-137 *(Figure 5i) and *miR-9 *(Figure G in Additional data file 4) show expression in groups of dorsal lateral habenular cells of the adult brain. Thus, the adult expression of these three miRNAs may correspond to habenular neurons that have not yet formed or fully matured at 5 dpf. This is in contrast to *miR-153a *and *miR-138*, which are expressed in the habenulae in both larvae and adults (Figure 5g,h,j, Figure B in Additional data file 11). *miR-100 *also shows robust habenular expression (Figure K in Additional data file 25) while *miR-92b *and *miR-34b *are expressed in ventricular cells adjacent to the mature habenular nuclei (Figure J in Additional data file 6, and Figure L in Additional data file 25). This analysis of habenular miRNA profiles illustrates that specific brain nuclei can express combinations of different miRNAs, some throughout life and some associated with differentiation. One of best understood roles for any animal miRNAs is in the determination of left/right asymmetric fates for neurons in *Caenorhabditis elegans *[50] and it will be of interest to determine if vertebrate miRNAs are involved in the establishment of the robust asymmetries in neuronal organization present in the habenulae [51,52].

## Discussion

Our survey of miRNA expression has revealed enormous diversity in the range of expression profiles. Although one cannot make specific conclusions regarding function based upon expression pattern alone, expression profiles do allow one to make generalized predictions regarding miRNA roles. This is useful as despite their prevalence, virtually nothing is known regarding the function of most miRNAs that are expressed in the brain.

A common feature of miRNA function is one of mutually exclusive expression of miRNAs and their target mRNAs [11,12]. In such situations, the miRNA is expressed at high levels in comparison to the target mRNA and the expectation is that miRNA function is to maintain low levels of target gene activity. The mutual exclusion of miRNAs and their targets may be in either space or in time. For instance, in zebrafish, *miR-430 *targets a large number of maternally deposited transcripts at the onset of zygotic transcription [6] and many examples of spatially exclusive expression domains of miRNAs and their targets have been documented (for example, [12,53]).

### Many expression profiles are consistent with the mutual exclusion model of miRNA function

Many of the expression profiles we describe are consistent with the miRNAs being expressed at high levels at times or places complementary to their targets. For instance, *miR-9 *is broadly expressed in both proliferative and differentiated cells in many of its expression domains. However, there are sharp transitions between domains of expression and non-expression, with some structures, such as the habenular nuclei, devoid of expression. The absence of temporally restricted expression and presence of spatially restricted expression is consistent with the main targets of this miRNA being restricted to those regions lacking *miR-9 *expression.

Many miRNAs show expression associated with a transition in the differentiation state of the expressing cells. For instance, *miR-92b *is downregulated in most mature neurons whereas, conversely, *miR-124 *is absent from proliferative cells and widely expressed in differentiated neurons. This profile is one of the best conserved miRNA patterns, as a similar restriction to mature neurons is seen for mouse and fly *miR-124 *[12,30]. These patterns are consistent with miRNAs targeting genes expressed at different phases of differentiation.

### Some CNS miRNAs may constitutively survey fluctuating levels of transcriptionally 'silenced' target mRNAs

miRNAs that are induced when cells transition from one state to another are likely to target mRNAs that are expressed during the initial state and not required during the second state. One might predict this role to be required only for the period following the transition during which there is perdurance of mRNAs that were transcribed during the previous state. Major changes in the transcriptome that occur when cells transition between states are thought to be brought about by transcriptional mechanisms independent of miRNA function and so it seems likely that many target mRNAs will not be actively transcribed once a transition has occurred (for example, [6]). However, many miRNAs that are induced upon neuronal differentiation, such as *miR-124*, appear to show constitutive expression throughout the lifetime of the expressing neuron. One possible explanation for this is that such miRNAs continue to regulate some mRNAs that are expressed and required in the mature neurons and we consider this possibility in the next section.

An additional possibility is that some of the miRNAs initially associated with transition to differentiation constantly survey the transcriptome for fluctuations in mRNA levels of genes that should not be actively transcribed in mature neurons. There are well-described mechanisms for repressing and silencing loci (for example, [54]), but the absolute efficacy of such mechanisms is unknown. Certainly, recent studies suggest that there is a huge variability in the level of transcription at equivalent active loci in different cells [55] and perhaps there is comparable variability at repressed loci.

The constitutive expression of miRNAs such as *miR-124*, *miR-181*, *miR-222 *and others in mature neurons is consistent with an initial role in the clearance of mRNAs from the neuronal precursor stage but later they may fulfill a different role in the surveillance of fluctuations in aberrant transcription from notionally 'silenced' loci. As the silencing of loci is generally efficient, one might conclude that for most of the time in most mature neurons, some miRNAs are doing very little indeed, consistent with the idea that miRNAs confer robustness to programs of gene expression [11,12].

We also find that neurons express multiple miRNAs and it is very likely that most miRNAs have many, perhaps as many as a few thousand, mRNA targets [1,2,6,56]. This raises the possibility that the collection of miRNAs expressed by a single neural cell may target a significant proportion of the entire transcriptome of protein coding genes. Of course, each neural cell requires the expression and function of a certain proportion of all possible protein coding genes. mRNAs from genes required for general cellular machinery are protected from miRNA action through possession of short 3' UTRs that are poor miRNA targets and genes required for specific cellular functions of the specific cell type are often miRNA anti-targets - that is, their 3' UTRs lack sequences that would enable binding of co-expressed miRNAs [12]. Other protein coding genes not required by the cell are transcriptionally silenced and it will be intriguing to determine what percentage of these genes is subject to active surveillance by miRNAs. It is possible that miRNA surveillance may represent a global mechanism for suppressing activity of aberrant transcripts of a significant proportion of protein coding genes not required by the specific cell type.

### Some miRNAs are likely to be co-expressed with their targets and may spatially or temporally modulate protein levels within neural cells

The mutual exclusion model of miRNA function may underlie most functions of animal miRNAs, but in some instances, miRNAs are expressed in the same cells at the same time as their target genes. Such a scenario has been termed an 'incoherent feedforward loop' as the induction of both miRNA and target mRNA would seem to be at crossed-purposes [13]. In such situations, the cell would contemporaneously require both the function of the miRNA and its targets. We find one class of miRNA expression profile highly suggestive of functioning in this way.

A few of the miRNAs we examined show expression restricted to very few cells and usually expressing cells share some characteristics of function and/or form. For instance, *miR-218a *is predominantly expressed in most motor neurons. It is hard to reconcile this observation with any conclusion other than the miRNA is targeting genes that normally function within the expressing cells. There is a great deal of regulation of protein activity levels within cells but there are not many examples of miRNAs involved in such regulation. However, *miR-134 *is localized to dendrites and appears to modulate the levels of activity of a kinase that influences dendrite morphogenesis [29].

Given that some mRNAs are localized and translated locally within neurons (for example, [57,58]), an attractive possibility is that cell-type specific brain miRNAs regulate spatially localized translation of proteins. Localized translation from mRNAs is mediated in part by mRNA binding proteins that promote translation [59,60] and a counterpart to this could be that miRNAs clear mRNAs at sites where protein activity should be low. If such roles do exist, then one would predict additional levels of regulation. For instance, there may exist mechanisms to protect target mRNAs in regions where translation is required. mRNAs can be protected from miRNA attack as seen, for instance, in germ cells [7], although we are unaware of examples of such regulation occurring within different cellular compartments. It is intriguing that the translation of beta-actin in neuronal processes is regulated by Vg1RBP, a protein that binds the 3' UTR of mRNAs [60] and may, therefore, shield the mRNA from miRNAs. Second, there may be additional levels of regulation of miRNA expression or localization. For instance, *miR-134 *transcripts are localized to the dendritic compartments in which they function and miRNA activity is spatially/temporally regulated by extracellular signals [29].

## Conclusion

We analyzed the expression of 38 conserved vertebrate miRNAs in zebrafish neural tissue from development to adulthood. This is the first study describing in detail miRNA brain expression and it shows several classes of expression profiles. It reveals a wide diversity in miRNA expression, ranging from single cell types to the majority of CNS cells and from transient to constitutive expression. Our survey of miRNA expression patterns suggests several modes of action within neural cells. The first is to function in neural stem cells/progenitors and the second is to facilitate the clearance of target mRNAs at spatial or temporal transitions. Subsequent to developmental transitions, miRNAs may constitutively survey target mRNAs to counteract stochastic fluctuations in aberrant transcription. Finally, cell-type specific miRNAs may modulate the spatial and/or temporal regulation of target mRNA translation within mature neural cells.

## Materials and methods

### *In situ *hybridization and antibody labeling

*In situ *hybridization on zebrafish larvae was performed as described previously [18,36]. We used the same *in situ *hybridization protocol for adult CNS tissue with some minor modifications. Adult brains were dissected and fixed overnight at 4°C in 4% paraformaldehyde. Proteinase K (10 μg/ml in phosphate buffered saline, 0.1% Tween-20 (PBST)) treatment was done twice for 30 minutes at 37°C with continuous shaking. The acetylation step in the *in situ *hybridization was often omitted since it did not change or improve signal. Post antibody washes were performed for 6 times for 30 minutes each at room temperature then overnight at 4°C and 6 further 15-minute washes at room temperature. Alkaline phosphatase enzymatic reaction was detected using the NBT-BCIP substrate and embryos were subsequently dehydrated to benzyl benzoate/benzyl alcohol. Preparations were subsequently sectioned (see below). miRNA probe sequences and annealing temperatures can be found in Table J in Additional data file 28.

Three days old Tg(huc:gfp) [61] or Tg(isl1:gfp) [62] embryos were fixed and processed for whole mount *in situ *hybridization as described above and/or immuno-cytochemistry using rabbit anti-green fluorescent protein (GFP) antibody (Torrey Pines Biolabs, Houston, Texas, USA) at 1:1,000 and goat anti-rabbit Alexa 488 conjugated (Invitrogen-Molecular Probes, Paris, France) at 1:1,000. When both methods were combined, alkaline phosphatase enzymatic reaction was performed using Fast Red (Roche, Paris, France) as substrate. Light microscopy images were acquired using a Nikon camera attached to a Leica or Nikon upright microscope and analyzed using Photoshop and Illustrator (Adobe) software. Confocal analysis was performed using a Leica TCS SP Confocal microscope using 25×/40× oil immersion objectives and a series of images were acquired at 0.8-2.5 μm intervals. Selected depths were projected by a combination of maximum intensity and opacity.

### Sectioning

Whole larvae and brains stained by whole-mount *in situ *hybridization were transferred from benzyl benzoate/benzyl alcohol to 100% methanol and incubated for 10 minutes. Specimens were washed twice with 100% ethanol for 10 minutes and incubated overnight in 100% Technovit 8100 infiltration solution (Kulzer, Leiden, Netherlands)) at 4°C. Next, specimens were transferred to a mold and embedded overnight in Technovit 8100 embedding medium (Kulzer) deprived of air at 4°C. Sections of 7 μm thickness were cut with a microtome (Reichert-Jung 2050, Leica, Rijswijk, Netherlands)), stretched on water and mounted on glass slides. Sections were dried overnight. Counterstaining was done with 0.05% neutral red for 12 s, followed by extensive washing with water. Sections were preserved with Pertex and mounted under a coverslip. Penetration of all reagents even in adult brains was generally very good, although in some preparations deep tissue in the brain did not label well and readers should be cautious about interpretation regarding absence of expression in such cases.

### Neuroanatomical annotation

Annotation of brain areas was made according to the atlases available for the embryonic and adult zebrafish [63,64] and available anatomical literature on fish brain [65,66]. We have generally used traditional terminology consistent with existing atlases, although various publications have suggested alternative designations. Two notable cases occurred for nomenclature in the diencephalon. It has been suggested that prethalamus and thalamus are better terms for ventral and dorsal thalamus, respectively [67], and tract tracing and transgenic approaches have suggested medial and lateral sub-nuclear sub-divisions of the habenulae [52] that have yet to be reconciled with traditional terminology.

Neuroanatomical documentation of the zebrafish brain, particularly at larval stages, is far from complete and so our annotation is not always definitive. However, for the sake of conciseness and readability, we generally do not use qualifiers such as 'presumptive'. Some of the miRNA expression patterns helped us annotate larval fish brain nuclei. The names of these nuclei are followed by a question mark and the cells belonging to them are delineated by dotted lines wherever possible.

### Predictions of miRNA targets

For predictions of possible mRNA targets of miRNAs, we used prediction programs and related resources available at [39,40].

## Abbreviations

CNS, central nervous system; dpf, days post-fertilization; GFP, green fluorescent protein; LNA locked nucleic acid miRNA, microRNAs; nt, nucleotide; UTR, untranslated region. A full list of neuroanatomical abbreviations is provided at the end of additional data file 26.

## Authors' contributions

MK carried out the neuroanatomical analysis of the data, immunohistochemistry experiments and drafted the manuscript. WK carried out the in situ hybridization and sectioning experiments and participated in the design of the study and drafting of the manuscript. EB participated in the in situ hybridization and sectioning experiments. FR revised critically the manuscript. SW participated in the organization of the data and drafted the manuscript. RP conceived the study, and participated in its design and coordination and helped to draft the manuscript. All authors read and approved the final manuscript.

## Additional data files

The following additional data are available with the online version of this paper. Additional data file 1 is a figure showing *miR-7 *expression in the zebrafish brain. Additional data file 2 is a figure showing *miR-9 *expression in the 3 dpf zebrafish brain. Additional data file 3 is a figure showing *miR-9 *expression in the 5 dpf zebrafish brain. Additional data file 4 is a figure showing *miR-9 *expression in the adult zebrafish brain. Additional data file 5 is a figure showing *miR-34 *expression in the zebrafish brain. Additional data file 6 is a figure showing *miR-92b *expression in the zebrafish brain. Additional data file 7 is a figure showing *miR-124 *expression in the zebrafish brain. Additional data file 8 is a figure showing *miR-128 *expression in the zebrafish brain. Additional data file 9 is a figure showing *miR-135c *expression in the zebrafish brain. Additional data file 10 is a figure showing *miR-137 *expression in the zebrafish brain. Additional data file 11 is a figure showing *miR-138 *expression in the zebrafish brain. Additional data file 12 is a figure showing *miR-153a *expression in the zebrafish brain. Additional data file 13 is a figure showing *miR-181a *expression in the zebrafish brain. Additional data file 14 is a figure showing *miR-181b *expression in the zebrafish brain. Additional data file 15 is a figure showing *miR-183 *expression in the zebrafish brain. Additional data file 16 is a figure showing *miR-200a *expression in the zebrafish brain. Additional data file 17 is a figure showing *miR-218a *expression in the zebrafish brain. Additional data file 18 is a figure showing *miR-219 *expression in the zebrafish brain. Additional data file 19 is a figure showing *miR-221 *expression in the zebrafish brain. Additional data file 20 is a figure showing *miR-222 *expression in the zebrafish brain. Additional data file 21 is a figure showing *miR-375 *expression in the zebrafish brain. Additional data file 22 is a figure showing *let-7a *expression in the zebrafish brain. Additional data file 23 is a figure showing *let-7b *expression in the zebrafish brain. Additional data file 24 is a figure showing *let-7c *expression in the zebrafish brain. Additional data file 25 is a figure showing the expression of other miRNAs in the zebrafish brain. Additional data file 26 includes the legends of the figures presented in Additional data files 1, 2, 3, 4, 5, 6, 7, 8, 9, 10, 11, 12, 13, 14, 15, 16, 17, 18, 19, 20, 21, 22, 23, 24, 25 and 29 and the list of neuroanatomical abbreviations. Additional data file 27 includes Tables A-I, which provide detailed information about the expression of each miRNA in different structures of the zebrafish central and/or peripheral nervous system. Additional data file 28 includes Tables J-L, listing the miRNAs analyzed, the family/cluster they belong to and the mismatch LNA probe sequences used to test specificity of corresponding fully matching LNA probes. Additional data file 29 is a figure showing a mismatch test for *let-7a*, *miR-92b*, *miR-153a *and *miR-181a*.

## Supplementary Material

Additional data file 1 *miR-7 *expression in the zebrafish brain. Click here for file

Additional data file 2 *miR-9 *expression in the 3 days post-fertilization (dpf) zebrafish brain. Click here for file

Additional data file 3 *miR-9 *expression in the 5 days post-fertilization (dpf) zebrafish brain. Click here for file

Additional data file 4 *miR-9 *expression in the adult zebrafish brain. Click here for file

Additional data file 5 *miR-34 *expression in the zebrafish brain. Click here for file

Additional data file 6 *miR-92b *expression in the zebrafish brain. Click here for file

Additional data file 7 *miR-124 *expression in the zebrafish brain. Click here for file

Additional data file 8 *miR-128 *expression in the zebrafish brain. Click here for file

Additional data file 9 *miR-135c *expression in the zebrafish brain. Click here for file

Additional data file 10 *miR-137 *expression in the zebrafish brain. Click here for file

Additional data file 11 *miR-138 *expression in the zebrafish brain. Click here for file

Additional data file 12 *miR-153a *expression in the zebrafish brain. Click here for file

Additional data file 13 *miR-181a *expression in the zebrafish brain. Click here for file

Additional data file 14 *miR-181b *expression in the zebrafish brain. Click here for file

Additional data file 15 *miR-183 *expression in the zebrafish brain. Click here for file

Additional data file 16 *miR-200a *expression in the zebrafish brain. Click here for file

Additional data file 17 *miR-218a *expression in the zebrafish brain. Click here for file

Additional data file 18 *miR-219 *expression in the zebrafish brain. Click here for file

Additional data file 19 *miR-221 *expression in the zebrafish brain. Click here for file

Additional data file 20 *miR-222 *expression in the zebrafish brain. Click here for file

Additional data file 21 *miR-375 *expression in the zebrafish brain. Click here for file

Additional data file 22 *let-7a *expression in the zebrafish brain. Click here for file

Additional data file 23 *let-7b *expression in the zebrafish brain. Click here for file

Additional data file 24 *let-7c *expression in the zebrafish brain. Click here for file

Additional data file 25 Expression of other miRNAs in the zebrafish brain. Click here for file

Additional data file 26 Legends of the figures presented in Additional data files 1-25 and 29 and the list of neuroanatomical abbreviations. Click here for file

Additional data file 27 Tables A-I providing detailed information about the expression of each miRNA in different structures of the zebrafish central and/or peripheral nervous system. Click here for file

Additional data file 28 Tables J-L, listing the miRNAs analyzed, the family/cluster they belong to and the mismatch locked nucleic acid (LNA) probe sequences used to test specificity of corresponding fully matching LNA probes. Click here for file

Additional data file 29 Mismatch test for *let-7a*, *miR-92b*, *miR-153a *and *miR-181a*. Click here for file
