# Supplementary material for: MicroRNAs show a wide diversity of expression profiles in the developing and mature central nervous system
Source: Genome Biol. 2007 Aug 21;8(8):R173. doi: 10.1186/gb-2007-8-8-r173 (PMC2375003; doi:10.1186/gb-2007-8-8-r173)
Supplement: Additional data file 26 — Legends of the figures presented in Additional data files 1-25 and 29 and the list of neuroanatomical abbreviations. [file gb-2007-8-8-r173-S26.doc]

**Additional data files 1-25 and 29 figure legends.**

**Additional data file 1. *miR-7* expression in the zebrafish brain.**

*miR-7* shows spatially localized expression, largely conserved throughout life. Larval expression is restricted to the forebrain in telencephalic, preoptic, few thalamic, eminentia thalami and ventral hypothalamic cells. In addition, it is expressed in a few cells of the area postrema at the dorsal junction of hindbrain-spinal cord (Table D). *miR-7* expression is mainly conserved in telencephalic, hypothalamic nuclei and area postrema between the larval and adult zebrafish brain. However, in the adult, we observe expression in the external and internal cellular layers of the olfactory bulb. Furthermore, in contrast to the larval, the adult thalamus is devoid of *miR-7* expression (Table D, I). *miR-7* often shares expression in the forebrain with *miR-222* but there are also some differences (table D, I).

A. parasagittal section through the larval fore- and midbrain showing *miR-7* expressing cells in the hypothalamus (caudal, Hc, intermediate, Hi and rostral, Hr), preoptic area (Po), eminentia thalami (ET), ventral subpallium (Sv) around the anterior commissure and pallium (P).

B. sagittal section through the larval fore- and midbrain showing *miR-7* expressing cells in the hypothalamus (caudal, Hc, intermediate, Hi and rostral, Hr), preoptic area (Po), eminentia thalami (ET), ventral (Sv) and dorsal (Sd) subpallium and pallium (P).

C. transverse section through the rostral larval telencephalon showing *miR-7* expressing cells in the ventral subpallium (Sv).

D. transverse section through the larval hypothalamus and midbrain showing *miR-7* expressing cells in the rostral ventral hypothalamus (Hr).

E. sagittal section through the larval hindbrain showing *miR-7* expressing cells in the caudal medulla oblongata (MO) at the level of the area postrema (AP).

F. transverse section through the adult olfactory bulb showing *miR-7* expressing cells in the internal (ICL) and external (ECL) cellular olfactory layers.

G. transverse section through the adult telencephalon showing *miR-7* expressing cells in the ventral (Vv), central (Vc) and dorsal (Vd) nuclei of the ventral telencephalon (subpallium), medial (Dm), central (Dc) and lateral (Dl) zones of the dorsal telencephalon (pallium). Expression is largely absent from cells lining the ventricles.

H. transverse section through the caudal adult telencephalon and rostral diencephalon (at the level of the optic chiasma, oc) showing *miR-7* expressing cells in the suprachiasmatic nucleus (SC), posterior parvocellular preoptic nucleus (PPp), dorsal endopeduncular nucleus (ENd, part of the eminentia thalami), posterior nucleus of the ventral telencephalon (Vp), posterior (Dp), medial (Dm), dorsal (Dd) and lateral (Dl) zones of the dorsal telencephalon.

I. transverse section through the caudal medulla oblongata (MO) showing *miR-7* expressing cells in the area postrema (AP).

J. transverse section through the adult hypothalamus showing *miR-7* expressing cells in the lateral hypothalamic nucleus (LH), ventral zone of the periventricular hypothalamus (Hv), dorsal zone of the periventricular hypothalamus at the level of the lateral hypothalamic ventricular recess (Hd-lr) and diffuse nucleus of the inferior hypothalamic lobe (DIL).

K. transverse section through the adult hypothalamus (caudal to section J) showing *miR-7* expressing cells in the caudal zone of the periventricular hypothalamus (Hc).

**Additional data files 2, 3, 4. *miR-9* expression in the zebrafish brain.**

*miR-9* is expressed in both periventricular and adjacent cells of proliferative zones and differentiating cells arising from these domains. *miR-9* expressing cells are widespread in all brain subdivisions (fore-, mid- and hind-brain) although some areas such as the larval epithalamus and the hypothalamic lateral torus are devoid of expression (Table A). In addition to the brain, *miR-9* is expressed in the inner retinal nuclear layer and ciliary marginal zone. *miR-9* expression is highly conserved between larval and adult brain and we find only minor differences at the regional level (Table A,F). *miR-9* is expressed in a few adult habenular and hypothalamic lateral torus cells, areas devoid of larval expression.

**Additional data file 2. *miR-9* expression in the 3dpf zebrafish brain.**

A. transverse section through the larval rostral telencephalon at the level of the olfactory epithelium (OE) showing *miR-9* expressing cells in the olfactory bulb (OB). Expression is absent in cells immediately ventral to the bulb.

B. transverse section through the larval telencephalon showing *miR-9* expressing cells in the ventral (Sv) and dorsal (Sd) subpallium, pallium (P) and olfactory bulb (OB).

C. transverse section through the larval caudal telencephalon and epithalamus showing *miR-9* expressing cells in the ventral (Sv) and dorsal (Sd) subpallium, pallium (P) and migrated telencephalic area (M4). Expression is absent from the habenulae and epiphysis.

D. transverse section through the larval diencephalon and rostral optic tectum showing *miR-9* expressing cells in the preoptic area (Po), eminentia thalami (ET), migrated eminentia thalami (M3), ventral thalamus (VT), dorsal thalamus (DT), zona limitans intrathalamica (ZLI) and the optic tectum (TeO).

E. transverse section through the larval diencephalon and optic tectum showing *miR-9* expressing cells in the preoptic area (Po), ventral thalamus (VT), dorsal thalamus (DT), periventricular (Pr) and migrated (M1) pretectal areas, optic tectum (TeO), retinal inner nuclear layer (INL) and ciliary marginal zone (arrowhead).

F. transverse section through the larval diencephalon and optic tectum showing *miR-9* expressing cells in the rostral hypothalamus (Hr), periventricular posterior tuberculum (PT), ventral thalamus (VT), dorsal thalamus (DT), periventricular (Pr) and migrated (M1) pretectal areas, proliferative (m) and periventricular gray (pgz) zones of the optic tectum (TeO).

G. transverse section through the larval diencephalon and optic tectum showing *miR-9* expressing cells in the rostral hypothalamus (Hr), ventral (PTv), dorsal (PTd) periventricular and migrated (M2) posterior tuberculum, dorsal thalamus (DT), periventricular pretectal area (Pr) and periventricular gray (pgz) zone of the optic tectum (TeO).

H. transverse section through the larval diencephalon and midbrain showing *miR-9* expressing cells in the rostral hypothalamus (Hr), ventral (PTv), dorsal (PTd) periventricular and migrated (M2) posterior tuberculum, tegmentum (T), proliferative (m) and periventricular gray (pgz) zones of the optic tectum (TeO).

I. transverse section through the larval hypothalamus and midbrain showing *miR-9* expressing cells in the intermediate hypothalamus (Hi, around the lateral ventricular recess, lr), ventral periventricular (PTv) and migrated (M2) posterior tuberculum, tegmental area at the level of the nucleus of the medial longitudinal fascicle (N), semicircular torus (TS) and periventricular gray zone (pgz) of the optic tectum (TeO).

J. transverse section through the larval hypothalamus and midbrain showing *miR-9* expressing cells in the intermediate hypothalamus (Hi) at the level of the lateral hypothalamic ventricular recess (lr), tegmental area (T), occulomotor nucleus (NIII), semicircular torus (TS) and periventricular gray zone (pgz) of the optic tectum (TeO). The arrowhead points at the oculomotor nerve (III).

K. transverse section through the larval caudal hypothalamus and midbrain showing *miR-9* expressing cells at the level of the lateral hypothalamic ventricular recess (lr), caudal hypothalamus (Hc), tegmental area (T), semicircular torus (TS), periventricular gray zone (pgz) of the optic tectum (TeO) and cerebellar valvula (Va).

L. transverse section through the larval caudal hypothalamus, midbrain and isthmus showing *miR-9* expressing cells in the interpeduncular nucleus (NIn), isthmic area (Is), semicircular torus (TS), cerebellar plate (CeP) and optic tectum (TeO).

M. transverse section through the larval hindbrain at the level of the octaval ganglion (OG) showing *miR-9* expressing cells in the medulla oblongata (MO) and cerebellar plate (CeP).

N. transverse section through the larval hindbrain at the level of the otic capsule (caudal to section M) showing *miR-9* expressing cells in the medulla oblongata (MO), presumptive octaval area (OA?) and rhombic lip (RL).

O. transverse section through the larval hindbrain at the level of the otic capsule (caudal to section N) showing *miR-9* expressing cells in the medulla oblongata (MO), presumptive octaval area (OA?) and rhombic lip (RL).

**Additional data file 3. *miR-9* expression in the 5dpf zebrafish brain.**

A. transverse section through the larval rostral telencephalon at the level of the olfactory epithelium (OE) showing *miR-9* expressing cells in the olfactory bulb (OB).

B. transverse section through the larval telencephalon at the level of the anterior commissure (ac) showing weakly *miR-9* expressing cells in the preoptic area (Po), dorsal subpallium (Sd), migrated telencephalic area (M4) and pallium (P).

C. transverse section through the larval caudal telencephalon and rostral diencephalon showing *miR-9* expressing cells in the preoptic area (Po), eminentia thalami (ET), migrated eminentia thalami (M3) and pallium (P).

D. transverse section through the larval diencephalon and rostral optic tectum showing *miR-9* expressing cells in the preoptic area, (Po), eminentia thalami (ET), migrated eminentia thalami (M3), ventral thalamus (VT), dorsal thalamus (DT), zona limitans intrathalamica (ZLI) and the optic tectum (TeO).

E. transverse section through the larval diencephalon and optic tectum showing *miR-9* expressing cells in the intermediate hypothalamus (Hi), ventral (PTv), dorsal (PTd) periventricular and migrated (M2) posterior tuberculum, dorsal thalamus (DT), periventricular pretectal area (Pr), proliferative zone (m), periventricular gray (pgz) zone and longitudinal torus (TL) of the optic tectum (TeO).

F. transverse section through the larval caudal hypothalamus and midbrain showing *miR-9* expressing cells at the level of the lateral hypothalamic ventricular recess (lr), caudal hypothalamus (Hc), tegmental area (T, at the level of the oculomotor nucleus, NIII), semicircular torus (TS), periventricular gray zone (pgz) of the optic tectum (TeO) and cerebellar valvula (Va). The arrowhead points at the oculomotor nerve.

G. transverse section through the larval caudal hypothalamus, midbrain and isthmus showing *miR-9* expressing cells in the caudal hypothalamus (Hc), diffuse nucleus of the hypothalamic inferior lobe (DIL), interpeduncular nucleus (NIn), isthmic area (Is), semicircular torus (TS), cerebellar plate (CeP), cerebellar valvula (Va) and optic tectum (TeO).

H. transverse section through the larval hindbrain at the level of the octaval ganglion (OG) showing *miR-9* expressing cells in the intermediate reticular formation (IMRF), medulla oblongata (MO), presumptive octaval area (OA?) and rhombic lip (RL).

I. transverse section through the larval hindbrain at the level of the posterior lateral line ganglion (PLLG) showing *miR-9* expressing cells in the inferior reticular formation (IRF), medulla oblongata (MO) and presumptive octaval area (OA?).

J. transverse section through the larval retina showing *miR-9* expressing cells in the inner nuclear layer (INL) and ciliary marginal zone (arrow).

**Additional data file 4. *miR-9* expression in the adult zebrafish brain.**

A. transverse section through the adult olfactory bulb showing *miR-9* expressing cells in the internal (ICL), external (ECL) cellular and glomerular (GL) olfactory layers.

B. transverse section through the adult telencephalon showing *miR-9* expressing cells in the ventral (Vv), lateral (Vl), central (Vc) and dorsal (Vd) nuclei of the ventral telencephalon (subpallium), medial (Dm), dorsal (Dd), central (Dc) and lateral (Dl) zones of the dorsal telencephalon (pallium).

C. higher magnification of B at the level of the subpallium showing *miR-9* expressing cells in the ventral (Vv), central (Vc) and dorsal (Vd) nuclei of the ventral telencephalon.

D. higher magnification of B at the level of the pallium and dorsal subpallium showing *miR-9* expressing cells in the dorsal nucleus of the ventral telencephalon (Vd), medial (Dm) and dorsal (Dd) zones of the dorsal telencephalon. Expression is present in many cells lining the ventricles in addition to presumptive neurons.

E. transverse section through the adult ventral telencephalon (caudal to sections B, C) showing periventricular (arrowheads) and lateral to the ventricle (arrows) *miR-9* expressing cells in the ventral (Vv) and dorsal (Vd) nuclei of the ventral telencephalon.

F. transverse section through the adult hypothalamus showing *miR-9* expressing cells in the dorsal zone of the periventricular hypothalamus (Hd). Arrowheads point at periventricular hypothalamic *miR-9* expressing cells at the level of the lateral ventricular recess (lr) and more medially.

G. transverse section through the adult dorsal diencephalon showing a few *miR-9* expressing cells in the dorsal habenular nucleus (Had), anterior (A) and ventromedial (VM) thalamic nuclei. Arrowheads point at periventricular *miR-9* expressing cells.

H. transverse section through the adult caudal preoptic area at the level of the postoptic commissure (poc) showing *miR-9* expressing cells in the posterior preoptic parvocellular nucleus (PPp). Blue arrowheads point at periventricular cells and black arrowheads point at *miR-9* expressing cells at the level of the poc.

I. transverse section through the adult caudal preoptic area at the level of the postoptic commissure (poc) showing *miR-9* expressing cells in the posterior parvocellular preoptic nucleus (PPp) and ventral zone of the periventricular hypothalamus (Hv).

J. transverse section through the adult diencephalon at the level of the posterior commissure (PC) showing *miR-9* expressing cells in the ventral nucleus of the periventricular pretectum (PPv), dorsal (DP) and central (CP) posterior thalamic nuclei, periventricular nucleus of posterior tuberculum (TPp), ventral zone of the periventricular hypothalamus (Hv), anterior (PGa) and lateral (PGl) preglomerular nuclei.

K. transverse section through the adult caudal midbrain and rostral hindbrain showing *miR-9* expressing cells in the perilemniscal nucleus (PL), nucleus of the lateral valvula (NLV), medial division of cerebellar valvula (Vam) and periventricular gray (pgz) and central (cz) zones of the optic tectum.

L. transverse section through the adult cerebellum showing *miR-9* expressing cells in the granular (CeGL), ganglionic (CeGaL) and molecular (CeML) cerebellar layers.

M. transverse section through the adult rostral hindbrain showing *miR-9* expressing cells in the superior reticular formation (SRF), superior raphe (SR), central gray (GC), nucleus of the lateral valvula (NLV) and granular cerebellar layer (CeGL).

N. transverse section through the adult hindbrain at the level of the octaval nerve (VIII) showing *miR-9* expressing cells in the intermediate reticular formation (IMRF), anterior octaval nucleus (AON), central gray (GC), granular (CeGL) and molecular (CeML) cerebellar layers.

O. transverse section through the adult rostral hindbrain at the level of the octaval nerve (VIII, caudal to section N) showing *miR-9* expressing cells in the intermediate reticular formation (IMRF), anterior (AON), secondary octaval (SO) and medial octavolateral (MON) nuclei, cerebellar crest (CC), granular (CeGL) and molecular (CeML) cerebellar layers.

P. transverse section through the adult hindbrain at the level of octaval nerve (VIII, caudal to section N) showing *miR-9* expressing cells in the inferior reticular formation (IRF), descending octaval (DON) and caudal octavolateral (CON) nuclei and facial lobe (LVII). The arrowhead points at periventricular cells around the rhombencephalic ventricle expressing *miR-9*.

Q. transverse section through the adult caudal hindbrain at the level of the vagal motor nucleus (NXm) showing *miR-9* expressing cells surrounding the rhombencephalic ventricle (rv), in the facial (LVII) and vagal (LX) lobes and inferior reticular formation (IRF).

R. transverse section through the adult caudal hindbrain at the level of the area postrema (AP) showing *miR-9* expressing cells in the lateral part of the commissural nucleus of Cajal (NC) and surrounding (arrowheads) the rhombencephalic ventricle and medial longitudinal fascicle (MLF).

**Additional data file 5. *miR-34* expression in the zebrafish brain.**

*miR-34* shows very restricted expression in the larval brain with transcription restricted to ventral and lateral larval hindbrain areas, *miR-34* conserves its expression in the adult hindbrain but additionally shows de novo expression in rostral and caudal nuclei, and thus changes dramatically its expression.

Specifically, at 3dpf and 5dpf, *miR-34* is expressed in ventral and lateral medulla oblongata cells, (presumptive octaval area), locus coeruleus, trigeminal motor nuclei cells, Mauthner, and reticular formation cells (Table D). In the adult brain, *miR-34* conserves its expression in respective nuclei and expands in specific nuclei and distinct cells of rostral brain, including epithalamic, pretectal, posterior tuberculum, tectal and tegmental cells, as well as in areas of dorsal hindbrain (cerebellar granular layer, facial and vagal lobes, Table I).

A. transverse section through the larval rostral hindbrain at the level of the octaval ganglion (OG) showing *miR-34* expressing cells in the ventral medulla oblongata (MO), superior reticular formation (SRF), likely the trigeminal motor nucleus (NV?) and octaval ganglion (OG).

B. transverse section through the larval hindbrain at the level of the otic capsule (ot, caudal to section A) showing *miR-34* expressing cells in the ventrolateral medulla oblongata (MO, likely octaval area, OA) and intermediate reticular formation (IMRF).

C. transverse section through the caudal larval hindbrain at the level of the inferior olive (IO) showing *miR-34* expressing cells in the ventrolateral medulla oblongata (MO, arrowhead).

D. transverse section through the larval hindbrain at the level of the octaval ganglion (OG) showing *miR-34* expressing cells in the ventrolateral medulla oblongata (MO, likely octaval area, OA), intermediate reticular formation (IMRF), Mauthner neuron (MAC) and octaval ganglion (OG).

E. transverse section through the larval hindbrain at the level of the octaval ganglion (OG, section caudal to section D) showing *miR-34* expressing cells in the ventrolateral medulla oblongata (MO), likely octaval area (OA), intermediate reticular formation (IMRF) and octaval ganglion (OG). The arrowhead points at *miR-34* expressing cells in the ventral hindbrain in proximity to the octaval nerve (VIII).

F. transverse section through the caudal larval hindbrain at the level of the inferior olive (IO) showing *miR-34* expressing cells (arrowhead) in the ventrolateral medulla oblongata (MO), likely ventral to the vagal motor nucleus (NX).

G. transverse section through the adult epithalamus showing *miR-34* expressing cells (arrowhead) in the dorsal habenular nucleus (Had).

H. transverse section through the adult hypothalamus showing *miR-34* expressing cells in the nucleus of the paraventricular organ (nPVO).

I. transverse section through the adult cerebellum showing *miR-34* expressing cells in the cerebellar granular layer (CeGL).

J. transverse section through the adult rostral hindbrain showing *miR-34* expressing cells in the locus coeruleus (LC) and superior reticular formation (SRF).

K. transverse section through the adult hindbrain at the level of the trigeminal root showing *miR-34* expressing cells in the primary sensory trigeminal nucleus (NVs), dorsal part of trigeminal motor nucleus (NVmd) and superior reticular formation (SRF).

L. transverse section through the adult lateral hindbrain at the level of the Mauhtner neuron (MAC) showing *miR-34* expressing cells in the MAC, magnocellular (MaON), anterior (AON) octaval and medial octavolateral (MON) nuclei.

M. transverse section through the adult superficial pretectum showing *miR-34* expressing cells in the magnocellular superficial pretectal nucleus (PSm) and the optic tectum (TeO).

N. transverse section through the adult pretectum and optic tectum showing *miR-34* expressing cells in the tectal periventricular grey (pgz) and central (arrowhead) zones and accessory pretectal nucleus (APN).

O. transverse section through the adult lateral hindbrain at the level of the octaval nerve (VIII, caudal to section L) showing *miR-34* expressing cells in the descending octaval (DON) and medial octavolateral (MON) areas and the intermediate reticular formation (IMRF). The arrowhead points at *miR-34* expressing cells in the ventral hindbrain in proximity to the octaval nerve (VIII).

P. transverse section through the adult lateral hindbrain at the level of the vagal nerve (X), showing *miR-34* expressing cells in the facial lobe (LVII), posterior octaval area (PON) and lateral to the medial longitudinal fascicle (MLF).

**Additional data file 6. *miR-92b* expression in the zebrafish brain.**

*miR-92b* is predominantly expressed in periventricular and adjacent cells corresponding to proliferative zones throughout the 5dpf larval brain (table A). It is also expressed in the retinal ciliary marginal zone. *miR-92b* expression is conserved in the same cell types in the adult brain (table F).

A. transverse section through the larval telencephalon showing *miR-92b* expressing cells in the ventral (Sv) and dorsal (Sd) subpallium, pallium (P) and epiphysis (E).

B. transverse section through the caudal telencephalon and rostral diencephalon showing *miR-92b* strongly expressing cells lining and close to the ventricle between the pallium (P) and epithalamus (habenula-Ha and epiphysis-E).

C. transverse section through the larval diencephalon and rostral optic tectum showing *miR-92b* expressing cells lining and close to the ventricle in the preoptic area, (Po), eminentia thalami (ET), ventral thalamus (VT), dorsal thalamus (DT) and the optic tectum (TeO).

D. transverse section through the larval diencephalon and optic tectum showing *miR-92b* expressing cells lining and close to the ventricle in the preoptic area (Po), ventral thalamus (VT), dorsal thalamus (DT), periventricular pretectum (Pr) and the proliferative zone of the optic tectum (m).

E. oblique transverse section through the larval midbrain, hypothalamus and rostral isthmus showing *miR-92b* expressing cells in the intermediate hypothalamus (Hi) around the lateral ventricular recess (lr, arrowheads), ventral posterior periventricular tuberculum (PTv), lining and close to the ventricle of tegmentum (T) and semicircular torus (TS), in the cerebellar valvula (Va) and proliferative zone of the optic tectum (m).

F. transverse section through the larval midbrain, hypothalamus and rostral isthmus showing *miR-92b* expressing cells in the intermediate hypothalamus (Hi) around the lateral ventricular ventricular recess (lr), lining and close to the ventricle in the tegmentum and isthmus (T/Is), semicircular torus (TS), in the cerebellar valvula (Va), cerebellar plate (CeP) and proliferative zone of the optic tectum (m).

G. transverse section through the adult telencephalon showing *miR-92b* expressing cells lining the telencephalic ventricle (tv) in the subpallium (dorsal nucleus of the ventral telencephalon,Vd) and the pallium (medial nucleus of the dorsal telencephalic area, Dm).

H. transverse section through the adult midbrain and hypothalamus showing *miR-92b* expressing cells lining/close to the ventricle in the dorsal (Hd-lr) and caudal (Hc) zones of the periventricular hypothalamus, mammillary body (CM) and lateral torus (TLa).

I. transverse section through the larval retina showing *miR-92b* expressing cells in the ciliary marginal zone (arrow).

J. transverse section through the adult dorsal diencephalon showing *miR-92b* expressing cells lining/close to the ventricle in the dorsal (Had) and ventral (Hav) habenular nuclei, anterior (A) and ventromedial (VM) thalamic nuclei.

K. transverse section through the adult cerebellum, isthmus and caudal midbrain. Arrows point at *miR-92b* expressing cells lining/close to the ventricle in the central gray (GC), optic tectum (TeO), central semicircular torus (TSc) and cerebellum (CCe).

**Additional data file 7. *miR-124* expression in the zebrafish brain**

*miR-124* is expressed in virtually all differentiating cells throughout the 3dpf, 5dpf brain and retina and conserved in similar domains in the adult brain (Table B, G).

A. transverse section through the larval telencephalon and rostral epithalamus showing *miR-124* expressing cells in the ventral (Sv) and dorsal (Sd) subpallium, pallium (P), olfactory bulb (OB) and habenula (Ha). Expression is absent from ventricular and periventricular cells.

B. transverse section through the larval caudal telencephalon and epithalamus showing *miR-124* expressing cells in the ventral (Sv) and dorsal (Sd) subpallium, pallium (P), migrated telencephalic area (M4), habenula (Ha) and epiphysis (E).

C. transverse section through the larval diencephalon and rostral optic tectum showing *miR-124* expressing cells in the preoptic area, (Po), eminentia thalami (ET), migrated eminentia thalami (M3), ventral thalamus (VT), dorsal thalamus (DT) and the optic tectum (TeO).

D. transverse section through the larval diencephalon and midbrain showing *miR-124* expressing cells in the rostral hypothalamus (Hr), periventricular posterior tubercular area (PT), dorsal thalamus (DT), periventricular pretectum (Pr) and periventricular gray zone (pgz) of the optic tectum (TeO).

E. transverse section through the larval diencephalon and midbrain showing *miR-124* expressing cells in the rostral hypothalamus (Hr), periventricular (dorsal-PTd, ventral-PTv) and migrated (M2) posterior tubercular area, tegmental area (T, at the level of the nucleus of medial longitudinal fascicle, N) and periventricular gray zone (pgz) of the optic tectum (TeO).

F. transverse section through the larval retina (dorsal to the right) showing *miR-124* expressing cells in the photoreceptor (Ph), inner nuclear (INL) and ganglion cell (GCL) layers. Expression is absent from the ciliary marginal zone (CMZ).

G. transverse section through the larval hypothalamus and midbrain showing *miR-124* expressing cells in the intermediate hypothalamus (Hi), migrated posterior tubercular area (M2), tegmental area (T, at the level of the oculomotor nucleus NIII), semicircular torus (TS) and periventricular gray zone (pgz) of the optic tectum (TeO).

H. transverse section through the larval hypothalamus, midbrain and isthmus showing *miR-124* expressing cells in the intermediate hypothalamus (Hi), isthmic area (Is), cerebellar plate (CeP), semicircular torus (TS) and optic tectum (TeO).

I. transverse section through the hindbrain at the level of the posterior lateral line (PLLG) and vagal (VG) ganglia showing *miR-124* expressing cells in the medulla oblongata (MO) and inferior raphe (IR).

J. transverse section through the adult telencephalon showing *miR-124* expressing cells in the subpallium (ventral nucleus of the ventral telencephalon,Vv, lateral nucleus of the ventral telencephalon, Vl, dorsal nucleus of the ventral telencephalon,Vd) and the pallium (medial zone of the dorsal telencephalic area, Dm, posterior zone of the dorsal telencephalic area, Dp).

K. higher magnification of J at the level of ventral (Vv) and dorsal (Vd) nuclei of the ventral telencephalon, showing that *miR-124* expressing cells are lateral to the ventricle (blue) whereas cells lining the ventricle are devoid of labelling (red, arrowheads).

L. transverse section through the adult hypothalamus showing *miR-124* expressing cells in the lateral hypothalamic nucleus (LH), anterior tuberal nucleus (ATN), nucleus of the paraventricular organ (nPVO), posterior tuberal nucleus (PTN) and nucleus of posterior periventricular tuberculum (TPp).

**Additional data file 8. *miR-128* expression in the zebrafish brain.**

*miR-128* shows spatially localized, conserved expression in specific larval and adult brain nuclei or cells. In addition, *miR-128* expression shows major quantitative differences in levels of expression between structures in the larval brain.

At 5dpf, *miR-128* is expressed in the entire olfactory bulb, pallium, and lateral medulla oblongata. In these regions, it is upregulated in the olfactory bulb, caudal pallial and lateral medulla oblongata cells. It is also expressed in distinct cells in the habenula, ventral thalamus, posterior tuberculum, lateral hypothalamus, periventricular and central tectal zones, semicircular torus and area postrema. It conserves its expression in these areas in the adult brain and given the good overall regional similarities in *miR-128* expression between 5dpf and adult brain we suggest that the 5dpf expression in the medulla oblongata corresponds to cells of the presumptive octaval area, facial and vagal lobes, area postrema and commissural nucleus of Cajal. One difference in the expression pattern of *miR-128* between larval and adult brain is expression in the adult inferior olive (Tables C, H).

A. transverse section through the rostral larval telencephalon showing *miR-128* expressing cells in the olfactory bulb (OB) and pallium (P).

B. transverse section through the caudal telencephalon and epithalamus showing *miR-128* expressing cells in the preoptic area (Po), dorsal subpallium (Sd), migrated telencephalic area (M4), pallium (P) and habenula (Ha).

C. transverse section through the larval diencephalon and optic tectum showing *miR-128* expressing cells in the lateral eminentia thalami (ET, arrowhead), ventral thalamus (VT), migrated pretectal area (M1), periventricular gray zone (pgz) and longitudinal torus (TL) of the optic tectum.

D. transverse section through the larval hypothalamus and midbrain showing *miR-128* expressing cells in the rostral hypothalamus (Hr), migrated posterior tubercular area (M2), semicircular torus (TS), periventricular gray (pgz) and central zone (cz) of the optic tectum (TeO).

E. transverse section through the larval hypothalamus and midbrain showing *miR-128* expressing cells in the rostral hypothalamus (Hr), lateral torus (TLa), ventral periventricular (PTv) and migrated posterior tubercular area (M2), semicircular torus (TS), periventricular gray (pgz) and central zone (cz) of the optic tectum (TeO).

F. transverse section through the larval hindbrain at the level of the otic capsule (ot) showing *miR-128* expressing cells in the lateral medulla oblongata (MO), including the octaval area (OA).

G. transverse section through the larval hindbrain at the level of the octaval ganglion (OG) showing *miR-128* expressing cells in the lateral medulla oblongata (MO), including the octaval area (OA).

H. transverse section through the larval hindbrain caudal to the posterior lateral line ganglion showing *miR-128* expressing cells in the lateral medulla oblongata (MO) likely in the octaval area (OA) and vagal lobe (LX).

I. transverse section through the larval caudal medulla oblongata (MO) showing *miR-128* expressing cells in the area postrema (AP) and likely the commissural nucleus of Cajal (NC).

J. transverse section through the rostral adult telencephalon showing *miR-128* expressing cells in the dorsal telencephalon/pallium (P) and olfactory bulb (OB) with the exception of the area of the medial olfactory tract (MOT).

K. transverse section through the adult epithalamus and thalamus showing *miR-128* in the dorsal part of the ventral habenula (Hav) and ventrolateral thalamic nucleus (VL).

L. transverse section through the adult hypothalamus and midbrain showing *miR-128* expressing cells in the lateral hypothalamic nucleus (LH), anterior tuberal nucleus (ATN), dorsal zone of the periventricular hypothalamus (Hd), diffuse nucleus of the inferior hypothalamic lobe (DIL), lateral torus (TLa), posterior tuberal nucleus (PTN), medial preglomeral nucleus (PGm), posterior thalamic nucleus (Pt) and optic tectum (TeO). For higher magnifications of the hypothalamus and optic tectum, see pictures M and O.

M. transverse section through the adult midbrain and isthmus (caudal to section L) showing *miR-128* expressing cells in the isthmic nucleus (NI), central nucleus of semicircular torus (TSc), periventricular gray (pgz) and superficial grey (sgz) zones of the optic tectum. The asterisk marks the proliferative tectal zone devoid of *miR-128* expression.

N. transverse section through the adult hindbrain at the level of the octaval nerve (VIII) showing *miR-128* expressing cells in the medial octavolateral nucleus (MON), cerebellar granular eminence (EG) and granular cerebellar layer (CeGL).

O. higher magnification of section L at the level of the adult hypothalamus showing *miR-128* expressing cells in the lateral hypothalamic nucleus (LH), anterior tuberal nucleus (ATN), dorsal zone of the periventricular hypothalamus (Hd), diffuse nucleus of the inferior hypothalamic lobe (DIL), lateral torus (TLa), posterior tuberal nucleus (PTN), medial preglomeral nucleus (PGm) and posterior thalamic nucleus (Pt).

P. transverse section through the adult hindbrain at the level of the vagal nerve (X, section caudal to R) showing *miR-128* expressing cells in the inferior olive (IO), caudal octavolateral nucleus (CON) and vagal lobe (LX).

Q. transverse section through the adult hindbrain at the junction with the spinal cord (section caudal to P) showing *miR-128* expressing cells in the area postrema (AP), commissural nucleus of Cajal (NC) and medial funicular nucleus (MFN).

R. transverse section through the adult hindbrain at the level of the cerebellar crest (CC, section caudal to N) showing *miR-128* expressing cells in the descending octaval nucleus (DON), caudal octavolateral nucleus (CON) and facial lobe (LVII).

**Additional data file 9. *miR-135c* expression in the zebrafish brain.**

*miR-135c* is expressed in both periventricular and differentiating cells with a more restricted pattern compared to *miR-9*. *miR-135c* expression is mainly conserved between larval and adult brain but also shows some differences (Tables B,G). *miR-135c* expression is conserved in both larval and adult subpallium, caudal pallium, habenular, preoptic, thalamic, hypothalamic, pretectal, tegmental, cerebellar, isthmic and octaval nuclei. It is expressed in additional adult areas, devoid of larval expression, such as the caudal hypothalamus, periventricular and migrated posterior tuberculum nuclei, inferior raphe and reticular formation. In other hindbrain areas, it is difficult to draw conclusion about the conservation of *miR-135c* expression from larval to adult stages: *miR-135c* is robustly expressed in medial and central columns throughout the larval isthmic area and medulla oblongata but this is not the case in the adult. *miR-135c* is expressed in many cells of the adult facial and vagal lobes, and this expression may correspond to part of the larval one.

A. transverse section through the larval telencephalon showing *miR-135c* expressing cells in the ventral (Sv) and dorsal (Sd) subpallium.

B. transverse section through the larval diencephalon and rostral optic tectum showing *miR-135c* expressing cells in the preoptic area (Po), eminentia thalami (ET), ventral (VT) and dorsal thalamus (DT).

C. transverse section through the larval caudal midbrain, hypothalamus, cerebellum and isthmus/medulla oblongata showing *miR-135c* expressing cells in the isthmic nucleus (NI), cerebellar plate (CeP), optic tectum (TeO) and isthmic/medulla oblongata areas (Is/MO).

D. transverse section through the larval hindbrain at the level of the octaval ganglion (OG) showing *miR-135c* expressing cells in the medulla oblongata (MO).

E. transverse section through the larval hindbrain at the level of the posterior lateral line ganglion (PLLG) showing *miR-135c* expressing cells in the medulla oblongata (MO) likely including the presumptive octaval area (OA).

F. transverse section through the adult telencephalon showing *miR-135c* expressing cells lining or lateral to the telencephalic ventricle in the subpallium (ventral nucleus of the ventral telencephalon,Vv, dorsal nucleus of the ventral telencephalon,Vd).

G. transverse section through the adult caudal telencephalon showing *miR-135c* expressing cells in the posterior zone of the dorsal telencephalon (Dp) and ventral part of the entopeduncular nucleus (ENv).

H. transverse section through the adult dorsal diencephalon showing *miR-135c* expressing cells lining or close to the diencephalic ventricle (dv) in the ventral habenular nucleus (Hav), intermediate (I), ventromedial (VM) and ventrolalateral (VL) thalamic nuclei.

I. transverse section through the adult midbrain and hypothalamus showing *miR-135c* expressing cells lining/close to the ventricle in the dorsal (Hd-lr, around the lateral ventricular recess) and caudal (Hc) zones of the periventricular hypothalamus.

J. transverse section through the adult caudal midbrain and isthmus showing *miR-135c* expressing cells in the tectal periventricular gray zone (pgz), periventricular central semicircular torus (arrow, TSc) nucleus of lateral valvula (NLV) and isthmic nucleus (NI).

K. transverse section through the adult isthmus showing *miR-135c* expressing cells in the inferior reticular formation (IRF).

L. transverse section through the caudal medulla oblongata showing *miR-135c* expressing cells in the inferior reticular formation (IRF), caudal octavolateral nucleus (CON), facial lobe (LVII) and surrounding the rhombencephalic ventricle (rv, arrows).

**Additional data file 10. *miR-137* expression in the zebrafish brain.**

*miR-137* shows spatially localized, conserved expression in specific larval and adult brain nuclei or cells (Table C, H).  *miR-137* expression is conserved in many larval and adult areas including the subpallium, ventro-medial and caudal pallium, preoptic area, dorsal thalamus, hypothalamus and ventral posterior tubercular area. Given the good correspondence of localized expression between larval and adult brains, this allows us to annotate specific nuclei in the larval tegmentum, isthmus and medulla oblongata. We suggest that the cells in the larval tegmentum and medulla oblongata correspond to the midbrain dorsal tegmental nucleus, lateral nucleus of cerebellar valvula, isthmic nucleus, facial and glossopharyngeal/vagal lobes, vagal motor nucleus and area postrema. Despite the well-conserved pattern of expression between larval and adult zebrafish brain, we observe minor differences. *miR-137* is expressed in adult dorsal lateral habenular cells and a few tectal periventricular gray zone and migrated posterior tuberculum cells.

A. transverse section through the larval telencephalon showing *miR-137* expressing cells in the ventral (Sv) and dorsal (Sd) subpallium and pallium (P).

B. transverse section through the caudal telencephalon and epithalamus showing *miR-137* expressing cells in the ventral subpallium(Sv)/preoptic area (Po), dorsal subpallium (Sd), migrated telencephalic area (M4) and pallium (P).

C. transverse section through the caudal telencephalon and epithalamus showing *miR-137* expressing cells in the pallium (P) and eminentia thalami (ET).

D. transverse section through the larval diencephalon and rostral optic tectum showing *miR-137* expressing cells in the eminentia thalami (ET), ventral thalamus (VT) and dorsal thalamus (DT).

E. transverse section through the larval diencephalon and rostral optic tectum (caudal to section D) showing *miR-137* expressing cells in the eminentia thalami (ET), ventral thalamus (VT) and dorsal thalamus (DT).

F. transverse section through the larval diencephalon and midbrain showing *miR-137* expressing cells in the rostral hypothalamus (Hr), dorsal periventricular posterior tuberculum (PTd) and dorsal thalamus (DT).

G. transverse section through the larval diencephalon and midbrain showing *miR-137* expressing cells in the intermediate hypothalamus (Hi), lateral hypothalamic torus (TLa) and ventral periventricular posterior tuberculum (PTv).

H. transverse section through the larval diencephalon and midbrain showing *miR-137* expressing cells in the lateral hypothalamic torus (TLa), ventral periventricular posterior tuberculum (PTv) and midbrain dorsal tegmental nucleus (DTN).

I. transverse section through the larval caudal midbrain and rostral hindbrain at the level of the facial ganglion (FG) showing *miR-137* expressing cells in the isthmic area (Is) including the isthmic nucleus (NI).

J. transverse section through the adult telencephalon at the level of the anterior commissure (ac) showing *miR-137* expressing cells in the supracommissural nucleus of the ventral (subpallial) telencephalic area (Vs), posterior (Dp), lateral (Dl), dorsal (Dd), medial (Dm) zones of the dorsal (pallial) telencephalic area and the dorsal entopeduncular nucleus (ENd).

K. transverse section through the adult caudal telencephalon at the level of the optic chiasma (oc) showing *miR-137* expressing cells in the posterior (Dp), lateral (Dl) and medial (Dm) zones of the dorsal (pallial) telencephalic area, posterior preoptic parvocellular (PPp) and suprachiasmatic (SC) nuclei.

L. transverse section through the adult epithalamus showing *miR-137* expressing cells in the dorsal habenular nucleus (Had, arrowheads).

M. transverse section through the adult diencephalon showing *miR-137* expressing cells in the ventral zone of the hypothalamus (Hv), anterior tuberal nucleus (ATN), dorsal zone of the periventricular hypothalamus (Hd), lateral torus (TLa), posterior tuberal nucleus (PTN), medial preglomeral nucleus (PGm), posterior thalamic nucleus (Pt) and central posterior thalamic nucleus (CP). For higher magnification of the thalamus, see section N.

N. Higher magnification of a transverse section (level caudal to section M) through the adult dorsal diencephalon, at the level of the posterior commissure (pc) showing *miR-137* expressing cells in the central posterior thalamic nucleus (CP).

O. transverse section through the young adult diencephalon and midbrain showing *miR-137* expressing cells in the ventral zone of the hypothalamus (Hv), lateral hypothalamic nucleus (LH), dorsal zone of the periventricular hypothalamus around the lateral hypothalamic ventricular recess (Hd-lr), diffuse nucleus of inferior lobe (DIL), lateral torus (TLa), posterior tuberal nucleus (PTN), ventral part of the nucleus of the posterior periventricular tuberculum (TPp), posterior thalamic nucleus/lateral preglomeral nucleus (Pt/PGl) and midbrain dorsal tegmental nucleus (DTN).

P. Higher magnification of the dorsal part of the transverse section P showing *miR-137* expressing cells in the midbrain dorsal tegmental nucleus (DTN), ventral part of the nucleus of the posterior periventricular tuberculum (TPp) and a few cells in the proximity of the mesencephalic ventricle (arrowhead).

Q. transverse section through the young adult caudal hypothalamus, midbrain and isthmus showing *miR-137* expressing cells in the caudal zone of the hypothalamus (Hc), diffuse nucleus of the hypothalamic inferior lobe (DIL), isthmic nucleus (NI), nucleus of lateral cerebellar valvula (NLV), central nucleus of semicircular torus (TSc) and optic tectum (TeO).

R. transverse section through the adult caudal hindbrain at the level of the vagal nerve showing *miR-137* expressing cells in the vagal lobe (LX) and vagal motor nucleus (NXm).

S. transverse section through the adult hindbrain at the junction with the spinal cord (caudal to section R) showing *miR-137* expressing cells in the area postrema (AP) and commissural nucleus of Cajal (NC).

**Additional data file 11. *miR-138* expression in the zebrafish brain**

*miR-138* is expressed in differentiated cells with more restriction compared to *miR-124*. Expression is largely conserved between larval and adult brain but there are some differences (Tables B,G). With the exception of the medulla oblongata where *miR-138* larval expression is widespread and thus difficult to correlate with the adult, we observe the following similarities and differences between larval and young adult expression: *miR-138* is expressed in both larval and young adult brains in the olfactory bulb, pallial and subpallial areas, preoptic area, dorsolateral habenular cells, dorsal posterior thalamic nuclei, hypothalamic region, tectal cells, isthmic area and cerebellar cells. Furthermore it is expressed in the adult medial octavolateral nucleus, facial and vagal lobe and reticular formation. This adult hindbrain expression may partially correspond to the larval expression in lateral isthmus and central and medial part of the medulla oblongata.  *miR-138* is not expressed in the young adult ventral thalamus and semicircular torus whereas it is expressed in these areas at 5dpf. Conversely, it is expressed in adult migrated posterior tuberculum areas, migrated pretectal nuclei, longitudinal torus and cerebellar valvula cells, areas devoid of staining in the larval brain.

A. transverse section through the larval telencephalon showing *miR-138* expressing cells in the ventral (Sv) and dorsal (Sd) subpallium, pallium (P) and olfactory bulb (OB).

B. oblique transverse section through the larval caudal telencephalon and epithalamus showing *miR-138* expressing cells in the preoptic area (Po), dorsal subpallium (Sd), pallium (P) and habenula (Ha).

C. transverse section through the larval diencephalon and optic tectum showing *miR-138* expressing cells in the ventral thalamus (VT), dorsal thalamus (DT) and the periventricular gray zone of the optic tectum (pgz).

D. transverse section through the adult olfactory bulb showing *miR-138* expressing cells in the internal granular layer (ICL).

E. transverse section through the young adult cerebellum showing *miR-138* expressing cells in the cerebellar ganglionic layer (CeGAL).

F. transverse section through the young adult telencephalon showing *miR-138* expressing cells in the subpallium (ventral nucleus of the ventral telencephalon, Vv, dorsal nucleus of the ventral telencephalon,Vd) and the pallium (medial zone of the dorsal telencephalic area, Dm).

G. transverse section through the young adult diencephalon showing *miR-138* expressing cells in the ventral periventricular zone of the hypothalamus (Hv), dorsal habenular nucleus (Had), parvocellular superficial (PSp) and central (CPN) pretectal nuclei.

H. transverse section through the larval isthmus, hypothalamus and caudal midbrain showing *miR-138* expressing cells in the lateral hypothalamic torus (TLa), isthmic area (Is), and cerebellar plate (CeP).

**Additional data file 12. *miR-153a* expression in the zebrafish brain.**

*miR-153a* shows expression in both periventricular and differentiating cells in the larval brain and has more restricted expression compared to *miR-124*. It is expressed in periventricular cells only in a few cases. In addition, *miR-153a* expression shows quantitative differences from one area to another and is conserved in many areas between larval and adult rostral brain but also presents some regional differences (Tables C,H).

Rostrally, *miR-153a* is expressed in both larval and adult telencephalic pallial and subpallial areas, preoptic area, habenula, dorsal and ventral thalamus, hypothalamus, posterior tuberculum, mesencephalic tectal periventricular gray zone and interpeduncular nucleus. In addition to these similarities between rostral larval and adult brain, we observe some differences: *miR-153a* is expressed only in the adult olfactory bulb whereas it is expressed only in the larval periventricular pretectum. Furthermore, it is expressed in the entire larval tegmentum but in the adult it is restricted in the semicircular torus. Caudally, *miR-153a* is expressed in the young adult and adult hindbrain, in the isthmic nucleus, nucleus of lateral valvula, perilemniscal nucleus, facial and vagal lobes and area postrema. Within the larval hindbrain we observe columns of *miR-153a* expression but it is difficult to establish a precise correlation with the adult nuclei. Finally, we observe quantitative differences in *miR-153a* expression between regions. For example *miR-153a* expression is particularly highly expressed in the larval habenula and migrated eminentia thalami.

A. transverse section through the larval telencephalon showing *miR-153a* in the ventral (Sv) and dorsal (Sd) subpallium and pallium (P).

B. transverse section through the larval caudal telencephalon and diencephalon showing *miR-153a* expressing cells in the preoptic area (Po), eminentia thalami (ET), migrated eminentia thalami (M3), pallium (P) and habenula (Ha).

C. transverse section through the larval diencephalon and rostral optic tectum showing *miR-153a* expressing cells in the preoptic area (Po), ventral thalamus (VT), dorsal thalamus (DT), periventricular pretectum (Pr) and periventricular gray zone (pgz) of the optic tectum (TeO).

D. transverse section through the larval diencephalon and midbrain showing *miR-153a* expressing cells in the rostral hypothalamus (Hr), periventricular (PT) and migrated (M2) posterior tubercular area, dorsal thalamus (DT) and periventricular gray zone (pgz) of the optic tectum (TeO).

E. transverse section through the larval caudal hypothalamus, midbrain and rostal hindbrain showing *miR-153a* expressing cells in the intermediate (Hi) and caudal (Hc) hypothalamus, diffuse nucleus of inferior lobe (DIL), interpeduncular nucleus (NIn), tegmentum (T), oculomotor nucleus (NIII), semicircular torus (TS) and optic tectum (TeO).

F. transverse section through the larval rostal hindbrain and caudal hypothalamus showing *miR-153a* expressing cells in the central medulla oblongata (MO), isthmic nucleus (NI), superior raphe (SR) and caudal hypothalamus (Hc).

G. transverse section through the larval hindbrain at the level of the octaval ganglion (OG) showing *miR-153a* expressing cells in the central medulla oblongata (MO).

H. transverse section through the rostral adult olfactory bulb showing *miR-153a* expressing cells in the glomerular (GL) and external cellular (ECL) layers.

I. transverse section through the adult olfactory bulb (caudal to section H) showing *miR-153a* expressing cells in the glomerular (GL), external (ECL) and internal (ICL) cellular layers.

J. transverse section through the adult telencephalon showing *miR-153a* expressing cells in the ventral (Vv), lateral (Vl), central (Vc) and dorsal (Vd) nuclei of the ventral telencephalon (subpallium), medial (Dm), central (Dc), lateral (Dl) and posterior (Dp) zones of the dorsal telencephalon (pallium).

K. higher magnification of J through the adult dorsal telencephalon showing strongly *miR-153a* expressing cells along the dorsal part of the telencephalic ventricle (tv, asterisk) and the lateral part of the medial zone of the dorsal telencephalon (Dm) and few weakly expressing *miR-153a* cells along the medial ventricular wall (arrowheads).

L. higher magnification of J through the adult medial telencephalic part, ventral to section K, showing strongly *miR-153a* expressing cells in the lateral part of Dm (medial zone of dorsal telencephalon), central nucleus of the ventral telencephalon (Vc) and weakly expressing cells along the medial ventricle in the dorsal nucleus of the ventral telencephalon (Vd, arrowhead).

M. transverse section through the adult epithalamus showing *miR-153a* expressing cells in the dorsal (Had) and ventral (Hav) habenular nuclei.

N. transverse section through the young adult hypothalamus showing *miR-153a* expressing cells in the lateral hypothalamic nucleus (LH), anterior tuberal nucleus (ATN), dorsal zone of the periventricular hypothalamus (Hd), diffuse nucleus of the inferior hypithalamic lobe (DIL), lateral torus (TLa), nucleus of the paraventricular organ (nPVO), medial preglomeral nucleus (PGm) and periventricular nucleus of posterior tuberculum (TPp).

O. transverse section through the adult tegmentum at the level of the oculomotor nucleus (NIII) showing *miR-153a* expression only in the lateral hypothalamic torus (TLa).

P. transverse section through the adult hypothalamus showing *miR-153a* expressing cells in the lateral hypothalamic nucleus (LH), anterior tuberal nucleus (ATN), ventral zone of the periventricular hypothalamus (Hv), dorsal zone of the periventricular hypothalamus (Hd), diffuse nucleus of the inferior hypothalamic lobe (DIL), lateral torus (TLa), nucleus of the paraventricular organ (nPVO) and posterior tuberal nucleus (PTN).

Q. transverse section through the young adult isthmus and caudal midbrain showing *miR-153a* expressing cells in the semicircular torus (TS), tectal periventricular gray zone (pgz) and istmic nucleus (NI).

R. transverse section through the adult caudal hindbrain showing *miR-153a* expressing cells in the facial (LVII) and vagal (LX) lobes.

S. transverse section through the adult caudal hindbrain (ventral to section Q) showing *miR-153a* expressing cells in the inferior olive (IO).

**Additional data files 13 and 14. *miR-181a* and *miR-181b* expression in the zebrafish brain.**

*miR-181a* and *miR-181b* belong to the same *miR*NA family and differ in three nucleotides located outside the seed region. These *miR*NAs show similar, spatially localised expression in the larval brain. *miR-181a* and *miR-181b* expression is quantitatively different from one area to another in the larval brain with strong expression in the retina and brain areas associated with the visual system. *miR-181a* and *miR-181b* expression is largely conserved to adult stage although there is downregulation in some areas. Despite overall conservation, we noticed subtle differences in the adult expression of *miR-181a* and *miR-181b* that were not obvious at larval stages.

In the larval brain, *miR-181a* and *miR-181b* are strongly expressed in retinal ganglion and inner cell layers, migrated pretectal, and tectal cells, that is, cells associated with vision. There are two more areas with strong *miR-181a* and *miR-181b* expression, a group of cells located in the central part of the pallium, close to the olfactory bulb and dorsal subpallium, and one in the central medulla oblongata whereas the rest of the larval brain contains weakly expressing cells. Given the adult expression in the olfactory bulb and facial and vagal lobes, the strong telencephalic and medulla oblongata larval expression may correspond to cells that populate these areas. While comparing the regional expression of *miR-181a* and *miR-181b*, significant regional differences are not obvious at larval stages (Table C).

Overall, *miR-181a* and *miR-181b* conserve their regional expression between larval and adult brain (Table H). For example, *miR-181a* and *miR-181b* are expressed in adult pretectal, tectal, hypothalamic and cerebellar cells as it is the case in the larval brain. But there are also differences between larval and adult expression. *miR-181a* and *miR-181b* expression is downregulated in the adult thalamus, periventricular posterior tuberculum and tegmentum. In addition, quantitative differences of expression observed in the larval brain are absent from the adult brain.

Finally, comparison of adult *miR-181a* and *miR-181b* expression reveals similar regional expression but differences at the cellular level within some areas. Good examples are the caudal hypothalamus and the facial lobe. Both *miR*NAs are expressed in these areas but likely in different cells. In other areas, like pretectum and tectum, telencephalon and olfactory bulb, they are likely expressed in the same cell types.

**Additional data file 13. *miR-181a* expression in the zebrafish brain.**

A. transverse section through the larval telencephalon showing *miR-181a* expressing cells throughout the ventral (Sv) and dorsal (Sd) subpallium, pallium (P) and olfactory bulb (OB). The asterisk marks the strongly expressing *miR-181a* cell group in the central pallium, close to OB and Sd.

B. transverse section through the larval diencephalon and optic tectum (TeO) showing strongly *miR-181a* expressing cells in the TeO and migrated pretectal area (M1) and weakly in the preoptic area (Po), ventral thalamus (VT), dorsal thalamus (DT) and eminentia thalami (ET).

C. transverse section through the larval diencephalon and optic tectum (TeO) showing strongly *miR-181a* expressing cells in the TeO and migrated pretectal area (M1) and weakly in the rostral hypothalamus (Hr), periventricular (PT) and migrated (M2) posterior tuberculum, dorsal thalamus (DT) and periventricular pretectum (Pr).

D. transverse section through the larval hindbrain at the level of the posterior lateral line (PLLG) and vagal (VG) ganglia showing *miR-181a* expressing cells in the medulla oblongata (MO). The asterisk marks the strongly *miR-181a* expressing cells in the lateral medulla oblongata likely in proximity to the octaval area (OA?).

E. transverse section through the adult hypothalamus showing *miR-181a* expressing cells in the caudal part of the dorsal zone of the periventricular hypothalamus (Hd) around the ventricular recess (lr) and mammillary body (CM).

F. transverse section through the adult caudal hindbrain showing *miR-181a* expressing cells in the facial (LVII) and glossopharyngeal (LIX) lobes.

G. transverse section through the larval retina showing *miR-181a* expressing cells in the inner part (amacrine cells) of the inner nuclear layer (INL) and the ganglion cell layer (GCL).

H. transverse section through the adult optic tectum showing *miR-181a* expressing cells in the superficial gray zone (sgz), central zone (cz) and the third division of the periventricular gray zone (pgz3).

I. transverse section through the adult dorsal diencephalon and rostral optic tectum showing *miR-181a* expressing cells in the superficial gray zone (sgz), central zone (cz), parvocellular (PSp) and magnocellular (PSm) superficial pretectal nuclei.

**Additional data file 14. *miR-181b* expression in the zebrafish brain.**

A. transverse section through the larval telencephalon showing *miR-181b* expressing cells throughout the ventral (Sv) and dorsal (Sd) subpallium, pallium (P) and olfactory bulb (OB). The asterisk marks the strongly expressing *miR-181b* cell group in the central pallium, close to the OB and Sd.

B. transverse section through the larval telencephalon (caudal to section A) showing *miR-181b* expressing cells throughout the ventral (Sv) and dorsal (Sd) subpallium, pallium (P) and olfactory bulb (OB). The asterisk marks the strongly expressing *miR-181b* cell group in the central pallium, close to the OB and Sd.

C. transverse section through the larval caudal telencephalon at the level of the anterior commissure (ac) showing *miR-181b* expressing cells in the preoptic area (Po), dorsal subpallium (Sd), pallium (P) and habenula (Ha).

D. transverse section through the larval diencephalon and optic tectum (TeO) showing *miR-181b* expressing cells in the TeO, preoptic area (Po), eminentia thalami (ET), ventral (VT) and dorsal (DT) thalamus and migrated pretectal area (M1).

E. transverse section through the larval hypothalamus and midbrain showing strongly expressing *miR-181b* cells in the optic tectum (TeO, periventricular gray zone-pgz and proliferative zone-m).

F. transverse section through the larval hindbrain at the level of the otic capsule (ot) showing *miR-181b* expressing cells in the medulla oblongata (MO). The asterisk marks the strongly *miR-181b* expressing cells in the central medulla oblongata likely in proximity to the octaval area (OA?).

G. transverse section through the adult caudal hindbrain showing *miR-181b* expressing cells in the facial lobe (LVII) and caudal octavolateral nucleus (CON).

H. transverse section through the larval retina showing *miR-181b* expressing cells in the inner part (amacrine cells) of the inner nuclear layer (INL) and the ganglion cell layer (GCL).

I. transverse section through the adult dorsal diencephalon and rostral optic tectum showing *miR-181b* expressing cells in the tectal superficial gray (sgz) and central (cz), zones, parvocellular (PSp), magnocellular (PSm) and central (CPN) superficial pretectal nuclei.

J. transverse section through the adult optic tectum showing *miR-181b* expressing cells in the superficial gray (sgz), central (cz) and periventricular gray (pgz) zones.

K. transverse section through the adult hypothalamus showing *miR-181b* expressing cells (arrowheads) in the caudal part of the dorsal zone of the periventricular hypothalamus around the lateral ventricular recess (Hd-lr) and mamillary body (CM).

**Additional data file 15. *miR-183* expression in the zebrafish brain.**

*miR-183* expression is cell type specific in larvae. It is expressed in retinal photoreceptors and weakly in some inner nuclear layer cells, pineal cells that are again likely to be photoreceptors and perhaps also in parapineal photoreceptors. Outside of the CNS, *miR-183* is expressed in cells that include peripheral sensory neuromasts, olfactory sensory neurons and hair cells of the ear. *miR-182* and *miR-96* show almost identical expression patterns to *miR-183* (Table E) although expression is not as robust.

A. transverse section through the larval rostral brain and retina showing *miR-183* expressing cells in the olfactory epithelium (OE), retinal photoreceptor (Ph) and inner nuclear (INL) layers.

B. transverse section through the larval telencephalon and rostral epithalamus showing *miR-183* expressing cells in the epiphysis (E) and neuromasts (nm).

C. transverse section through the larval epithalamus and pallium (P) showing *miR-183* expressing cells medial to the habenula (Ha) likely corresponding to parapineal organ (arrowhead) and neuromasts (nm).

D. transverse section through the larval midbrain and isthmus showing *miR-183* expressing cells in the trigeminal (TG), anterior lateral line (ALLG) ganglia and neuromasts (nm).

E. transverse section through the larval hindbrain showing *miR-183* expressing cells in the otic capsule (ot), octaval (OG) and glossopharyngeal (GG) ganglia and neuromasts (nm).

F. transverse section through the larval caudal hindbrain showing *miR-183* expressing cells in the posterior lateral line (PLLG) and vagal (VG) ganglia.

G. transverse section through the larval retina showing *miR-183* expressing cells in the retinal photoreceptor (Ph) and inner cellular layer (INL).

**Additional data file 16. *miR-200a* expression in the zebrafish brain.**

*miR-200a* shows cell-type specific expression (Table E). It is expressed in sensory cells of the olfactory epithelium, taste buds, ear, neuromasts as well as the adult primary olfactory fiber layer.

A. transverse section through the rostral part of the larval head showing *miR-200a* expressing cells in the olfactory epithelium (OE) and the taste buds (arrowhead).

B. transverse section through the caudal part of the larval head showing *miR-200a* expressing cells in the taste buds (arrowheads).

C. transverse section through the larval part of the head at the level of epithalamus (Ha, habenula) showing *miR-200a* expressing cells in neuromasts (arrowhead).

D. transverse section through the adult olfactory bulb (OB) showing *miR-200a* expressing cells in the primary olfactory fiber layer (POF). These may be transcripts in axons of olfactory sensory neurons.

**Additional data file 17. *miR-218a* expression in the zebrafish brain.**

*miR-218a* shows cell type specific expression in cranial motor nuclei (III, V, VI, VII, IX, X) and spinal motoneurons (Table E). *miR-218a* expression is conserved in adult motor nuclei, but also expands in rostral brain areas including the ventral telencephalon, magnocellular preoptic area, ventral and lateral hypothalamic nuclei, optic tectum and inferior olive (Table I).

A. transverse section through the larval hindbrain at the level of the octaval ganglion (OG) showing *miR-218a* expression in the trigeminal motor nucleus (NV).

B. transverse section through the larval hindbrain at the level of the otic capsule (ot, caudal to section A) showing *miR-218a* expression in the facial (NVII) and abducens (NVI) motor nuclei.

C. transverse section through the larval hindbrain at the level of the otic capsule (ot, caudal to section B) showing *miR-218a* expression in the facial motor nucleus (NVII).

D. transverse section through the larval hindbrain at the level of the posterior lateral line (PLLG) and vagal (VG) ganglia showing *miR-218a* expression in the glossopharyngeal motor nucleus (NIX).

E. transverse section through the larval caudal hindbrain (caudal to section D) showing *miR-218a* expression in the vagal motor nucleus (NX).

F. transverse section through the larval caudal hindbrain (caudal to section E) showing *miR-218a* expressing cells in the vagal motor nucleus (NX) and motor neurons (MN) of the caudal inferior reticular formation.

G. parasagittal confocal section through the larval hindbrain showing *miR-218a* expressing cells in the facial (NVII) and vagal (NX) motor nuclei.

H. parasagittal confocal section through the larval hindbrain showing cells expressing Tg(isl1:gfp) in the facial (NVII) and vagal (NX) motor nuclei.

I. superimposition of sections G and H showing co-localization (in yellow) of *miR-218a* (red) and GFP (green) expression in the facial (NVII) and vagal (NX) motor nuclei.

J. transverse section through the larval spinal cord showing *miR-218a* expressing cells in motor neurons (MN).

K. transverse section through the adult preoptic area at the level of the optic chiasma (oc) showing *miR-218a* expressing cells in the magnocellular preoptic nucleus (PM).

L. transverse section through the adult ventral hypothalamus showing *miR-218a* expressing cells in the lateral hypothalamic nucleus (LH) and ventral zone of the periventricular hypothalamus (Hv).

M. transverse section through the adult caudal midbrain showing weak *miR-218a* expressing cells in the oculomotor nucleus (NIII).

N. transverse section through the adult hindbrain at the level of the trigeminal nerve (ventral motor root, Vmv) showing *miR-218a* expressing cells in the ventral part of the trigeminal motor nucleus (NVmv).

O. transverse section through the adult hindbrain at the level of the octaval nerve (VIII) showing *miR-218a* expressing cells in the rostral part of the abducens nucleus (NVIr).

P. transverse section through the adult hindbrain at the level of the octaval nerve (VIII, caudal to section O) showing *miR-218a* expressing cells in the caudal part of the abducens (NVIc) and motor facial (NVIIm) nuclei.

Q. transverse section through the adult caudal hindbrain at the level of the vagal nerve (caudal to section P) showing *miR-218a* expressing cells in the vagal motor nucleus (NXm) and inferior olive (IO).

R. transverse section through the adult spinal cord showing *miR-218a* expressing cells in motor neurons (MN) of the ventral horn (VH).

**Additional data file 18. *miR-219* expression in the zebrafish brain**

*miR-219* is expressed in both periventricular and differentiated cells in the larval brain and has more restricted expression in differentiating cells compared to *miR-124*. Transcripts are localized in periventricular and adjacent cells only in few cases. *miR-219* shows pronounced changes between larval and adult brain expression patterns. *miR-219* transcript localization suggests downregulation of expression during maturity and conserved or de novo expression in distinct cells, possibly glia.

In the 5dpf larval brain, *miR-219* is expressed in the entire ventral and dorsal thalamus, tegmentum, isthmus, medulla oblongata and ventral hindbrain areas such as the reticular formation. In addition, it is expressed in most pretectal cells, in a few pallial, ventral habenular, dorsal posterior tubercular, tectal and lateral cerebellar plate cells. It is absent from the caudal preoptic area, hypothalamus, retina and Mauthner cells (Table B). In the adult brain, it is widely expressed only in the caudal hindbrain. In the rostral adult brain, expression is often downregulated as is the case in the ventral thalamus and is limited to few distinct cells found in areas that are positive (pallium, epithalamus, central posterior (dorsal) thalamic nucleus, posterior tuberculum, pretectum, tectum, tegmentum, cerebellum) or negative in the larval brain (olfactory bulb, subpallium, caudal preoptic area, hypothalamus). In some cases these distinct adult cells, possibly glia, are associated with nerves or tracts such as the lateral olfactory tract, optic tract or commissures such as anterior, postoptic and tectal commissure, whereas the equivalent pathways in larvae are devoid of staining (Table G).

A. transverse section through the larval caudal telencephalon and rostral diencephalon showing *miR-219* weakly expressing cells in the rostral preoptic area (Po), eminentia thalami (ET), pallium (P), dorsal thalamus (DT) and habenula (Ha).

B. transverse section through the larval diencephalon and optic tectum showing *miR-219* expressing cells in the ventral (VT) and dorsal (DT) thalamus and pretectum (Pr).

C. transverse section through the larval diencephalon and optic tectum showing *miR-219* expressing cells in the dorsal periventricular posterior tuberculum (PTd), dorsal thalamus (DT) and pretectum (Pr).

D. transverse section through the larval hypothalamus, caudal midbrain and isthmus showing *miR-219* expressing cells in the semicircular torus (TS), isthmic area (Is) and optic tectum (TeO).

E. transverse section through the caudal larval midbrain and isthmus showing *miR-219* expressing cells in the semicircular torus (TS), isthmic area (Is), isthmic nucleus (NI), superior raphe (SR), superior reticular formation (SRF) and lateral cerebellar plate (CeP, arrowhead).

F. transverse section through the larval hindbrain at the level of the octaval ganglion showing *miR-219* expressing cells in the medulla oblongata (MO), presumptive octaval area (OA) and intermediate reticular formation (IMRF). The Mauthner cell (MAC) is devoid of expression.

G. transverse section through the adult telencephalon at the level of the anterior commissure showing *miR-219* expressing cells (arrows) scattered throughout telencephalic areas [Vs-supracommissural nucleus of the ventral (subpallial) telencephalic area, Dm-medial, Dc-central, Dl-lateral, Dp-posterior zones of the dorsal (pallial) telencephalic area, ac-anterior commissure].

H. higher magnification of G showing *miR-219* expressing cells (arrows) scattered throughout telencephalic areas [Dm-medial, Dc-central, Dl-lateral, Dp-posterior zones of the dorsal (pallial) telencephalic area].

I. transverse section through the adult diencephalon at the level of the postoptic commissure (poc) showing *miR-219* expressing cells in the poc and the optic tract (OT), the pretectal central (CPN) and superficial parvocellular (PSp) nuclei and the posterior preoptic parvocellular nucleus (PPp, arrow).

J. transverse section through the adult diencephalon and optic tectum showing *miR-219* expressing cells scattered in the ventral zone of the hypothalamus (Hv), the ventral optic tract (VOT) and the tectal superficial gray zone (sgz).

K. transverse section through the optic tectum (caudal to section J) showing *miR-219* expressing cells in the tectal commissure, periventricular (pgz) and superficial (sgz) gray zones and longitudinal torus (TL).

L. transverse section showing *miR-219* expressing cells (arrows) scattered throughout the cerebellum (CCe) and granular eminence (EG).

M. transverse section through the adult ventral hindbrain showing *miR-219* expressing cells scattered in the anterior octaval area (AON), intermediate reticular formation (IMRF) and in proximity to medial longitudinal fascicle (MLF). The areas dorsal to and the Mauthner cell (MAC) are devoid of expression.

N. transverse section through the adult ventral hindbrain showing *miR-219* expressing cells scattered in the intermediate reticular formation (IMRF), anterior lateral line nerve (ALLN), octaval nerve (VIII) and in proximity to medial longitudinal fascicle (MLF) and rostral part of the abducens nucleus (NVIr). The areas dorsal to IMRF are devoid of expression.

O. transverse section through the adult caudal hindbrain showing *miR-219* expressing cells throughout this area, in particular in the vagal lobe (LX), medial funicular nucleus (MFN), inferior reticular formation (IRF) and in proximity to tracts such as the bulbospinal tract (TBS) and the MLF.

**Additional data file 19. *miR-221* expression in the zebrafish brain.**

*miR-221* shows spatially localized expression. It is strongly expressed in lateral hypothalamic areas (cells around the lateral recess, in the lateral torus and diffuse inferior lobe) and at high levels in caudal pallium and eminentia thalami. It is weakly expressed in a few rostral ventral telencephalic, thalamic, periventricular posterior tubercular, semicircular torus and rostral tectal cells (Table D).  *miR-221* expression is largely conserved in the adult telencephalic, hypothalamic, thalamic, posterior tubercular and semicircular torus nuclei (table I). However, there are some differences between the larval and adult *miR-221* brain expression. *miR-221* is expressed in adult preoptic area cells, isthmic nucleus, facial and vagal lobes, areas devoid of larval expression. *miR-221* is also expressed in two cell populations in the adult tectal periventricular gray zone, area with few weakly stained cells in the larva.

*miR-221* belongs to the same cluster as *miR-222* and seems to largely share expression patterns but they also have subtle differences in transcript localisation. For instance, only *miR-222* is expressed in the larval ventral intermediate hypothalamus, whereas only *miR-221* is expressed in adult thalamic and pretectal nuclei, many adult migrated posterior tuberculum nuclei, larval and adult semicircular torus cells (tables D, I).

A. transverse section through the larval diencephalon showing weakly *miR-221* expressing cells in the eminentia thalami (ET) and thalamic area (arrowhead, dorsal thalamus-DT, ventral thalamus-VT).

B. transverse section through the larval diencephalon and midbrain showing *miR-221* strongly expressing cells at the level of the intermediate hypothalamus (Hi) in the diffuse nucleus of the hypothalamic inferior lobe (DIL) and the lateral torus (TLa) and weakly expressing cells in the optic tectum (arrowheads, TeO).

C. transverse section through the larval hypothalamus and midbrain showing *miR-221* strongly expressing cells at the level of the intermediate hypothalamus (Hi) in the diffuse nucleus of the hypothalamic inferior lobe (DIL).

D. transverse section through the adult rostral telencephalon showing robust expression of *miR-221* in the dorsal telencephalon/pallium (P) and weakly in the internal cellular layer of the olfactory bulb (ICL).

E. transverse section through the adult telencephalon showing *miR-221* expressing cells in the ventral (Vv) and dorsal (Vd) nuclei of the ventral telencephalon (subpallium), medial (Dm), central (Dc), dorsal (Dd) and lateral (Dl) zones of the dorsal telencephalon.

F. transverse section through the adult preoptic area at the level of the optic chiasma (posterior to section E) showing *miR-221* expressing cells in the suprachiasmatic nucleus (SC), preoptic posterior parvocellular (PPp) and magnocellular (PM) nuclei.

G. transverse section through the adult telencephalon at the level of anterior commissure (ac) showing *miR-221* expressing cells in the supracommissural nucleus of the ventral telencephalon (Vs), medial (Dm), dorsal (Dd), lateral (Dl) and posterior (Dp) zones of the dorsal telencephalon.

H. transverse section through the adult diencephalon showing *miR-221* expressing cells in the ventrolateral thalamic nucleus (VL) and the caudal telencephalon (Te).

I. Higher magnification of the section E at the level of the thalamus showing *miR-221* expressing cells in the ventrolateral thalamic nucleus (VL).

J. transverse section through the adult hypothalamus showing *miR-221* expressing cells in the ventral zone of periventricular hypothalamus (Hv), anterior tuberal nucleus (ATN), diffuse nucleus of the hypothalamic inferior lobe (DIL), nucleus of the paraventricular organ (nPVO), nucleus of periventricular posterior tuberculum (TPp) and medial preglomeral nucleus (PGm).

K. transverse section through the adult hypothalamus showing *miR-221* expressing cells in the caudal zone of the periventricular hypothalamus (Hc), dorsal zone of the periventricular hypothalamus (Hd, at the level of lateral ventricular recess-lr) and diffuse nucleus of the hypothalamic inferior lobe (DIL).

L. transverse section through the adult hypothalamus showing *miR-221* expressing cells in the ventral (Hv) and dorsal (Hd) zones of periventricular hypothalamus, anterior tuberal nucleus (ATN), lateral hypothalamic nucleus (LH), and nucleus of periventricular posterior tuberculum (TPp).

M. transverse section through the adult isthmus and caudal midbrain showing *miR-221* strongly expressing cells in the tectal periventricular gray zone (pgz), central nucleus of semicircular torus (TSc, arrowheads), and weakly in the nucleus of lateral valvula (NLV, arrowhead).

N. transverse section through the adult isthmus and caudal midbrain showing *miR-221* strongly expressing cells in the tectal periventricular gray zone (pgz) of the optic tectum (TeO), central nucleus of semicircular torus (TSc), isthmic nucleus (NI) and weakly in the nucleus of lateral valvula (NLV, arrowhead).

O. transverse section through the adult hypothalamus showing *miR-221* expressing cells in the anterior tuberal nucleus (ATN), lateral hypothalamic nucleus (LH) and nucleus of the paraventricular organ (nPVO).

**Additional data file 20. *miR-222* expression in the zebrafish brain.**

*miR-222* expression is restricted to differentiating cells of the forebrain and midbrain and expression is largely conserved in these areas throughout life. In addition, *miR-222* is de novo expressed in the adult facial and vagal lobes. In the larval brain, *miR-222* is expressed in cells of all telencephalic areas apart from the lateral telencephalic area M4. In the diencephalon, *miR-222* is expressed in the preoptic area, ventral thalamus and eminentia thalami, rostral and intermediate hypothalamic nuclei. More caudally, it is expressed in ventral and migrated posterior tubercular cells and a few scattered cells of the tectal periventricular gray zone (Table D). In the adult brain, *miR-222* expression is conserved in the above areas with the exception of the thalamus where it is downregulated. In addition, *miR-222* is expressed in the adult facial and vagal lobes although in the larva we did not observe expression in any hindbrain cells that could correspond to these adult areas (Table I).

A. transverse section through the larval telencephalon showing *miR-222* expressing cells in the ventral (Sv) and dorsal (Sd) subpallium and pallium (P).

B. transverse section through the larval telencephalon (caudal to section A, at the level of the anterior commissure, arrowhead) showing *miR-222* expressing cells in the preoptic area (Po), dorsal subpallium (Sd) and pallium (P).

C. transverse section through the larval caudal telencephalon and rostral diencephalon showing *miR-222* expressing cells in the preoptic area (Po), eminentia thalami (ET), ventral thalamus (VT) and pallium (P).

D. transverse section through the larval hypothalamus and midbrain showing *miR-222* expressing cells in the intermediate hypothalamus (Hi, ventral and at the level of lateral ventricular recess-lr), diffuse nucleus of the hypothalamic inferior lobe (DIL), lateral torus (TLa), ventral periventricular (PTv) and migrated posterior tubercular area (M2).

E. transverse section through the larval hindbrain at the level of the posterior lateral line ganglion (PLLG) where the medulla oblongata (MO) is devoid of *miR-222* expression. The dorsal medulla oblongata at this level may give rise to cells of the facial and vagal lobes.

F. transverse section through the young adult caudal telencephalon at the level of the anterior commissure (ac) showing *miR-222* expressing cells in the anterior parvocellular preoptic nucleus (PPa), endopeduncular nucleus (EN, part of eminentia thalami), supracommissural nucleus of the ventral telencephalon (Vs), medial (Dm) and lateral (Dl) zones of the dorsal telencephalon.

G. transverse section through the adult hypothalamus showing *miR-222* expressing cells in the ventral zone of the periventricular hypothalamus (Hv), anterior tuberal nucleus (ATN), dorsal zone of the periventricular hypothalamus at the level of the lateral hypothalamic ventricular recess (Hd-lr), diffuse nucleus of the inferior hypothalamic lobe (DIL), lateral hypothalamic torus (TLa) and nucleus of the paraventricular organ (nPVO).

H. transverse section through the adult hypothalamus showing *miR-222* expressing cells in the anterior tuberal nucleus (ATN), dorsal zone of the periventricular hypothalamus at the level of the lateral hypothalamic ventricular recess (Hd-lr), diffuse nucleus of the inferior hypothalamic lobe (DIL), lateral hypothalamic torus (TLa), nucleus of the paraventricular organ (nPVO) and posterior thalamic nucleus (Pt).

I. transverse section through the adult caudal hindbrain showing *miR-222* expressing cells superficially lining the facial (LVII) and vagal (LX) lobes.

J. transverse section through the adult rostral telencephalon showing *miR-222* expressing cells in the dorsal telencephalon/pallium (P) and internal cellular layer of olfactory bulb (ICL).

K. transverse section through the adult telencephalon showing *miR-222* expressing cells in the ventral (Vv) and dorsal (Vd) nuclei of the ventral telencephalon (subpallium), medial (Dm), central (Dc), dorsal (Dd), lateral (Dl) and posterior (Dp) zones of the dorsal telencephalon.

L. transverse section through the adult telencephalon at the level of anterior commissure (ac) showing *miR-222* expressing cells in the anterior parvocellular preoptic nucleus (PPa), dorsal part of the entopeduncular nucleus (ENd, part of the eminentia thalami), supracommissural/posterior nucleus of the ventral telencephalon (Vs), medial (Dm), dorsal (Dd) and lateral (Dl) zones of the dorsal telencephalon.

**Additional data file 21. *miR-375* expression in the zebrafish brain.**

*miR-375* shows cell type specific expression the pituitary, a few hypothalamic cells and cranial nerve ganglia (Table E).

A. transverse section through the embryonic intermediate hypothalamus (Hi) showing weak *miR-375* expression in the hypothalamus (arrowheads) and strong expression in the pituitary (Pi).

**Additional data files 22, 23 and 24. *let-7a, let-7b* and *let-7c* expression in the zebrafish brain.**

*let-7a, let-7b* and *let-7c* are expressed in both proliferating and differentiating cells. *let-7b* and *let-7c* differ in their sequence in only one nucleotide located outside the seed region. They share similar regional expression in the larval brain with two differences: *let-7b* is expressed in the retinal ciliary marginal zone and pineal cells whereas *let-7a* and *let-7c* are absent (table A). *let-7a, let-7b* and *let-7c* mainly conserve their regional expression between larval and adult brain (tables A, F).

*let-7a, let-7b* and *let-7c* are expressed in many proliferating and differentiating cells of the larval fore-, mid- and hindbrain with the exception of some areas such as hypothalamic nuclei (caudal hypothalamus, diffuse nucleus of inferior lobe, lateral torus) interpeduncular nucleus, locus coereleus, raphe and reticular formation. We detected only minor differences at the regional level between larval and adult brain expression. For example, *let-7b* and *let-7c* are expressed in some adult but not larval hypothalamic lateral torus and superior raphe cells (tables A, F).

**Additional data file 22. *let-7a* expression in the zebrafish brain.**

A. transverse section through the larval telencephalon showing *let-7a* expressing cells in the ventral (Sv) and dorsal (Sd) subpallium and pallium (P).

B. transverse section through the larval telencephalon and epithalamus showing *let-7a* expressing cells in the ventral (Sv) and dorsal (Sd) subpallium, pallium (P), migrated telencephalic area (M4) and habenula (Ha). Pineal cells (E) are devoid of expression.

C. transverse section through the larval diencephalon and rostral optic tectum showing *let-7a* expressing cells in the preoptic area (Po), eminentia thalami (ET), ventral thalamus (VT) and dorsal (DT) thalamus, periventricular (Pr) and migrated (M1) pretectum and optic tectum (TeO, including the tectal proliferative zone, m).

D. transverse section through the larval retina (dorsal to the right) devoid of *let-7a* expressing cells. The arrow points at the ciliary marginal zone.

E. transverse section through the larval diencephalon and midbrain showing mainly periventricular (arrowheads) *let-7a* expressing cells in the rostral hypothalamus (Hr), periventricular (PT) and migrated (M2) posterior tuberculum, dorsal thalamus (DT), tegmentum (T) and tectal periventricular gray zone (pgz).

F. transverse section through the larval hypothalamus, midbrain and rostral hindbrain showing *let-7a* expressing cells in the intermediate hypothalamus (Hi, area of the periventricular hypothalamic recess-lr), semicircular torus (TS), tegmentum (T), tectal periventricular gray zone (pgz) and cerebellar valvula (Va).

G. transverse section through the larval hypothalamus, midbrain and rostral hindbrain showing *let-7a* expressing cells in the intermediate (Hi) and caudal (Hc) hypothalamus, semicircular torus (TS), tegmentum/isthmic area (T/Is), periventricular gray zone (pgz) of the optic tectum (TeO), cerebellar valvula (Va) and cerebellar plate (CeP).

H. transverse section through the larval hindbrain at the level of the otic capsule (ot) showing *let-7a* expressing cells in the medulla oblongata (MO).

**Additional data file 23. *let-7b* expression in the zebrafish brain.**

A. transverse section through the larval rostral telencephalon showing *let-7b* expressing cells in the olfactory bulb (OB).

B. transverse section through the larval telencephalon showing *let-7b* expressing cells in the ventral (Sv) and dorsal (Sd) subpallium and pallium (P).

C. transverse section through the larval rostral diencephalon showing *let-7b* expressing cells in the preoptic area (Po), eminentia thalami (ET), migrated eminentia thalami (M3), pallium (P), habenula (Ha) and epiphysis (E).

D. transverse section through the larval diencephalon and rostral optic tectum showing *let-7b* expressing cells in the preoptic area (Po), eminentia thalami (ET), migrated eminentia thalami (M3), ventral thalamus (VT), dorsal thalamus (DT), and optic tectum (TeO).

E. transverse section through the larval retina (dorsal to the right) showing *let-7b* expressing cells in the ciliary marginal zone (CMZ).

F. transverse section through the larval diencephalon and midbrain showing mainly periventricular (arrowheads) *let-7b* expressing cells in the dorsal (PTd) and ventral (PTv) periventricular and lateral migrated (M2) posterior tuberculum, tegmentum (T), semicircular torus (TS), longitudinal torus (TL), tectal proliferative (m) and periventricular gray (pgz) zones.

G. transverse section through the larval caudal midbrain and rostral hindbrain showing *let-7b* expressing cells in the semicircular torus (TS), optic tectum (TeO), isthmic/medulla oblongata area (Is/MO) and cerebellar plate (CeP).

H. transverse section through the larval rostral hindbrain showing *let-7b* expressing cells in the semicircular torus (TS), cerebellar plate (CeP) and isthmic/medulla oblongata area (Is/MO).

I. transverse section through the larval hindbrain at the level of the otic capsule showing *let-7b* expressing cells in the medulla oblongata (MO) and rhombic lip (RL).

J. transverse section through the adult rostral telencephalon showing *let-7b* expressing cells in the dorsal telencephalon/pallium (P) and olfactory bulb (OB).

K. transverse section through the adult caudal hindbrain showing *let-7b* expressing

cells in the inferior reticular formation (IRF), posterior octaval (PON) and caudal octavolateral (CON) areas, facial lobe (LVII) and around the rhomboncephalic ventricle (arrowhead).

**Additional data file 24. *let-7c* expression in the zebrafish brain.**

A. transverse section through the larval telencephalon showing *let-7c* expressing cells in the ventral (Sv) and dorsal (Sd) subpallium and pallium (P).

B. transverse section through the larval caudal telencephalon and epithalamus showing *let-7c* expressing cells in the dorsal subpallium (Sd), pallium (P) and habenula (Ha).

C. transverse section through the larval diencephalon and optic tectum showing *let-7c* expressing cells in the ventral thalamus (VT), dorsal thalamus (DT), periventricular (Pr) and migrated (M1) pretectum and optic tectum (TeO, proliferative zone-m, periventricular gray zone-pgz, longitudinal torus-TL).

D. transverse section through the larval diencephalon and midbrain showing mainly periventricular (arrowheads) *let-7c* expressing cells in the periventricular (PT) and lateral migrated (M2) posterior tuberculum, tegmentum (T), semicircular torus (TS) and tectal periventricular gray zone (pgz).

E. oblique transverse section through the larval caudal hypothalamus, midbrain and hindbrain showing *let-7c* expressing cells in the semicircular torus (TS), optic tectum (TeO), isthmic area (Is), cerebellar plate (CeP), and medulla oblongata (MO).

F. transverse section through the larval retina (dorsal to the right) devoid of *let-7c* expressing cells. The arrow points at the ciliary marginal zone.

G. oblique transverse section through the larval hindbrain at the level of the otic capsule (caudal to section E) showing *let-7c* expressing cells in the semicircular torus (TS), optic tectum (TeO), granular cerebellar eminence (EG), cerebellar plate (CeP) and medulla oblongata (MO).

H. oblique transverse section through the larval hindbrain at the level of the octaval ganglion (caudal to section G) showing *let-7c* expressing cells in the granular cerebellar eminence (EG), medulla oblongata (MO) and rhombic lip (RL).

I. transverse section through the adult rostral telencephalon showing *let-7c* expressing cells in the dorsal telencephalon/pallium (P) and olfactory bulb (OB).

J. transverse section through the adult caudal hindbrain showing *let-7c* expressing

cells in the inferior reticular formation (IRF), posterior octaval (PON) and caudal octavolateral (CON) nuclei and facial lobe (LVII).

**Additional data file 25. Other miRNAs expressed in the zebrafish brain.**

In addition to the 21 described above, we have examined the expression of seventeen additional *miR*NAs in the zebrafish brain. In this figure and following tables (Additional file AF27) we show examples of their expression.

A. transverse section through the larval rostral telencephalon showing *miR-139* expressing cells in the olfactory epithelium (OE). *miR-139* is widely expressed in differentiated cells in the zebrafish brain (see also panel D).

B. transverse section through the adult olfactory bulb showing *miR-187* expressing cells in glomerular (GL) and internal cellular (ICL) layers. *miR-187* is widely expressed in differentiated cells in the adult zebrafish brain.

C. transverse section through the embryonic rostral diencephalon showing *miR-454a* expressing cells in the preoptic area (Po), eminentia thalami (ET), migrated eminentia thalami (M3), habenula (Ha), and retina. The arrow points at the ciliary marginal zone. *miR-454a* is almost ubiquitously expressed in the larval zebrafish brain.

D. transverse section through the adult hypothalamus showing *miR-139* expressing cells in ventral (Hv) and dorsal (Hd) zones of periventricular hypothalamus, anterior tuberal nucleus (ATN), lateral hypothalamic nucleus (LH), posterior tuberal nucleus (PTN), lateral torus (TLa) and lateral preglomerular nucleus (PGl). *miR-139* is widely expressed in differentiated cells in the zebrafish brain.

E. transverse section through the larval retina showing *miR-132* expressing cells in the ciliary marginal zone (CMZ, arrow), inner nuclear layer (INL) and ganglion (GCL) cell layers. *miR-132* is widely expressed in the zebrafish brain.

F. transverse section through the larval retina showing *miR-125b* expressing cells in the ciliary marginal zone (CMZ, arrow), inner (INL) and ganglion (GCL) cell layers. *miR-125b* is almost ubiquitously expressed in the zebrafish brain.

G. transverse section through the adult hypothalamus showing *miR-132* expressing cells in dorsal zone of periventricular hypothalamus (Hd), anterior tuberal nucleus (ATN), lateral hypothalamic nucleus (LH), posterior tuberal nucleus (PTN), medial preglomerular nucleus (PGm) and posterior thalamic nucleus (Pt). *miR-132* is widely expressed in differentiated cells in the zebrafish brain (see also panel E).

H. transverse section through the adult preoptic area showing *miR-98* expressing cells in the suprachiasmatic nucleus (SC) and parvocellular posterior preoptic nucleus (PPp). *miR-98* is widely expressed in the adult zebrafish brain.

I. transverse section through the adult preoptic area showing *miR-34b* expressing cells lining and lateral to the ventricle in the anterior parvocellular preoptic nucleus (PPa). *miR-34b* is mainly expressed in periventricular cells of the adult zebrafish brain (see also panel L.

J. transverse section through the larval diencephalon and optic tectum (TeO) showing *miR-125a* expressing cells in the ventral (VT) and dorsal (DT) thalamus and the periventricular gray zone (pgz) of the TeO. *miR-125a* is widely expressed in differentiated cells of the zebrafish brain.

K. transverse section through the adult dorsal diencephalon showing *miR-100* expressing cells in the habenular nuclei (dorsal-Had, ventral-Hav), ventromedial thalamic nucleus (VM) and optic tectum (TeO). *miR-100* is widely expressed in the zebrafish brain.

L. transverse section through the adult thalamic area showing *miR-34b* expressing cells lining the ventricle in the ventromedial thalamic nucleus (VM). *miR-34b* is mainly expressed in periventricular cells of the adult zebrafish brain (see also panel I).

M. transverse section through the larval midbrain showing *miR-99* expressing cells in the periventricular gray zone (pgz) of the optic tectum (TeO), tegmentum (T), semicircular torus (TS) and intermediate hypothalamus (Hi). *miR-99* is widely expressed in periventricular and differentiating cells of the larval zebrafish brain.

N. transverse section through the adult isthmus showing *miR-103* expressing cells in the central gray (GC), superior raphe (SR), isthmic nucleus (NI), nucleus of lateral valvula (NLV) and diffuse nucleus of inferior hypothalamic lobe (DIL). *miR-103* is widely expressed in the zebrafish brain.

O. transverse section through the adult cerebellum showing *miR-16* expressing cells in the cerebellar granular layer (CeGL). *miR-16* is widely expressed in the zebrafish brain.

**Additional data file 29. Mismatch test for *let-7a*, *miR-92b, miR-153a* and *miR-181a*.**

To test the ability of LNA probes to discriminate between different members within a miRNA family, we performed in situ hybridization with single and double mismatch probes for *let-7a*, *miR-92b*, *miR-153a* and *miR-181a*. In parallel we performed in situ hybridization with the fully matching LNA probes. All probes were labeled and hybridized in parallel.

A,C,E,G: show in situ hybridization signal for full matching LNA probes *miR-181a*, *let-7a*, *miR-92b* and *miR-153a*, respectively.

B,D,F,H: show in situ hybridization signal for a double mismatch *miR-181a* probe (MM*miR-181a*) and single mismatch probes *let-7a*, *miR-92b* and *miR-153a* (MM*let-7a*, MM*miR-92b* and MM*miR-153a*), respectively.

Although there is a strong reduction in hybridization signal with the single mismatch probes (MM*let-7a*, MM*miR-92b* and MM*miR-153a*), hybridization was only fully eliminated by the double mismatch probe MM*miR-181a*. Thus, LNA probes may not be able to fully discriminate between miRNA family members that differ at a single position.

**Abbreviations**.

I Olfactory nerve

II Optic nerve

III Oculomotor nerve

IV Trochlear nerve

V Trigeminal nerve

Vmd Dorsal motor root of the trigeminal nerve

Vs Sensory root of the trigeminal nerve

VI Abducens nerve

VIc Caudal root of the abdcens nerve

VIr Rostral root of the abducens nerve

VII Facial nerve

VIIs Sensory root of the facial nerve

VIII Octaval nerve

IX Glossopharyngeal nerve

X Vagal nerve

A Anterior thalamic nucleus

ac Anterior commisure

ALLG Anterior lateral line ganglion

ALLN Anterior lateral line nerves

AON Anterior octaval nucleus

AP Area postrema

APN Accessory pretectal nucleus

ATN Anterior tuberal nucleus

CC Cerebellar crest

CCe Cerebellum

CeGaL Cerebellar ganglionic layer

CeGL Cerebellar granular layer

CeML Cerebellar molecular layer

CeP Cerebellar plate

Cer Cerebellar commissure

Cgus Commissure of the secondary gustatory tract

Chor Horizontal commissure

Cinf Commissure infima of Haller

CIL Central nucleus of the inferior lobe

CM Mammilary body

CMZ Ciliary marginal zone

CON Caudal octavolateral nucleus

CP Central posterior thalamic nucleus

CPN Central pretectal nucleus

Cpost Posterior commissure

Ctec Tectal commissure

Cven Ventral rhomboncephalic commissure

cz Central zone

D Dorsal telencephalic area /Pallium

DAO Dorsal accessory optic nucleus

Dc Central zone of dorsal telencephalic area

Dd Dorsal zone of dorsal telencephalic area

DH Dorsal horn

DIL Diffuse nucleus of the inferior lobe

Dl Lateral zone of dorsal telencephalic area

Dm Medial zone of dorsal telencephalic area

DON Descending octaval nucleus

DOT dorsomedial optic tract

DP Dorsal posterior thalamic nucleus

Dp Posterior zone of dorsal telencephalic area

DTN Midbrain dorsal tegmental nucleus

DT Dorsal thalamus

DV Descending trigeminal root

dwz Deep white zone

E Epiphysis/ Pineal

ECL External cellular layer (including mitral cells)

EG Granular eminence

ENd Endopeduncular nucleus, dorsal part

ENv Endopeduncular nucleus, ventral part

ET Eminentia thalami

FG Facial Ganglion

FR Habenulointerpeduncular tract

GC Central grey

GCL Ganglion cell layer

GG Glossopharyngeal ganglion

GL Glomerular layer

H Hypothalamus

Ha Habenula

Had Dorsal Habenular nucleus

Hav Ventral Habenular nucleus

Hc Caudal (zone of periventricular) hypothalamus

Hd Dorsal zone of periventricular hypothalamus

Hd-lr Area of hypothalamic lateral ventricular recess

Hi Intermediate hypothalamus

Hr Rostral hypothalamus

Hv Ventral zone of periventricular hypothalamus

I Intermediate thalamic nucleus

IAF Inner arcuate fibers

ICL Internal cellular layer of olfactory bulb

IMR Intermediate raphe

IMRF Intermediate reticular formation

INL Inner nuclear layer

IO Inferior olive

IR Inferior raphe

IRF Inferior reticular formation

Is Isthmic area including isthmic nucleus and nucleus lateralis valvula

LVII Facial lobe

LIX Glossopharyngeal lobe

LX Vagal lobe

LC Locus coeruleus

LCa Caudal lobe of cerebellum

LFB Lateral forebrain bundle

LH Lateral hypothalamic nucleus

LLF Lateral lemniscus=lateral longitudinal fascicle

LOT Lateral olfactory tract

lr area of hypothalamic lateral ventricular recess

LRN Lateral reticular nucleus

m Tectal Proliferative zone

M1 Migrated pretectal area

M2 Migrated posterior tubercular area

M3 Migrated area of ET

M4 Migrated telencephalic area

MA Mauthner axon

MAC Mauthner cell

MaON Magnocellular octaval nucleus

MFB Medial forebrain bundle

MFN Medial funicular nucleus

MLF Medial longitudinal tract

MNV Mesencephalic nucleus of trigeminal nerve

MO Medulla oblongata

MON Medial octavolateral nucleus

MOT Medial olfactory tract

N Region of the nucleus of medial longitudinal fascicle

NIII Oculomotor nucleus

NIV Trochlear nucleus

NV(md) Trigeminal motor nucleus, dorsal part

Nv(mv) Trigeminal motor nucleus, ventral part

NVs Primary sensory trigeminal nucleus

NVI Abducens motor nuclei

NVII(m) Facial motor nucleus

NIX(m) Glossopharyngeal motor nucleus

NX(m) Vagal motor nucleus

NC Commissular nucleus of Cajal

nc notochord

NDV Nucleus of the descending trigeminal root

NI Isthmic nucleus

NIn Interpeduncular nucleus

NLL Nucleus of the lateral lemniscus

NLV Nucleus of lateral valvula

nMLF Nucleus of the medial longitudinal fascicle

nPVO Nucleus of Paraventricular organ

NT Nucleus taeniae

OA Octaval area

OB Olfactory bulb

oc Optic chiasma

OE Olfactory epithelium

OG Octaval ganglion

OT Optic tract

P Dorsal telencephalic area /Pallium

PGa Anterior preglomerular nucleus

PGc Caudal preglomerular nucleus

PGl Lateral preglomerular nucleus

PGm Medial preglomerular nucleus

pgz (layers 1,2,3) Periventricular gray zone of optic tectum

Ph Photoreceptor layer

Pgz Tectal Periventricular zone

Pi Pituitary

PL Perilemniscal nucleus

PLLG Posterior lateral line ganglion

PLLN Posterior lateral line nerve

PM Magnocellular preopic nucleus

Po Preoptic region

poc Postoptic commissure

POF Primary olfactory fiber layer

PON Posterior octaval nucleus

PPa Parvocellular preoptic nucleus, anterior part

PPd Periventricular pretectal nucleus, dorsal part

PPp Parvocellular preoptic nucleus, posterior part

PPv Periventricular pretectal nucleus, ventral part

Pr Periventricular pretectum

PSm Magnocellular superficial pretectal nucleus

PSp Parvocellular superficial pretectal nucleus

PT Posterior tuberculum

Pt Posterior thalamic nucleus

PTd Dorsal part of periventricular posterior tuberculum

PTN Posterior tuberal nucleus

PTv Ventral part of periventricular posterior tuberculum

RL Rhombic lip

RT Rostral tegmental nucleus

rv Rhombencephalic ventricle

SC Suprachiasmatic nucleus

Sco Subcommissural organ

Sd Dorsal division of ventral telencephalic area

SG Subglomerular nucleus

SGN Secondary gustatory nucleus

sgz Superficial grey zone

SO Secondary octaval population

SR Superior raphe

SRF Superior reticular formation

Sv Ventral division of venral telencephalic area

swz Superficial white zone

T Tegmentum

TBS Bulbospinal tract

Te Telencephalon

TeO Optic tectum

TG Trigeminal gangion

TGN Tertiary gustatory nucleus

TL Longitudinal torus

TLa Lateral torus

TPp Periventricular nucleus of posterior tuberculum

TS Semicircular torus

TSc Central nucleus of semicircular torus

Tsvl Ventrolateral nucleus of semicircular torus

TTB Tectobulbar tract

tv Telencephalic ventricle

V/S Ventral telencephalic area /Subpallium

Va Cerebellar valvula

Val Lateral division of valvula cerebelli

Vam Medial division of valvual cerebelli

VAO Ventral accessory optic nucleus

Vc Central nucleus of ventral telencephalic area

Vd Dorsal nucleus of ventral telencephalic area

VG Vagal ganglion

VH Ventral horn

Vl Lateral nucleus of ventral telencephalic area

VM Ventromedial thalamic nucleus

Vmn Ventral motor root of the trigeminal nerve

VL Ventrolateral thalamic nucleus

VOT ventrolateral Optic tract

Vp Postcommissural nucleus of ventral telencephalic area

Vs Supracommissural nucleus of ventral telencephalic area

VT Ventral thalamus

Vv Ventral nucleus of venral telencephalic area

ZLI Zona limitans intrathalamica
