# Supplementary material for: MicroRNAs show a wide diversity of expression profiles in the developing and mature central nervous system
Source: Genome Biol. 2007 Aug 21;8(8):R173. doi: 10.1186/gb-2007-8-8-r173 (PMC2375003; doi:10.1186/gb-2007-8-8-r173)
Supplement: Additional data file 27 — Tables A-I providing detailed information about the expression of each miRNA in different structures of the zebrafish central and/or peripheral nervous system. [file gb-2007-8-8-r173-S27.doc]

**TABLE A. *miR-92b, miR-9, let-7a, let-7b, let-7c, mir-99* and *mir-100* expression in the larval zebrafish brain.**

In all tables, plus (+) signifies expression, minus (-) signifies absence of expression and empty space signifies that there are no data available.

| **miRNA** | ***miR-92b***  **5dpf** | ***miR-9***  **3dpf** | ***miR-9***  **5dpf** | ***let-7a***  **3dpf** | ***let-7b***  **5dpf** | ***let-7c***  **5dpf** | ***mir-99***  **5dpf** | ***mir-100***  **5dpf** |
| --- | --- | --- | --- | --- | --- | --- | --- | --- |
| Periventricular cells | + | + | + | + | + | + | + | + |
| Proliferative zones | + | + | + | + | + | + | + | + |
| Differentiating cells | - | + | + | + | + | + | + | + |
| Structure-specific | - | - | - | - | - | - | - | - |
| Conserved pattern between embryo and adult | Cell type conservation | Cell type conservation | Cell type conservation | Cell type conservation | Cell type conservation | Cell type conservation |  |  |

| **miRNA** | ***miR-92b***  **5dpf** | ***miR-9***  **3dpf** | ***miR-9***  **5dpf** | ***let-7a***  **3dpf** | ***let-7b***  **5dpf** | ***let-7c***  **5dpf** | ***mir-99***  **5dpf** | ***mir-100***  **5dpf** |
| --- | --- | --- | --- | --- | --- | --- | --- | --- |
| Olfactory epithelium | few | few | few | few | few | - | few | - |
| TELENCEPHALON | Periventricular and adjacent cells | Periventricular and differentiating cells | Periventricular and differentiating cells | Periventricular and differentiating cells | Periventricular and differentiating cells | Periventricular and differentiating cells | Periventricular and differentiating cells | Periventricular and differentiating cells |
| Olfactory bulb- OB | few | + | + | few | + | + | few | few |
| **Ventral telencephalic area /Subpallium -S** | + | + except rostro-ventral part | + except rostro-ventral part | + | + | + | + | + |
| Dorsal division of ventral telencephalic area-**Sd** | + | + | + | + | + | + | + | + |
| Ventral division of venral telencephalic area-**Sv** | + | + | + | + | + | + | + | + |
| **Dorsal telencephalic area /Pallium -P** | + | + | + | + | + | + | + | + |
| Migrated telencephalic area-**M4** | - | + | + | + | + | + | - | - |

| **miRNA** | ***miR-92b***  **5dpf** | ***miR-9***  **3dpf** | ***miR-9***  **5dpf** | ***let-7a***  **3dpf** | ***let-7b***  **5dpf** | ***let-7c***  **5dpf** | ***mir-99***  **5dpf** | ***mir-100***  **5dpf** |
| --- | --- | --- | --- | --- | --- | --- | --- | --- |
| DIENCEPHALON | + | + | + | + | + | + | + | + |
| Preoptic region-Po | + | Periventricular and adjacent cells | Periventricular and adjacent cells | + | + | + | + | + |
| Subcommissural organ | - | - | - | + | + | + | + | + |
| Epithalamus | + | - | - | + | + | + | + | + |
| Pineal/epiphysis-**E** | Few ventral periventricular cells | - | - | - | + | - | + | + |
| Habenula-**Ha** | Periventricular and adjacent cells(ventral/medial) | - | - | + | + | + | + | + |
| **Thalamus** | Periventricular and adjacent cells | Periventricular and differentiating cells | Periventricular and differentiating cells | Periventricular and adjacent cells | Periventricular and adjacent cells | Periventricular and adjacent cells | Periventricular and adjacent cells | Periventricular and adjacent cells |
| Ventral thalamus -**VT** | + | + | + | + | + | + | + | + |
| Dorsal thalamus -**DT** | + | + | + | + | + | + | + | + |
| Eminentia thalami-**ET** | + | + | + | + | + | + | + | + |
| Migrated area of ET-**M3** | - | + | + | - | few | few | - | - |
| Zona limitans intrathalamica-**ZLI** | - | + | + | + | + | + | + |  |
| Hypothalamus-H | Periventricular and adjacent cells | Periventricular and adjacent cells | Periventricular and adjacent cells | Few weak | Few weak | Few weak | Periventricular and adjacent cells | Periventricular and adjacent cells |
| Caudal hypothalamus-**Hc** | + | + | + | - | - | - | + | few |
| Diffuse nucleus of the inferior lobe-**DIL** | + | Few | Few | - | - | - | few | + |
| Area of hypothalamic lateral ventricular recess -**lr** | + | + | + | + | Few | Few | + | + |
| Intermediate hypothalamus –**Hi** | + | + | + | + | + | + | + | + |
| Lateral torus-**TLa** | - | - | - | - | - | - | - | - |
| Rostral hypothalamus-**Hr** | + | + | + | + | + | + | + | + |
| **Pituitary** | - | - | - | - | - | - | - | - |

| **miRNA** | ***miR-92b***  **5dpf** | ***miR-9***  **3dpf** | ***miR-9***  **5dpf** | ***let-7a***  **3dpf** | ***let-7b***  **5dpf** | ***let-7c***  **5dpf** | ***mir-99***  **5dpf** | ***mir-100***  **5dpf** |
| --- | --- | --- | --- | --- | --- | --- | --- | --- |
| Posterior tuberculum-PT | Periventricular cells | + | + | + | + | + | + | + |
| Dorsal part of periventricular posterior tuberculum-**PTd** | + | Periventricular and adjacent cells | Periventricular and adjacent cells | Periventricular and adjacent cells | Periventricular and adjacent cells | Periventricular and adjacent cells | Periventricular and adjacent cells | Periventricular and adjacent cells |
| Migrated posterior tubercular area-**M2** | - | + | + | + | + | + | + | - |
| Ventral part of periventricular posterior tuberculum-**PTv** | + | Periventricular and adjacent cells | Periventricular and adjacent cells | Periventricular cells and adjacent | Periventricular and adjacent cells | Periventricular cells and adjacent | Periventricular cells and adjacent | Periventricular cells and adjacent |
| **Retina** | + | + | + | - | + | - | - | - |
| Ciliary marginal zone-**CMZ** | + | + | + | - | + | - | - | - |
| Ganglion cell layer-**GCL** | - | - | - | - | - | - | - | - |
| Inner nuclear layer-**INL** | - | + | + | - | - | - | - | - |
| Photoreceptor layer-**Ph** | - | - | - | - | - | - | - | - |
| Pretectum | + | + | + | + | + | + | + | + |
| Periventricular pretectum-Pr | + | + | + | + | + | + | + | + |
| Migrated pretectal area-**M1** | - | + | + | + | + | + | + | few |
| **MIDBRAIN** | + | + | + | + | + | + | + | + |
| **Optic tectum-TeO** | + | + | + | + | + | + | few | few |
| Central zone-**cz** | - |  | + | - | - | - | - | - |
| Periventricular gray zone -**pgz** | + | + | + | + | + | + | Few rostral | - |
| Proliferative zone-**m** | + | + | + | + | + | + |  | - |
| Longitudinal torus-**TL** (5d) | - |  | + | + | + | + | + | + |
| Tegmentum-T | Periventricular and adjacent cells | Periventricular and differentiating cells | Periventricular and differentiating cells | Periventricular and adjacent cells | Periventricular and adjacent cells | Periventricular and adjacent cells | Periventricular and adjacent cells | Periventricular and adjacent cells |
| Region of the nucleus of medial longitudinal fascicle-**N** | + | + | + | + | + | + | + | + |
| Dorsal tegmental nucleus-**DTN** (5d) | - |  | + |  |  |  |  |  |
| Occulomotor nerve nucleus-**NIII** | - | + | + |  |  |  |  |  |
| Semicircular torus-**TS** | + | + | + | + | + | + | + | + |
| Interpeduncular nucleus-**NIn** | - | + | + | - | - | - | - | - |

| **miRNA** | ***miR-92b***  **5dpf** | ***miR-9***  **3dpf** | ***miR-9***  **5dpf** | ***let-7a***  **3dpf** | ***let-7b***  **5dpf** | ***let-7c***  **5dpf** | ***mir-99***  **5dpf** | ***mir-100***  **5dpf** |
| --- | --- | --- | --- | --- | --- | --- | --- | --- |
| **HINDBRAIN** | + | + | + | + | + | + | + | + |
| **ISTHMUS** | Periventricular and adjacent cells | Periventricular  and dorsoventral rows of differentiating  cells | Periventricular  and dorsoventral rows of differentiating  cells | Periventricular and dorsoventral rows of differentiating  cells | Periventricular and dorsoventral rows of differentiating  cells | Periventricular  and dorsoventral  rows of  differentiating cells | Periventricular and dorsoventral rows of differentiating  cells | Periventricular and dorsoventral rows of differentiating  cells |
| Isthmic area including isthmic nucleus (**NI)** and nucleus of lateral valvula-**Is** | Periventricular and adjacent cells | + | + | + | + | + | + | + |
| Locus coereleus-**LC** | - |  |  | - | - | - | - | - |
| Superior raphe-**SR** | - | - | - | - | - | - | - | - |
| Superior reticular formation-**SRF** | - | - | Few | - | - | - | - | - |
| Cerebellum | + | + | + | + | + | + | + | + |
| Cerebellar plate-CeP | + | + | + | + | + | + | + | + |
| Granular eminence-**EG** | + | + | + | + | + | + | few | few |
| Cerebellar valvula-**Va** | + | few | few | + | + | + | + | + |
| Medulla oblongata-MO | Periventricular and adjacent cells | Periventricular  and  differentiating  cells  (mediolateral/ dorsoventral  rows) | Periventricular  and  differentiating  cells  (mediolateral/  dorsoventral  rows) | Periventricular and differentiating cells (mediolateral/ dorsoventral rows) | Periventricular and differentiating cells (mediolateral/ dorsoventral rows) | Periventricular  and  differentiating  cells  (mediolateral/ dorsoventral  rows) | Periventricular  and  differentiating  cells | Periventricular  and  differentiating  cells |
| ***Lateral part*** (presumptive Octaval area-**OA?)** | + | + | + | + | + | + | + | + |
| Dorsal part | + | + | + | + | + | + | + | + |
| Rhombic lip-RL | + | + | + | + | + | + | + | + |
| Area postrema-**AP?** (5d) |  |  |  |  |  |  |  |  |
| Facial lobe-LVII? |  |  |  |  |  |  |  |  |
| Glossopharyngeal lobe-LIX? |  |  |  |  |  |  |  |  |
| Vagal lobe-LX? |  |  |  |  |  |  |  |  |
| Cranial nerve nuclei | - |  | (+) |  |  |  |  |  |
| Abducens nucleus-NVI | - |  |  |  |  |  |  |  |
| Facial motor nucleus-**NVII** | - | (-) | (+) |  |  |  |  |  |
| Glossopharyngeal motor nucleus-**NIX** | - |  |  |  |  |  |  |  |
| Trigeminal motor nucleus-**NV** | - | (-) |  |  |  |  |  |  |
| Vagal motor nucleus-**NX** | - |  | (+) |  |  |  |  |  |
| ***Ventral hindbrain areas*** | - | - | + | - | - | - | - | - |
| Inferior olive-**IO** | - | - | - | - | - | - | - | - |
| Inferior raphe-**IR** | - | - | - | - | - | - | - | - |
| Inferior reticular formation-**IRF** | - | - | + | - | - | - | - | - |
| Intermediate raphe-**IMR** | - | - | - | - | - | - | - | - |
| Intermediate reticular formation-**IMRF** | - | - | + | - | - | - | - | - |
| Mauthner cell-**MAC** | - | - | - | - | - | - | - | - |

| **miRNA** | ***miR-92b***  **5dpf** | ***miR-9***  **3dpf** | ***miR-9***  **5dpf** | ***let-7a***  **3dpf** | ***let-7b***  **5dpf** | ***let-7c***  **5dpf** | ***mir-99***  **5dpf** | ***mir-100***  **5dpf** |
| --- | --- | --- | --- | --- | --- | --- | --- | --- |
| **Cranial Ganglia** | + | - | - | - | - | - | - | - |
| Anterior lateral line ganglion-**ALLG** | + | - | - | - | - | - | - | - |
| Facial ganglion-**FG** | + | - | - | - | - | - | - | - |
| Glossopharyngeal ganglion-**GG** | + |  | - | - | - | - | - | - |
| Octaval ganglion-**OG** | + | - | - | - | - | - | - | Few weak |
| Posterior lateral line ganglion-**PLLG** | + | - | - | - | - | - | - | - |
| Trigeminal gangion-**TG** | + | - | - | - | - | - | - | - |
| Vagal ganglion-**VG** | + | - | - | - | - | - | - | - |

**Table B.  *miR-124, miR-138, miR-219* and *miR-135c* expression in the larval zebrafish brain.**

| miRNA | ***miR-124***  **3dpf** | ***miR-124***  **5dpf** | ***miR-138***  **5dpf** | ***miR-219***  **3dpf** | ***miR-219***  **5dpf** | ***miR-135c***  **5dpf** |
| --- | --- | --- | --- | --- | --- | --- |
| Periventricular cells | - | - | - | Few cases | Few cases | + |
| Proliferative zones | - | - | - | - | - | + |
| Differentiating cells | + | + | + | + | + | + |
| Structure-specific | no | no | no | no | no | no |
| Pattern conserved between embryo and adult | Cell type conservation | Cell type conservation | Mixed profile | no | no | Mixed profile |

| **miRNA** | ***miR-124***  **3dpf** | ***miR-124***  **5dpf** | ***miR-138***  **5dpf** | ***miR-219***  **3dpf** | ***miR-219***  **5dpf** | ***miR-135c***  **5dpf** |
| --- | --- | --- | --- | --- | --- | --- |
| Olfactory epithelium | - | - | - | - | - | - |
| TELENCEPHALON | + | + | + | + | + | + |
| Olfactory bulb- OB | + | + | + | - | - | - |
| **Ventral telencephalic area /Subpallium -S** | + | + | + | - | - | + |
| Dorsal division of ventral telencephalic area-**Sd** | + | + | + | - | - | + |
| Ventral division of ventral telencephalic area-**Sv** | + | + | + | - | - | + |
| **Dorsal telencephalic area /Pallium -P** | + | + | + | some | some | caudal |
| Migrated telencephalic area-**M4** | + | + | + | + | + | - |

| **miRNA** | ***miR-124***  **3dpf** | ***miR-124***  **5dpf** | ***miR-138***  **5dpf** | ***miR-219***  **3dpf** | ***miR-219***  **5dpf** | ***miR-135c***  **5dpf** |
| --- | --- | --- | --- | --- | --- | --- |
| DIENCEPHALON | + | + | + | + | + | + |
| Preoptic region-Po | + | + | rostral | few rostral | rostral | + |
| Subcommissural organ | - | - | - | - | + | - |
| Epithalamus | + | + | + | + | + | Periventricular cells |
| Pineal/epiphysis-**E** | + | + | - | - | - | + |
| Habenula-**Ha** | + | + | Lateral dorsal | ventral | ventral | Few ventral |
| **Thalamus** | + | + | + | + | + | + |
| Ventral thalamus -**VT** | + | + | + | + | + | + |
| Dorsal thalamus-**DT** | + | + | + | + | + | + |
| Eminentia thalami-**ET** | + | + | + |  | + | few |
| Migrated area of EmT-**M3** | + | + | - | - | - | - |
| Zona limitans intrathalamica-**ZLI** | - | - | - | - | - | - |
| Hypothalamus-H | + | + | + | - | - | + |
| Caudal hypothalamus-**Hc** | + | + | - | - | - | - |
| Diffuse nucleus of the inferior lobe-**DIL** | + | + | + | - | - | - |
| Area of hypothalamic lateral ventricular recess–**lr** | + | + | - | - | - | + |
| Intermediate hypothalamus –**Hi** | + | + | - | - | - | + |
| Lateral torus-**TLa** | + | + | + | - | - | - |
| Rostral hypothalamus-**Hr** | + | + | ventral | - | - | + |
| **Pituitary** | - | - | - | - | - | - |

| **miRNA** | ***miR-124***  **3dpf** | ***miR-124***  **5dpf** | ***miR-138***  **5dpf** | ***miR-219***  **3dpf** | ***miR-219***  **5dpf** | ***miR-135c***  **5dpf** |
| --- | --- | --- | --- | --- | --- | --- |
| Posterior tuberculum-PT | + | + | - | - | + | + |
| Dorsal part of periventricular posterior tuberculum-**PTd** | + | + | - | - | + | few |
| Migrated posterior tubercular area-**M2** | + | + | - | - | - | - |
| Ventral part of periventricular posterior tuberculum-**PTv** | + | + | - | - | - | + |
| **Retina** | + | + | - | - | - | - |
| Ciliary marginal zone-**CMZ** | - | - | - | - | - | - |
| Ganglion cell layer-**GCL** | + | + | - | - | - | - |
| Inner nuclear layer-**INL** | + | + | - | - | - | - |
| Photoreceptor layer-**Ph** | + | + | - | - | - | - |
| Pretectum | + | + | + | + | + | + |
| Periventricular pretectum-Pr | + | + | + | + | + | + |
| Migrated pretectal area-**M1** | + | + | - | Few caudal | Few caudal | few |
| **MIDBRAIN** | + | + | + | + | + | + |
| **Optic tectum-TeO** | + | + | + | + | + | + |
| Central zone-**cz** (5d) | + | + | - |  | - | - |
| Periventricular gray zone -**pgz** | + | + | some | few | few | some |
| Proliferative zone-**m** | - | - | - | - | - | - |
| Longitudinal torus-**TL**(5d) |  | - | - |  | - | - |
| Tegmentum-T | + | + | + | + | + | + |
| Region of the nucleus of medial longitudinal fascicle-**N** | + | + | - | + | + | + |
| Dorsal tegmental nucleus-**DTN (**5d) |  | + | - |  | + | + |
| Occulomotor nerve nucleus-**NIII** | + | + | - | + | + | - |
| Semicircular torus-**TS** | + | + | + | + | + | + |
| Interpeduncular nucleus-**NIn** | + | + | - | - | Few weak | - |

| **miRNA** | ***miR-124***  **3dpf** | ***miR-124***  **5dpf** | ***miR-138***  **5dpf** | ***miR-219***  **3dpf** | ***miR-219***  **5dpf** | ***miR-135c***  **5dpf** |
| --- | --- | --- | --- | --- | --- | --- |
| **HINDBRAIN** | + | + | + | + | + | + |
| **ISTHMUS** | + | + | lateral | + | + | + |
| Isthmic area including isthmic nucleus (**NI**) and lateral nucleus of valvula-**Is** | + | + | + | + | + | + |
| Locus coereleus-**LC** | + | + |  | + | + |  |
| Superior raphe-**SR** | + | + | - | + | + | - |
| Superior reticular formation-**SRF** | + | + | - | + | + | - |
| Trochlear motor nucleus-**NIV?** | + | + | - | + | + | - |
| Cerebellum | + | + | + | + | + | + |
| Cerebellar plate-CeP | + | + | Ganglionic layer | Some lateral | Some lateral | granular layer |
| Cerebellar crest-**CC** | - | - | - | - | - | - |
| Granular eminence-**EG** | + | + | - | + | + | + |
| Cerebellar valvula-**Va** | - | - | - | - | - | few |
| Medulla oblongata-MO | + | + | Medial and central part | +throughout | + throughout | Rostral :central DV columns  Lateral :scattered cells  Caudal : + throughout except most dorsal part |
| Rhombic lip | - | - | - | + | + | - |
| ***Lateral part*** (presumptive Octaval Area-**OA?)** | + | + | - | + | + | + |
| Dorsal part | + | + | +except most dorsal zone of cells | + | + | Few (periventricular) |
| Area postrema-**AP** | + | + |  | + | + |  |
| Facial lobe-LVII? | + | + |  | + | + | (periventricular) |
| Vagal lobe-LX? | + | + |  | + | + |  |
| Cranial nerve nuclei | + | + | - | + | + |  |
| Abducens motor nucleus-NVI | + | + | - | + | + |  |
| Facial motor nucleus-**NVII** | + | + | - | + | + |  |
| Glossopharyngeal motor nucleus-**NIX** | + | + | - | + | + |  |
| Trigeminal motor nucleus-**NV** | + | + | - | + | + |  |
| Vagal motor nucleus-**NX** | + | + | - | + | + |  |
| ***Ventral hindbrain areas*** | + | + | - | + | + | - |
| Inferior olive-**IO** | + | + | - | + | + | - |
| Inferior raphe-**IR** | + | + | - | + | + | - |
| Inferior reticular formation-**IRF** | + | + | - | + | + | - |
| Intermediate raphe-**IMR** | + | + | - | + | + | - |
| Intermediate reticular formation-**IMRF** | + | + | - | + | + | - |
| Mauthner cell-**MAC** | + | + | - | - | - | - |

| **miRNA** | ***miR-124***  **3dpf** | ***miR-124***  **5dpf** | ***miR-138***  **5dpf** | ***miR-219***  **3dpf** | ***miR-219***  **5dpf** | ***miR-135c***  **5dpf** |
| --- | --- | --- | --- | --- | --- | --- |
| **CRANIAL GANGLIA** | + | + | + | - | - | - |
| Anterior lateral line ganglion-**ALLG** | + | + | + | - | - | - |
| Facial ganglion-**FG** | + | + | + | - | - | - |
| Glossopharyngeal ganglion-**GG** | + | + | + | - | - | - |
| Octaval ganglion-**OG** | + | + | + | - | - | - |
| Posterior lateral line ganglion-**PLLG** | + | + | + | - | - | - |
| Trigeminal gangion-**TG** | + | + | + | - | - | - |
| Vagal ganglion-**VG** | + | + | + |  | - | - |

**Table C. *miR-128, miR-137, miR-181a, miR-181b* and *miR-153a* expression in the larval zebrafish brain.**

| miRNA | ***miR-128***  **3dpf** | ***miR-128***  **5dpf** | ***miR-137***  **5dpf** | ***miR-181a***  **3dpf** | ***miR-181b***  **5dpf** | ***miR-153a***  **5dpf** |
| --- | --- | --- | --- | --- | --- | --- |
| Periventricular cells | - | - | - | -/+ | -/+ | Few cases |
| Proliferative zones | - | - | - | -/+ | -/+ | - |
| Differentiating cells | + | + | + | + | + | + |
| Area-specific | yes | yes | yes | Quantitative differences in expression | Quantitative differences in expression | Quantitative differences in expression |
| Expression pattern conserved/different between embryo and adult | Mainly conserved | Mainly conserved | Mainly conserved | Mainly conserved | Mainly conserved | Mixed profile |

| miRNA | ***miR-128***  **3dpf** | ***miR-128***  **5dpf** | ***miR-137***  **5dpf** | ***miR-181a***  **3dpf** | ***miR-181b***  **5dpf** | ***miR-153a***  **5dpf** |
| --- | --- | --- | --- | --- | --- | --- |
| Olfactory epithelium | - | - | Few cells at ventromedial edge | - | - | - |
| TELENCEPHALON | + | + | + | + | + | + |
| Olfactory bulb- OB | + | + | - | Weak | Weak | - |
| **Ventral telencephalic area /Subpallium -S** | + except rostro-ventral cells | + except rostro-ventral cells | + | Weak | Weak | + |
| Dorsal division of ventral telencephalic area-**Sd** | + | + | + | Weak | Weak | + |
| Ventral division of venral telencephalic area-**Sv** | + except rostral cells | + except rostral cells | + | Weak | Weak | + |
| **Dorsal telencephalic area /Pallium -P** | +  caudal : strong | +  caudal : strong | Rostral:Ventral /medial : +  Lateral : few/weak.  Caudal : + | Weak except strong at central area (limit with Sd and OB) | Weak except  strong at central area (limit with Sd and OB) | Some Weak |
| Migrated telencephalic area-**M4** | + | + | + | Weak | - | - |

| miRNA | ***miR-128***  **3dpf** | ***miR-128***  **5dpf** | ***miR-137***  **5dpf** | ***miR-181a***  **3dpf** | ***miR-181b***  **5dpf** | ***miR-153a***  **5dpf** |
| --- | --- | --- | --- | --- | --- | --- |
| DIENCEPHALON | + | + | + | Weak | Weak | +including periventricular cells |
| Preoptic region-Po | - | Rostral | + (Caudal : weak) | Weak | Weak | + |
| Subcommissural organ | - | - | - | Weak | Weak | - |
| Epithalamus | + | + | - | Weak | Weak | + |
| Pineal/epiphysis-**E** | - | - | - | - | - | - |
| Habenula-**Ha** | Lateral | Lateral | - | Weak | Weak | + (strong expressing cells) |
| **Thalamus** | + | + | + | Weak | Weak | + |
| Ventral thalamus -**VT** | + | + | Some weak | Weak | Weak | + |
| Dorsal thalamus-**DT** | - | - | + | Weak | Weak | + (except caudo-lateral) |
| Eminentia thalami-**ET** | Few lateral | Few lateral | + | Weak | Weak | + |
| Migrated area of ET-**M3** | - | - | - | - | - | + (strong expressing cells) |
| Zona limitans intrathalamica-**ZLI** | - | - | - | - | - | + |
| Hypothalamus-H | + | + | + | Weak | Weak | + (including periventricular cells) |
| Caudal hypothalamus-**Hc** | - | - | Few | Few weak | Weak | + (including periventricular cells) |
| Diffuse nucleus of the inferior lobe-**DIL** | + | + | Few | Weak | Weak | + |
| Area of hypothalamic lateral recess ventricle–**lr** | + | + | - | Weak | Weak | + |
| Intermediate hypothalamus-**Hi** | + | Lateral ventral | Ventral | Weak | Weak | + (including periventricular cells) |
| Lateral torus-**TLa** | + | + | + | Weak |  | + |
| Rostral hypothalamus-**Hr** | + | Lateral ventral | + | Weak | Weak | + |
| **Pituitary** | - | - | - | - | - | - |

| miRNA | ***miR-128***  **3dpf** | ***miR-128***  **5dpf** | ***miR-137***  **5dpf** | ***miR-181a***  **3dpf** | ***miR-181b***  **5dpf** | ***miR-153a***  **5dpf** |
| --- | --- | --- | --- | --- | --- | --- |
| Posterior tuberculum-PT | + | + | + | Weak | Weak | + |
| Dorsal part of periventricular posterior tuberculum-**PTd** | - | - | Few weak | Weak | Weak | + |
| Migrated posterior tubercular area-**M2** | + | + | - | Weak | Weak | Weak |
| Ventral part of periventricular posterior tuberculum-**PTv** | + | + | + | Weak | Weak | + |
| **Retina** | - | - | - | + | + | - |
| Ciliary marginal zone-**CMZ** | - | - | - | - | - | - |
| Ganglion cell layer-**GCL** | - | - | - | + | + | - |
| Inner nuclear layer-**INL** | - | - | - | + (inner part) | + (inner part) | - |
| Photoreceptor layer-**Ph** | - | - | - | - | - | - |
| Pretectum | + | + | - | + | + | + |
| Periventricular pretectum-Pr | - | - | - | Weak | Weak | + |
| Migrated pretectal area-**M1** | + | + | - | + | Some | - |
| **MIDBRAIN** | + | + | + | + | + | + |
| **Optic tectum-TeO** | + | + | - | + | + | + |
| Central zone-**cz** (5d) |  | + | - | + | Some | - |
| Periventricular zone -**pgz** | + | + | - | + | + | + |
| Proliferative zone-**m** | - | - | - | + | + | - |
| Longitudinal torus-**TL** (5d) |  | + | - |  | Weak | - |
| Tegmentum-T | + | + | + | Weak | Weak | + |
| Region of the nucleus of medial longitudinal fascicle-**N** | - | - | - | Some weak | Some weak | + |
| Dorsal tegmental nucleus-**DTN** | - | - | + |  |  | + |
| Occulomotor nerve nucleus-**NIII** | - | - | - | - | - | + |
| Semicircular torus-**TS** | + | + | Caudal | Weak | Weak | + |
| Interpeduncular nucleus-**NIn** | - | - | - |  |  | + |

| miRNA | ***miR-128***  **3dpf** | ***miR-128***  **5dpf** | ***miR-137***  **5dpf** | ***miR-181a***  **3dpf** | ***miR-181b***  **5dpf** | ***miR-153a***  **5dpf** |
| --- | --- | --- | --- | --- | --- | --- |
| **HINDBRAIN** | + | + | + | + | + | + |
| **ISTHMUS** | + | + | + | Weak | Weak | + |
| Isthmic area including isthmic nucleus (**NI**) and nucleus lateralis valvula-**Is** | + | + | Dorso-ventral rows of cells, NI | Weak | Some weak | + |
| Locus coereleus-**LC** | - | - | - | - | - | - |
| Superior raphe-**SR** | - | - | - | - | - | + |
| Superior reticular formation-**SRF** | - | - | - | Weak | Weak | - |
| Cerebellum | + | + | - | Weak | Weak | - |
| Cerebellar plate-CeP | + | + | - | Weak | Some weak | - |
| Cerebellar crest-**CC** | - | - | - | - | - | - |
| Granular eminence-**EG** | Few | Few | - | Weak | Weak | - |
| Cerebellar valvula-**Va** | - | - | - | Weak | Weak | - |
| ***Rhombic lip*** | - | - | - | - | - | - |
| Medulla oblongata-MO | Lateral  throughout | Lateral  throughout | Lateral and medial rows of cells | Weak throughout  except strong  caudal dorsal/central  column of cells | Weak throughout  except strong  caudal dorsal/central  column of cells | Rostral :medial column  Caudal :medial column and lateral weak cells |
| ***Lateral part*** (including octaval area-**OA ?)** | +(strong) | +(strong) | - | weak | Some weak |  |
| Dorsal part | + | + | + | + | + |  |
| Area postrema-**AP?** | + and commissural nucleus of cajal (NC?) | + and commissural nucleus of cajal (NC?) | + |  |  |  |
| Facial lobe-LVII? | + | + |  |  |  |  |
| Vagal lobe-LX? | + | + | (+) |  |  |  |
| Cranial nerve nuclei | - | - | + |  |  |  |
| Abducens nucleus-NVI | - | - | - |  |  |  |
| Facial motor nucleus-**NVIIm** | - | - | - |  |  |  |
| Glossopharyngeal motor nucleus-**NIX** | - | - | - |  |  |  |
| Trigeminal motor nucleus-**NVm** | - | - | - |  |  |  |
| Vagal motor nucleus-**NXm** | - | - | + |  |  |  |
| ***Ventral hindbrain areas*** | + | + | - | Weak | Weak | - |
| Inferior olive-**IO** | - | - | - | - | - | - |
| Inferior raphe-**IR** | - | - | - | - | - | - |
| Inferior reticular formation-**IRF** | Few | Few | - | - | - | - |
| Intermediate raphe-**IMR** | - | - | - | - | - | - |
| Intermediate reticular formation-**IMRF** | - | - | - | Weak | Few weak | - |
| Mauthner cell-**MAC** | - | - | - | - | - | - |

| **miRNA** | ***miR-128***  **3dpf** | ***miR-128***  **5dpf** | ***miR-137***  **5dpf** | ***miR-181a***  **3pdf** | ***miR-181b***  **5dpf** | ***miR-153a***  **5dpf** |
| --- | --- | --- | --- | --- | --- | --- |
| **CRANIAL GANGLIA** | - | - | + | + | + | - |
| Anterior lateral line ganglion-**ALLG** | - | - | - |  |  | - |
| Facial ganglion-**FG** | - | - | Weak |  |  | - |
| Glossopharyngeal ganglion-**GG** | - | - | - |  |  | - |
| Octaval ganglion-**OG** | - | - | - |  | - | - |
| Posterior lateral line ganglion-**PLLG** | - | - | - | Weak |  | - |
| Trigeminal gangion-**TG** | - | - | Weak |  | - | - |
| Vagal ganglion-**VG** | - | - | - | Weak | + | - |

**Table D. *miR-34, miR-222, miR-221* and *miR-7* expression in the larval zebrafish brain.**

| miRNA | ***miR-34***  **3dpf/5dpf** | ***miR-222***  **5dpf** | ***miR-221***  **5dpf** | ***miR-7***  **5dpf** |
| --- | --- | --- | --- | --- |
| Periventricular cells | - | - | - | - |
| Proliferative zones | - | - | - | - |
| Differentiating cells | + | + | + | + |
| Area-specific | Caudal brain | Rostral brain | yes | Forebrain areas and area postrema |
| Pattern conserved between embryo and adult | no | Mixed profile | Mixed profile | Mainly yes |

| miRNA | ***miR-34***  **3dpf/5dpf** | ***miR-222***  **5dpf** | ***miR-221***  **5dpf** | ***miR-7***  **5dpf** |
| --- | --- | --- | --- | --- |
| Olfactory epithelium | - | - | - | - |
| TELENCEPHALON | - | + | + | + |
| Olfactory bulb- OB | - | - | - | - |
| **Ventral telencephalic area /Subpallium -S** | - | + | Few weak | + |
| Dorsal division of ventral telencephalic area-**Sd** | - | + | + | + |
| Ventral division of venral telencephalic area-**Sv** | - | + | + | + |
| **Dorsal telencephalic area /Pallium -P** | - | + | Rostral :few lateral and ventral  Caudal :+ | +  caudal :weak |
| Migrated telencephalic area-**M4** | - | - | - | - |

| miRNA | ***miR-34***  **3dpf/5dpf** | ***miR-222***  **5dpf** | ***miR-221***  **5dpf** | ***miR-7***  **5dpf** |
| --- | --- | --- | --- | --- |
| DIENCEPHALON | - | + | + | + |
| Preoptic region-Po | - | Weak | - | +  caudal :weak |
| Subcommissural organ | - | - | - | - |
| Epithalamus | - | - | - | - |
| Pineal/epiphysis-**E** | - | - | - | - |
| Habenula-**Ha** | - | - | - | - |
| **Thalamus** | - | + | Weak scattered cells | + |
| Ventral thalamus -**VT** | - | Weak |  | - |
| Dorsal thalamus-**DT** | - | - |  | Weak |
| Eminentia thalami-**ET** | - | + | + | + |
| Migrated area of ET-**M3** | - | - | - | - |
| Zona limitans intrathalamica-**ZLI** | - | - | - | - |
| Hypothalamus-H | - | + | +strong | + |
| Caudal hypothalamus-**Hc** | - | - | - | Ventral |
| Diffuse nucleus of the inferior lobe-**DIL** | - | + | + | - |
| Area of hypothalamic lateral ventricular recess–**lr** | - | + | Lateral | Ventral |
| Intermediate hypothalamus –**Hi** | - | + | Lateral | Ventral |
| Lateral torus-**TLa** | - | + | + | - |
| Rostral hypothalamus-**Hr** | - | + | + | Ventral |
| **Pituitary** | - | - | - | - |

| miRNA | ***miR-34***  **3dpf/5dpf** | ***miR-222***  **5dpf** | ***miR-221***  **5dpf** | ***miR-7***  **5dpf** |
| --- | --- | --- | --- | --- |
| Posterior tuberculum-PT | - | + | Few | - |
| Dorsal part of periventricular posterior tuberculum-**PTd** | - | - | Few | - |
| Migrated posterior tubercular area-**M2** | - | Lateral,  ventral | - | - |
| Ventral part of periventricular posterior tuberculum-**PTv** | - | + | - | - |
| **Retina** | - | - | - | - |
| Ciliary marginal zone-**CMZ** | - | - | - | - |
| Ganglion cell layer-**GCL** | - | - | - | - |
| Inner nuclear layer-**INL** | - | - | - | - |
| Photoreceptor layer-**Ph** | - | - | - | - |
| Pretectum | - | - | - | - |
| Periventricular pretectum-Pr | - | - | - | - |
| Migrated pretectal area-**M1** | - | - | - | - |
| **MIDBRAIN** | - | + | + | - |
| **Optic tectum-TeO** | - | + | + | - |
| Central zone-**cz** | - | - | - | - |
| Periventricular zone -**pgz** | - | Few weak scattered cells | Few weak rostral cells | - |
| Proliferative zone-**m** | - | - | - | - |
| Longitudinal torus-**TL** (5d) | - | - | - | - |
| Tegmentum-T | - | - | Few | - |
| Region of the nucleus of medial longitudinal fascicle-**N** | - | - | - | - |
| Dorsal tegmental nucleus-**DTN** | - | - | - | - |
| Occulomotor nerve nucleus-**NIII** | - | - | - | - |
| Semicircular torus-**TS** | - | - | Few weak | - |
| Interpeduncular nucleus-**NIn** | - | - | - | - |

| miRNA | ***miR-34***  **3dpf/5dpf** | ***miR-222***  **5dpf** | ***miR-221***  **5dpf** | ***miR-7***  **5dpf** |
| --- | --- | --- | --- | --- |
| **HINDBRAIN** | + | - | - | - |
| **ISTHMUS** | + | - | - | - |
| Isthmic area including isthmic nucleus (**NI**) and nucleus lateralis valvula-**Is** | - | - | - | - |
| Locus coereleus-**LC** | + | - | - | - |
| Trochlear nucleus-**NIV?** | - | - | - | - |
| Superior raphe-**SR** | - | - | - | - |
| Superior reticular formation-**SRF** | + | - | - | - |
| Cerebellum | - | - | - | - |
| Cerebellar plate-CeP | - | - | - | - |
| Cerebellar crest-**CC** | - | - | - | - |
| Granular eminence-**EG** | - | - | - | - |
| Cerebellar valvula-**Va** | - | - | - | - |
| ***Rhombic lip*** | - | - | - | - |
| Medulla oblongata-MO | Groups of cells Ventral/lateral | - | - | + |
| ***Lateral part*** (presumptive Octaval area-**OA?)** | + | - | - | - |
| Dorsal part | - | - | - | + |
| Area postrema-**AP** | - | - | - | + |
| Facial lobe-LVII? | - | - | - | - |
| Vagal lobe-LX? | - | - | - | - |
| Cranial nerve nuclei | + ? | - | - | - |
| Abducens nucleus-NVI | - | - | - | - |
| Facial motor nucleus-**NVII** | - | - | - | - |
| Glossopharyngeal motor nucleus-**NIX** | - | - | - | - |
| Trigeminal motor nucleus-**NV** | + ? | - | - | - |
| Vagal motor nucleus-**NX** | - | - | - | - |
| ***Ventral hindbrain areas*** | + | - | - | - |
| Inferior olive-**IO** | - | - | - | - |
| Inferior raphe-**IR** | - | - | - | - |
| Inferior reticular formation-**IRF** | + | - | - | - |
| Intermediate raphe-**IMR** | - | - | - | - |
| Intermediate reticular formation-**IMRF** | + | - | - | - |
| Mauthner cell-**MAC** | + | - | - | - |

| miRNA | ***miR-34***  **3dpf/5dpf** | ***miR-222***  **5dpf** | ***miR-221***  **5dpf** | ***miR-7***  **5dpf** |
| --- | --- | --- | --- | --- |
| **CRANIAL GANGLIA** | + | - | - | - |
| Anterior lateral line ganglion-**ALLG** | - | - | - | - |
| Facial Ganglion-**FG** | - | - | - | - |
| Glossopharyngeal ganglion-**GG** | - | - | - | - |
| Octaval ganglion-**OG** | Few cells | - | - | - |
| Posterior lateral line ganglion-**PLLG** | - | - | - | - |
| Trigeminal gangion-**TG** | Few cells | - | - | - |
| Vagal ganglion-**VG** | - | - | - | - |

**Table E. *miR-218a, miR-183, miR-182, miR-96, miR-375, miR-200a, miR-200b* and *miR-429* expression in the larval zebrafish brain.**

| miRNA | ***miR-218a***  **3dpf** | ***miR-218a***  **5dpf** | ***miR-183***  **5dpf** | ***miR-182***  **5dpf** | ***miR-96***  **5dpf** | ***miR-375***  **5dpf** | ***miR-200a***  ***miR-200b***  ***miR-429***  **3dpf/5dpf** |
| --- | --- | --- | --- | --- | --- | --- | --- |
| Periventricular cells | - | - | - | - | - | - | - |
| Proliferative zones | - | - | - | - | - | - | - |
| Differentiated cells | + | + | + | + | + | + | + |
| Cell-specific | Motoneurons | Motoneurons | Olfactory epithelium  Epithalamus  Retina cells  Neuromasts  Otic capsule hair cells  Cranial ganglia | Olfactory epithelium  Epithalamus  Retina cells  Neuromasts  Otic capsule hair cells  Cranial ganglia | Olfactory epithelium  Epithalamus  Retina cells  Neuromasts  Otic capsule hair cells  Cranial ganglia | Pituitary  Few hypothalamic cells  Cranial ganglia | Taste buds  Olfactory epithelium  Neuromasts |
| Expression pattern conserved between embryo and adult | No | No |  |  |  |  |  |

| miRNA | ***miR-218a***  **3dpf** | ***miR-218a***  **5dpf** | ***miR-183***  **5dpf** | ***miR-182***  **5dpf** | ***miR-96***  **5dpf** | ***miR-375***  **5dpf** | ***miR-200a***  ***miR-200b***  ***miR-429***  **3dpf/5dpf** |
| --- | --- | --- | --- | --- | --- | --- | --- |
| Olfactory epithelium | - | - | + | + | + | - | + |
| TELENCEPHALON | - | - | - | - | - | - | - |
| Olfactory bulb- OB | - | - | - | - | - | - | - |
| **Ventral telencephalic area /Subpallium -S** | - | - | - | - | - | - | - |
| Dorsal division of ventral telencephalic area-**Sd** | - | - | - | - | - | - | - |
| Ventral division of venral telencephalic area-**Sv** | - | - | - | - | - | - | - |
| **Dorsal telencephalic area /Pallium -P** | - | - | - | - | - | - | - |
| Migrated telencephalic area-**M4** | - | - | - | - | - | - | - |

| miRNA | ***miR-218a***  **3dpf** | ***miR-218a***  **5dpf** | ***miR-183***  **5dpf** | ***miR-182***  **5dpf** | ***miR-96***  **5dpf** | ***miR-375***  **5dpf** | ***miR-200a***  ***miR-200b***  ***miR-429***  **3dpf/5dpf** |
| --- | --- | --- | --- | --- | --- | --- | --- |
| DIENCEPHALON | - | - | + | + | + | + | - |
| Preoptic region-Po | - | - | - | - | - | - | - |
| Subcommissural organ | - | - | - | - | - | - | - |
| Epithalamus | - | - | + | + | + | - | - |
| Pineal/epiphysis-**E** | - | - | +,  parapineal  organ | +,  parapineal  organ ? | +,  parapineal  organ ? | - | - |
| Habenula-**Ha** | - | - | - | - | - | - | - |
| **Thalamus** | - | - | - | - | - | - | - |
| Ventral thalamus -**VT** | - | - | - | - | - | - | - |
| Dorsal thalamus-**DT** | - | - | - | - | - | - | - |
| Eminentia thalami-**ET** | - | - | - | - | - | - | - |
| Migrated area of ET-**M3** | - | - | - | - | - | - | - |
| Zona limitans intrathalamica-**ZLI** | - | - | - | - | - | - | - |
| Hypothalamus-H | - | - | - | - | - | Few scattered | - |
| Caudal hypothalamus-**Hc** | - | - | - | - | - | - | - |
| Diffuse nucleus of the inferior lobe-**DIL** | - | - | - | - | - | - | - |
| Area of hypothalamic lateral recess ventricle–**lr** | - | - | - | - | - | - | - |
| Intermediate hypothalamus –**Hi** | - | - | - | - | - | + | - |
| Lateral torus-**TLa** | - | - | - | - | - | - | - |
| Rostral hypothalamus-**Hr** | - | - | - | - | - | + | - |
| **Pituitary** | - | - | - | - | - | + | - |

| miRNA | ***miR-218a***  **3dpf** | ***miR-218a***  **5dpf** | ***miR-183***  **5dpf** | ***miR-182***  **5dpf** | ***miR-96***  **5dpf** | ***miR-375***  **5dpf** | ***miR-200a***  ***miR-200b***  ***miR-429***  **3dpf/5dpf** |
| --- | --- | --- | --- | --- | --- | --- | --- |
| Posterior tuberculum-PT | - | - | - | - | - | - | - |
| Dorsal part of periventricular posterior tuberculum-**PTd** | - | - | - | - | - | - | - |
| Migrated posterior tubercular area-**M2** | - | - | - | - | - | - | - |
| Ventral part of periventricular posterior tuberculum-**PTv** | - | - | - | - | - | - | - |
| **Retina** | - | - | + | + | + | - |  |
| Ciliary marginal zone-**CMZ** | - | - | - | - | - | - | - |
| Ganglion cell layer-**GCL** | - | - | - | - | - | - | - |
| Inner nuclear layer-**INL** | - | - | + | + | + | - | - |
| Photoreceptor layer-**Ph** | - | - | + | + | + | - | - |
| Pretectum | - | - | - | - | - | - | - |
| Periventricular pretectum-Pr | - | - | - | - | - | - | - |
| Migrated pretectal area-**M1** | - | - | - | - | - | - | - |
| **MIDBRAIN** | + | + | - | - | - | - | - |
| **Optic tectum-TeO** | - | - | - | - | - | - | - |
| Central zone-**cz** | - | - | - | - | - | - | - |
| Periventricular zone -**pgz** | - | - | - | - | - | - | - |
| Proliferative zone-**m** | - | - | - | - | - | - | - |
| Longitudinal torus-**TL** (5d) | - | - | - | - | - | - | - |
| Tegmentum-T | + | + | - | - | - | - | - |
| Region of the nucleus of medial longitudinal fascicle-**N** | - | - | - | - | - | - | - |
| Dorsal tegmental nucleus-**DTN** | - | - | - | - | - | - | - |
| Occulomotor nerve nucleus-**NIII** | + | + | - | - | - | - | - |
| Semicircular torus-**TS** | - | - | - | - | - | - | - |
| Interpeduncular nucleus-**NIn** | - | - | - | - | - | - | - |

| miRNA | ***miR-218a***  **3dpf** | ***miR-218a***  **5dpf** | ***miR-183***  **5dpf** | ***miR-182***  **5dpf** | ***miR-96***  **5dpf** | ***miR-375***  **5dpf** | ***miR-200a***  ***miR-200b***  ***miR-429***  **3dpf/5dpf** |
| --- | --- | --- | --- | --- | --- | --- | --- |
| **HINDBRAIN** | + | + | - | - | - | - | - |
| **ISTHMUS** | + | + | - | - | - | - | - |
| Isthmic area including isthmic nucleus and nucleus lateralis valvula-**Is** | - | - | - | - | - | - | - |
| Locus coereleus-**LC** | - | - | - | - | - | - | - |
| Superior raphe-**SR** | - | - | - | - | - | - | - |
| Superior reticular formation-**SRF** | - | - | - | - | - | - | - |
| Trochlear motor nucleus-**NIV?** |  |  |  |  |  |  |  |
| Cerebellum | - | - | - | - | - | - | - |
| Cerebellar plate-Ce | - | - | - | - | - | - | - |
| Cerebellar crest-**CC** | - | - | - | - | - | - | - |
| Granular eminence-**EG** | - | - | - | - | - | - | - |
| Cerebellar valvula-**Va** | - | - | - | - | - | - | - |
| Medulla oblongata-MO | + | + | - | - | - | - | - |
| ***Lateral part*** (presumptive octaval area-**OA ?)** | - | - | - | - | - | - | - |
| Dorsal part | - | - | - | - | - | - | - |
| Area postrema-**AP?** | - | - | - | - | - | - | - |
| Facial lobe-LVII? | - | - | - | - | - | - | - |
| Glossopharyngeal lobe-LIX? | - | - | - | - | - | - | - |
| Vagal lobe-LX? | - | - | - | - | - | - | - |
| Cranial nerve nuclei | + | + | - | - | - | - | - |
| Abducens nucleus-NVI | + | + | - | - | - | - | - |
| Facial motor nucleus-**NVII** | + | + | - | - | - | - | - |
| Glossopharyngeal motor nucleus-**NIX** | + | + | - | - | - | - | - |
| Trigeminal motor nucleus-**NV** | + | + | - | - | - | - | - |
| Vagal motor nucleus-**NX** | + | + | - | - | - | - | - |
| ***Ventral hindbrain areas*** | - | - | - | - | - | - | - |
| Inferior olive-**IO** | - | - | - | - | - | - | - |
| Inferior raphe-**IR** | - | - | - | - | - | - | - |
| Inferior reticular formation-**IRF** | - | - | - | - | - | - | - |
| Intermediate raphe-**IMR** | - | - | - | - | - | - | - |
| Intermediate reticular formation-**IMRF** | - | - | - | - | - | - | - |
| Mauthner cell-**MAC** | - | - | - | - | - | - | - |

| miRNA | ***miR-218a***  **3dpf** | ***miR-218a***  **5dpf** | ***miR-183***  **5dpf** | ***miR-182***  **5dpf** | ***miR-96***  **5dpf** | ***miR-375***  **5dpf** | ***miR-200a***  ***miR-200b***  ***miR-429***  **3dpf/5dpf** |
| --- | --- | --- | --- | --- | --- | --- | --- |
| **CRANIAL GANGLIA** | - | - | + | + | + | + | - |
| Anterior lateral line ganglion-**ALLG** | - | - | + | + | + | + | - |
| Facial ganglion-**FG** | - | - | + | + | + | + | - |
| Glossopharyngeal ganglion-**GG** | - | - | + | + | + | + | - |
| Octaval ganglion-**OG** | - | - | + | + | + | + | - |
| Posterior lateral line ganglion-**PLLG** | - | - | + | + | + | + | - |
| Trigeminal gangion-**TG** | - | - | + | + | + | + | - |
| Vagal ganglion-**VG** | - | - | + | + | + | + | - |

**Table F. *miR-92b, miR-9, miR-7b* and *miR-7c* expression in the adult zebrafish brain.**

| miRNA | *miR-92b*Ad | *miR-9*Ad | *let-7b*Ad | *let-7c*Ad |
| --- | --- | --- | --- | --- |
| Periventricular cells | + | + | + | + |
| Proliferative zones | + | + | + | + |
| Differentiating cells | - | + | + | + |
| Specific areas | Throughout the brain | Throughout the brain | No | No |
| Conserved/ different from embryo | Cell type conservation | Cell type conservation | Cell type conservation | Cell type conservation |

| miRNA | *miR-92b*Ad | ***miR-9***  **Ad** | *let-7b* **Ad** | *let-7c* **Ad** |
| --- | --- | --- | --- | --- |
| **TELENCEPHALON-Te** | **Periventricular and adjacent cells** | + | +  including periventricular cells | +  including periventricular cells |
| **Olfactory bulb- OB** | Few periventricular and adjacent cells | + | + | + |
| External cellular layer (including mitral cells)-**ECL** | Few | + | + | + |
| Glomerular layer-**GL** | - | + | + | + |
| Primary olfactory fiber layer-**POF** | - | - | - | - |
| Internal cellular layer of olfactory bulb-**ICL** | Few | + | + | + |
| **Ventral telencephalic area /Subpallium –V** | Periventricular and adjacent cells | + | + | + |
| Central nucleus of ventral telencephalic area-**Vc** | - | + | + | + |
| Dorsal nucleus of ventral telencephalic area-**Vd** | + | Periventricular and adjacent cells | - | - |
| Lateral nucleus of ventral telencephalic area-**Vl** | - | + | + | + |
| Ventral nucleus of venral telencephalic area-**Vv** | Few weak | + | + | + |
| Supracommissural nucleus of ventral telencephalic area-**Vs** | + | Few periventricular cells | - | - |
| Postcommissural nucleus of ventral telencephalic area-**Vp** | + | + | - | - |
| **Dorsal telencephalic area /Pallium -P** | Periventricular and adjacent cells | + | + | + |
| Central zone of dorsal telencephalic area-**Dc** | - | Some | - | - |
| Dorsal zone of dorsal telencephalic area-**Dd** | - | + | Periventricular  and adjacent cells | Periventricular and adjacent cells |
| Lateral zone of dorsal telencephalic area-**Dl** | + | + | + | + |
| Medial zone of dorsal telencephalic area-**Dm** | + | + | + | + |
| Posterior zone of dorsal telencephalic area-**Dp** | + | + | + | + |
| Nucleus taenia-**NT** | - | + | + | + |
|  |  |  |  |  |
| Endopeduncular nucleus, dorsal part **ENd** | - | + | + | + |
| Endopeduncular nucleus, ventral part **ENv** | - | - | + | + |

| miRNA | *miR-92b*Ad | *miR-9* **Ad** | *let-7b* **Ad** | *let-7c* **Ad** |
| --- | --- | --- | --- | --- |
| DIENCEPHALON | Periventricular and adjacent cells | + | + including periventricular cells | +  including periventricular cells |
| Preoptic area | Periventricular and adjacent cells | + | + | + |
| Parvocellular preoptic nucleus, anterior part-**PPa** | + | + | + | + |
| Parvocellular preoptic nucleus, posterior part-**PPp** | + | Periventricular cells | + | + |
| Magnocellular preopic nucleus **PM** | - | + | - | - |
| Suprachiasmatic nucleus **SC** | - | Periventricular cells | + | + |
| Epithalamus | Periventricular and adjacent cells | + | + | + |
| Pineal/epiphysis-**E** | - | - | + | + |
| Dorsal Habenular nucleus-**Had** | + | Few cells | + | + |
| Ventral Habenular nucleus-**Hav** | + | - | - | - |
| **Thalamus** | Periventricular and adjacent cells | + | - | - |
| Anterior thalamic nucleus-**A** | + | + | - | - |
| Ventromedial thalamic nucleus-**VM** | + | + | - | - |
| Ventrolateral thalamic nucleus-**VL** | - | - | - | - |
| Central posterior thalamic nucleus-**CP** | + | + | - | - |
| Dorsal posterior thalamic nucleus-**DP** | - | + | - | - |
| Intermediate thalamic nucleus-**I** | + | + | + | + |
| Hypothalamus | Periventricular and adjacent cells | Mainly periventricular cells | + | + |
| Anterior tuberal nucleus-**ATN** | - | - | + | + |
| Caudal zone of periventricular hypothalamus-**Hc** | + | + | + | + |
| Central nucleus of the inferior lobe-**CIL** | - | - | - | - |
| Diffuse nucleus of the inferior lobe-**DIL** | - | Few scattered cells | + | + |
| Dorsal zone of periventricular hypothalamus-**Hd** | + | Few | + | + |
| Dorsal zone of periventricular hypothalamus- Lateral recess **Hd-lr** | + | +mainly periventricular  cells | + | + |
| Lateral hypothalamic nucleus-**LH** | - | Few | + | + |
| Lateral torus-**TLa** | + | Few scattered | + | + |
| Mammilary body-**CM** | + | + | + | + |
| Ventral zone of periventricular hypothalamus-**Hv** | + | + | + | + |

| miRNA | *miR-92b* **Ad** | *miR-9* **Ad** | *let-7b* **Ad** | *let-7c* **Ad** |
| --- | --- | --- | --- | --- |
| Posterior tuberculum | Periventricular and adjacent cells | + | + | + |
| Anterior preglomerular nucleus-**PGa** | - | + | + | + |
| Caudal preglomerular nucleus-**PGc** | - | - | - | - |
| Lateral preglomerular nucleus-**PGl** | - | + | + | + |
| Medial preglomerular nucleus-**PGm** | + | + | + | + |
| Nucleus of Paraventricular organ-**nPVO** | + | - | - | - |
| Periventricular nucleus of posterior tuberculum-**TPp** | + | Periventricular cells | - | - |
| Posterior thalamic nucleus-**Pt** | - | + | + | + |
| Posterior tuberal nucleus-**PTN** | + | - | + | + |
| Subglomerular nucleus-**SG** | - | - | + | + |
| Tertiary gustatory nucleus-**TGN** | - | - | + | + |
| Pretectum | Periventricular and adjacent cells | + | + | + |
| Accessory pretectal nucleus-**APN** | - | + | - | - |
| Central pretectal nucleus-**CPN** | - | + | - | - |
| Magnocellular superficial pretectal nucleus-**PSm** | - | few | + | + |
| Parvocellular superficial pretectal nucleus-**PSp** | - | + | + | + |
| Periventricular pretectal nucleus, dorsal part-**PPd** | + | + | - | - |
| Periventricular pretectal nucleus, ventral part-**PPv** | + | + | - | - |
| Dorsal accessory optic nucleus-**DAO** | - | - | + | + |
| Ventral accessory optic nucleus-**VAO** | - | - | + | + |
| Posterior preoptic nucleus-**PO** | - | + | - | - |
| **MIDBRAIN** | + | + | + | + |
| **Optic tectum-TeO** | + | + | + | + |
| Superficial white zone-**swz** | - | - | - | - |
| Superficial grey zone-**sgz** | - | Few | + | + |
| Central zone-**cz** | - | Few | - | - |
| Deep white zone-**dwz** | - | - | - | - |
| Periventricular gray zone of optic tectum-**pgz (layers 1,2,3)** | Some | + | Some | Some |
| Proliferative zone-**m** | + | + | + | + |
| Longitudinal torus-**TL** | Few | + | Few | Few |
| Tegmentum | Throughout periventricular and adjacent cells | + | + | + |
| Central nucleus of semicircular torus-**TSc** | + | Periventricular cells | Caudal | Caudal |
| Ventrolateral nucleus of semicircular torus-**TSvl** | + | Periventricular cells | + | + |
| Dorsal tegmental nucleus-**DTN** | + | Few | - | - |
| Mesencephalic nucleus of trigeminal nerve-**MNV** | - | Few ventral | - | - |
| Nucleus of the medial longitudinal fascicle-**nMLF** | - | - | - | - |
| Oculomotor nucleus-**NIII** | - | - | - | - |
| Rostral tegmental nucleus-**RT** | - | - | - | - |
| Interpeduncular nucleus-**NIn** | Few | - | - | - |

| miRNA | *miR-92b* **Ad** | *miR-9* **Ad** | *let-7b* **Ad** | *let-7c* **Ad** |
| --- | --- | --- | --- | --- |
| **HINDBRAIN** | Periventricular cells around rhombencephalic ventricle (rv) | +  including periventricular cells around the rhombencephalic ventricle (rv) | +  including periventricular cells around the rhombencephalic ventricle (rv) | +  including periventricular cells around the rhombencephalic ventricle (rv) |
| **Isthmus** | Periventricular cells of the rhombencephalic ventricle | + | + | + |
| Isthmic nucleus-**NI** | - | - | + | + |
| Nucleus of lateral valvula-**NLV** | + | Caudal | - | - |
| Locus coeruleus-**LC** | - | - | - | - |
| Nucleus of the lateral lemniscus-**NLL** | - | - | - | - |
| Perilemniscal nucleus-**PL** | - | + | - | - |
| Superior raphe-**SR** | - | Ventral | + | + |
| Superior reticular formation-**SRF** | - | + | - | - |
| Cerebellum-Ce | + | + | + | + |
| Caudal lobe of cerebellum-**LCa** | Medial part | + | + | + |
| Cerebellar corpus-**CCe** | Medial molecular and granular layers | Molecular (CeML), granular (CeGL) and ganglionic (CeGaL) layers : scattered cells | + | + |
| Cerebellar crest-**CC** | - | Scattered | + | + |
| Granular eminence-**EG** | Few | + | + | + |
| Lateral division of cerebellar valvula -**Val** | Few | + | - | - |
| Medial division of cerebellar valvula -**Vam** | Few | + | - | - |
| **Cranial nerve nuclei** | - | + | + | + |
| Abducens nuclei-**NVI** | - | - | - | - |
| Facial motor nucleus-**NVIIm** | - | - | - | - |
| Glossopharyngeal motor nucleus-**NIXm** | - | - | - | - |
| Nucleus of the descending trigeminal root-**NDV** | - | - | + | + |
| Primary sensory trigeminal nucleus-**NVs** | - | - | - | - |
| Trigeminal motor nucleus, dorsal part-**NVmd** | - | + | - | - |
| Trigeminal motor nucleus, ventral part-**NVmv** | - | - | - | - |
| Trochlear nucleus-**NIV** | - | - | - | - |
| Vagal motor nucleus-**NXm** | Periventricular cells | Periventricular cells | Periventricular cells | Periventricular cells |
| Lobes | - | + | + | + |
| Facial lobe-**LVII** | Periventricular cells | + | + | + |
| Glossopharyngeal lobe-**LIX** | - | Few | + | + |
| Vagal lobe-**LX** | Periventricular cells | + | + | + |
| Octavolateral nuclei | - | + | + | + |
| Anterior octaval nucleus-**AON** | - | Scattered | - | - |
| Caudal octavolateral nucleus-**CON** | - | Few | + | + |
| Descending octaval nucleus-**DON** | - | Few | Few | Few |
| Magnocellular octaval nucleus-**MaON** | - | - | Few | Few |
| Medial octavolateral nucleus-**MON** | - | Few | - | - |
| Posterior octaval nucleus-**PON** | - | - | + | + |
| Secondary octaval population-**SO** | - | + | - | - |
|  |  |  |  |  |
| Area postrema-**AP** | - | - | - | - |
| Central gray-**GC** | + | + | + | + |
| Commissular nucleus of Cajal-**NC** | - | + | + | + |
| Inferior olive-**IO** | - | - | + | + |
| Inferior raphe-**IR** | - | Few | + | + |
| Inferior reticular formation-**IRF** | - | + | + | + |
| Intermediate reticular formation-**IMRF** | - | + | + | + |
| Lateral reticular nucleus-**LRN** | - | - | - | - |

| miRNA | *miR-92b* **Ad** | *miR-9* **Ad** | *let-7b* **Ad** | *let-7c* **Ad** |
| --- | --- | --- | --- | --- |
| Mauthner cell-**MAC** | - | - | - | - |
| Medial funicular nucleus-**MFN** | - | Few | + | + |
| Secondary gustatory nucleus-**SGN** | - | - | + | + |
| **SPINAL CORD** |  | + | + | + |
| Dorsal horn-**DH** |  | + | + | + |
| Ventral horn-**VH** |  | + | + | + |
| **COMMISSURES** | - | + | - | - |
| Postoptic commissure-**poc** |  | + |  |  |

**Table G. *miR-124, miR-219, miR-138* and *miR-135c* expression in the adult zebrafish brain**

| miRNA | *miR-124*Ad | *miR-219*Y-Ad, Ad | *miR-138*Y-Ad | *miR-135c*Ad |
| --- | --- | --- | --- | --- |
| Periventricular cells | - | - | - | + |
| Proliferative zones | - | - | - |  |
| Differentiating cells | + | + | + | + |
| Specific areas | No | Distinct cells in many different brain areas | No | No |
| Expression pattern conserved/ different between embryo and adult | Cell type conservation | Different | Mixed profile | Mixed profile |

| miRNA | *miR-124*Ad | *miR-219*Y-Ad, Ad | *miR-138*Y-Ad | *miR-135c*Ad |
| --- | --- | --- | --- | --- |
| **TELENCEPHALON-T** | **Differentiated cells throughout** | **Few distinct cells** | + | **+** |
| **Olfactory bulb- OB** | + | + | + | - |
| External cellular layer (including mitral cells)-**ECL** | + | - | - | - |
| Glomerular layer-**GL** | + | + | + | - |
| Primary olfactory fiber layer-**POF** | - | + | - | - |
| Internal cellular layer of olfactory bulb-**ICL** | + | - | + | - |
| **Ventral telencephalic area /Subpallium –V** | + | **+** | + | + |
| Central nucleus of ventral telencephalic area-**Vc** | + | - | + | - |
| Dorsal nucleus of ventral telencephalic area-**Vd** | + | + | + | Few  periventricular |
| Lateral nucleus of ventral telencephalic area-**Vl** | + | - | - | - |
| Ventral nucleus of venral telencephalic area-**Vv** | + | Lateral | + | Medial |
| Supracommissural nucleus of ventral telencephalic area-**Vs** | + | Ventral,  at the level of anterior commissure | + | + |
| Postcommissural nucleus of ventral telencephalic area-**Vp** | + | - | + | Periventricular cells |
| **Dorsal telencephalic area /Pallium -P** | + | + | + | + |
| Central zone of dorsal telencephalic area-**Dc** | + | + | - | - |
| Dorsal zone of dorsal telencephalic area-**Dd** | + | - | + | - |
| Lateral zone of dorsal telencephalic area-**Dl** | + | + | Few | - |
| Medial zone of dorsal telencephalic area-**Dm** | + | + | Few medial | - |
| Posterior zone of dorsal telencephalic area-**Dp** | + | + | + | + |
| Nucleus taeniae-**NT** | + | - | + | - |
|  |  |  |  |  |
| Endopeduncular nucleus, dorsal part **ENd** | + | - | + | - |
| Endopeduncular nucleus, ventral part **ENv** | + | - | - | + |

| miRNA | *miR-124*Ad | *miR-219*Y-Ad, Ad | *miR-138*Y-Ad | *miR-135c*Ad |
| --- | --- | --- | --- | --- |
| DIENCEPHALON | Differentiated cells throughout | Distinct cells | + | + |
| Preoptic area | + | + | + | Periventricular and adjacent cells |
| Parvocellular preoptic nucleus, anterior part-**PPa** | + | - | + | + |
| Parvocellular preoptic nucleus, posterior part-**Ppp** | + | + | - | + |
| Magnocellular preopic nucleus **PM** | + | - | - | + |
| Suprachiasmatic nucleus **SC** | + | + | - | + |
| Epithalamus | + | + | + | + |
| Pineal/epiphysis-**E** | + | Dorso-lateral | - | - |
| Dorsal Habenular nucleus-**Had** | + | + | Dorsal/lateral | Periventricular cells |
| Ventral Habenular nucleus-**Hav** | + | + | - | Periventriular cells |
| **Thalamus** | + | + | + | + |
| Anterior thalamic nucleus-**A** | + | - | - | + |
| Ventromedial thalamic nucleus-**VM** | + | - | - | + |
| Ventrolateral thalamic nucleus-**VL** | + | - | - | few |
| Central posterior thalamic nucleus-**CP** | + | Y-Ad :few | + | + |
| Dorsal posterior thalamic nucleus-**DP** | + | - | + | + |
| Intermediate thalamic nucleus-**I** | + | - | - | + |
| Hypothalamus | + | + | + | + |
| Anterior tuberal nucleus-**ATN** | + | + | - | + |
| Caudal zone of periventricular hypothalamus-**Hc** | + | - | Few ventral | + |
| Central nucleus of the inferior lobe-**CIL** | + | - | + | - |
| Diffuse nucleus of the inferior lobe-**DIL** | + | + | + | - |
| Dorsal zone of periventricular hypothalamus-**Hd** | + | - | - | + |
| Zone of lateral hypothalamic ventricular recess- **lr** | + | + | - | + |
| Lateral hypothalamic nucleus-**LH** | + | + | + | + |
| Lateral torus-**Tla** | + | - | - | Few |
| Mammilary body-**CM** | + | + | - | - |
| Ventral zone of periventricular hypothalamus-**Hv** | + | + | + | + |

| miRNA | *miR-124*Ad | *miR-219*Y-Ad, Ad | *miR-138*Y-Ad | *miR-135c*Ad |
| --- | --- | --- | --- | --- |
| Posterior tuberculum | + | + | + | + |
| Anterior preglomerular nucleus-**PGa** | + | + | + | + |
| Caudal preglomerular nucleus-**PGc** | + | - | - | - |
| Lateral preglomerular nucleus-**PGl** | + | + | - | Caudal |
| Medial preglomerular nucleus-**PGm** | + | + | + | + |
| Nucleus of Paraventricular organ-**nPVO** | + |  | - | - |
| Periventricular nucleus of posterior tuberculum-**TPp** | + | + | - | Periventricular cells |
| Posterior thalamic nucleus-**Pt** | + | + | + | - |
| Posterior tuberal nucleus-**PTN** | + | - | + | + |
| Subglomerular nucleus-**SG** | + | - | + | - |
| Tertiary gustatory nucleus-**TGN** | + | - | - | - |
| Pretectum | + | + | + | + |
| Accessory pretectal nucleus-**APN** | + | - | + | - |
| Central pretectal nucleus-**CPN** | + | + | Few | + |
| Magnocellular superficial pretectal nucleus-**PSm** | + | + | - | - |
| Parvocellular superficial pretectal nucleus-**PSp** | + | + | + | Few |
| Periventricular pretectal nucleus, dorsal part-**PPd** | + | + | - | - |
| Periventricular pretectal nucleus, ventral part-**PPv** | + | + | - | - |
| Dorsal accessory optic nucleus-**DAO** | + | + | - | - |
| Ventral accessory optic nucleus-**VAO** | + | + | - | - |
| Posterior preoptic nucleus-**PO** | + | + | - | - |
| **MIDBRAIN** | **Differentiated cells throughout** | Distinct cells | + | + |
| **Optic tectum-TeO** | + | + | Scattered cells in lateral- posterior | + |
| Superficial white zone-**swz** | + | - | - | - |
| Superficial grey zone-**sgz** | + | + | + | - |
| Central zone-**cz** | + | - | + | - |
| Deep white zone-**dwz** | + | - | + | - |
| Periventricular gray zone -**pgz(layers 1, 2, 3)** | + | + | + | 3 : dorsal |
| Proliferative zone-**m** | - | - | - | - |
| Longitudinal torus-**TL** | + | + | Few dorsal | - |
| Tegmentum | + | + | + | + |
| Central nucleus of semicircular torus-**TSc** | + | + | - | + |
| Ventrolateral nucleus of semicircular torus-**TSvl** | + | + | - | Few periventricular |
| Dorsal tegmental nucleus-**DTN** | + | - | - | - |
| Interpeduncular nucleus-**NIn** | + | - | - | - |
| Mesencephalic nucleus of trigeminal nerve-**MNV** | + | + | - | - |
| Nucleus of the medial longitudinal fascicle-**nMLF** | + | - | - | - |
| Oculomotor nucleus-**NIII** | + | - | - | - |
| Rostral tegmental nucleus-**RT** | + | Y-Ad :+ | - | - |

| miRNA | *miR-124*Ad | *miR-219*Y-Ad, Ad | *miR-138*Y-Ad | *miR-135c*Ad |
| --- | --- | --- | --- | --- |
| **HINDBRAIN** | **Differentiated cells throughout** | + | + | **+including cells around the rhombencephalic ventricle (rv)** |
| **Isthmus** | + | + | + | + |
| Isthmic nucleus-**NI** | + | - | + | + |
| Nucleus of lateral valvula-**NLV** | + | - | - | + |
| Locus coeruleus-**LC** | + | - | - | - |
| Nucleus of the lateral lemniscus-**NLL** | + | - | - | - |
| Perilemniscal nucleus-**PL** | + | + | - | + |
| Superior raphe-**SR** | + | + | - | - |
| Superior reticular formation-**SRF** | + | + | - | + |
| Cerebellum-Ce | + | + | + | - |
| Caudal lobe of cerebellum-**LCa** | + | + | - | - |
| Cerebellar corpus-**CCe** | + | Granular layer (CeGL)  Ganglionic layer  (CeGAL) | Granular layer : some (CeGL)  Ganglionic layer(CeGAL) | Granular layer (CeGL): few |
| Cerebellar crest-**CC** | + | - | - | - |
| Granular eminence-**EG** | + | + | + | Few |
| Lateral division of cerebellar valvula- **Val** | + | - | + | - |
| Medial division of cerebellar valvula -**Vam** | + | - | + | - |
| **Cranial nerve nuclei** | + | - | - | - |
| Abducens nuclei-**NVI** | + | - | - | - |
| Facial motor nucleus-**NVIIm** | + | - | - | - |
| Glossopharyngeal motor nucleus-**NIXm** | + | - | - | - |
| Nucleus of the descending trigeminal root-**NDV** | + | - | - | - |
| Primary sensory trigeminal nucleus-**NVs** | + | - | - | - |
| Trigeminal motor nucleus, dorsal part-**NVmd** | + | - | - | - |
| Trigeminal motor nucleus, ventral part-**NVmv** | + | - | - | - |
| Trochlear nucleus-**NIV** | + | - | - | - |
| Vagal motor nucleus-**NXm** | + | - | - | - |
| Lobes | + | + | + | + |
| Facial lobe-**LVII** | + | + | + | + |
| Glossopharyngeal lobe-**LIX** | + | - | - | + |
| Vagal lobe-**LX** | + | + | + | + |
| Octavolateral nuclei | + | + | + | + |
| Anterior octaval nucleus-**AON** | + | + | - | - |
| Caudal octavolateral nucleus-**CON** | + | + | - | Few |
| Descending octaval nucleus-**DON** | + | + | - | Few ventral |
| Magnocellular octaval nucleus-**MaON** | + | - | - | - |
| Medial octavolateral nucleus-**MON** | + | + | + | Few dorsal |
| Posterior octaval nucleus-**PON** | + | + | - | - |
| Secondary octaval population-**SO** | + | + | - | - |
|  |  |  |  |  |
| Area postrema-**AP** | + | + | - | - |
| Central gray-**GC** | - | - | - | + |
| Commissular nucleus of Cajal-**NC** | + | + | few | - |
| Inferior olive-**IO** | + | + | + | - |
| Inferior raphe-**IR** | + | + | - | Few |
| Inferior reticular formation-**IRF** | + | Scattered cells | Few | + |
| Intermediate reticular formation-**IMRF** | + | Scattered cells | - | - |
| Lateral reticular nucleus-**LRN** | + | + | - | - |
| Mauthner cell-**MAC** | + | - | - | - |
| Medial funicular nucleus-**MFN** | + | + | - | - |
| Secondary gustatory nucleus-**SGN** | + | - | - | - |

| miRNA | *miR-124*Ad | *miR-219*Y-Ad, Ad | *miR-138*Y-Ad | *miR-135c*Ad |
| --- | --- | --- | --- | --- |
| **SPINAL CORD** | Differentiated cells throughout | + |  |  |
| Dorsal horn-**DH** | + | + |  |  |
| Ventral horn-**VH** | + | + |  |  |
| **COMMISSURES** |  | Cells related with |  |  |
| Anterior commisure-**ac** |  | + |  |  |
| Cerebellar commissure-**Ccer** |  | - |  |  |
| Commissure infima of Haller-**Cinf** |  | + |  |  |
| Commissure of the posterior tuberculum-**Ctub** |  | - |  |  |
| Commissure of the secondary gustatory tract-**Cgus** |  | - |  |  |
| Habenular commissure-**Chab** |  | + |  |  |
| Horizontal commissure-**Chor** |  | - |  |  |
| Optic chiasma-**OC** |  | + |  |  |
| Posterior commissure-**Cpost** |  | + |  |  |
| Postoptic commissure-**poc** |  | + |  |  |
| Tectal commissure-**Ctec** |  | + |  |  |
| Ventral rhomboncephalic commissure-**Cven** |  | - |  |  |

| miRNA | *miR-124*Ad | *miR-219*Y-Ad, Ad | *miR-138*Y-Ad | *miR-135c*Ad |
| --- | --- | --- | --- | --- |
| **NERVES** |  | Cells associated with |  |  |
| Anterior lateral line nerves-**A LLN** |  | + |  |  |
| Descending trigeminal root-**DV** |  | + |  |  |
| Oculomotor nerve-**III** |  | + |  |  |
| Olfactory nerve-**I** |  | + |  |  |
| Optic nerve- **II** |  | + |  |  |
| Posterior lateral line nerve-**PLLN** |  | + |  |  |
| Trigeminal nerve-**V** |  | + |  |  |
| Trochlear nerve-**IV** |  | + |  |  |
| Vagal nerve-**X** |  | + |  |  |
| **TRACTS** |  | + |  |  |
| Bulbo-spinal tract-**TBS** |  | + |  |  |
| Lateral forebrain bundle-**LFB** |  | +at preglomerular complex level |  |  |
| Lateral lemniscus=lateral longitudinal fascicle-**LLF** |  | +at superior reticular formation level |  |  |
| Lateral olfactory tract-**LOT** |  | + |  |  |
| Medial longitudinal tract-**MLF** |  | + |  |  |
| Medial olfactory tract-**MOT** |  | + |  |  |
| Optic tract-**OT** ventrolateral **VOT** and dorsomedial **DOT** |  | + |  |  |
| Tectobulbar tract-**TTB** |  | + |  |  |

**Table H. *miR-128, miR-137, miR-181b, miR-181a* and *miR-153a* expression in the adult zebrafish brain.**

| miRNA | ***miR-128***  **Ad** | *miR-137*Y-Ad, Ad | *miR-181b*Ad | *miR-181a*Ad | *miR-153a*Y-Ad, Ad |
| --- | --- | --- | --- | --- | --- |
| Periventricular cells | - | - | - | - | Few cases |
| Proliferative zones | - | - | - | - | - |
| Differentiating cells | + | + | + | + | + |
| Specific brain areas | yes | yes | yes | yes | + |
| Expression pattern conserved/different between embryo and adult | Conserved | Conserved | Mixed profile | Mixed profile | Mixed profile |

| miRNA | ***miR-128***  **Ad** | *miR-137*Y-Ad, Ad | *miR-181b*Ad | *miR-181a*Ad | *miR-153a*Y-Ad, Ad |
| --- | --- | --- | --- | --- | --- |
| **TELENCEPHALON-Te** | + | + | + | + | + |
| **Olfactory bulb- OB** | + | + | + | + | + |
| External cellular layer (including mitral cells)-**ECL** | + | - | + | + | + |
| Glomerular layer-**GL** | + | - | Few | Few | + |
| Primary olfactory fiber layer-**POF** | + | Few | - | - | - |
| Internal cellular layer of olfactory bulb-**ICL** | + | - | + | + | + |
| **Ventral telencephalic area /Subpallium –V** | + | + | Weak | Weak | + |
| Central nucleus of ventral telencephalic area-**Vc** | + | + | Ventral | Ventral | + |
| Dorsal nucleus of ventral telencephalic area-**Vd** | + | + | Few | Few | Few :weak, rostral |
| Lateral nucleus of ventral telencephalic area-**Vl** | Weak | Weak | - | - | Weak |
| Ventral nucleus of venral telencephalic area-**Vv** | + | + | + | + | + |
| Supracommissural nucleus of ventral telencephalic area-**Vs** | Weak | + | + | + | Few scattered cells |
| Postcommissural nucleus of ventral telencephalic area-**Vp** | Weak | + | + | + | Few scattered cells |
| **Dorsal telencephalic area /Pallium -P** | + | + | Weak | Weak | + |
| Central zone of dorsal telencephalic area-**Dc** | + | - | Some | Few | + |
| Dorsal zone of dorsal telencephalic area-**Dd** | + | Ventral row of cells | + | + | + |
| Lateral zone of dorsal telencephalic area-**Dl** | + | Caudal | + | + | + including periventricular cells.  Caudo-dorsal :weak |
| Medial zone of dorsal telencephalic area-**Dm** | + | Rostral : medial  Caudal : dorsal and medial | + | + | Rostral :+ incuding dorsal periventricular cells.  Caudal : +  Medial :weak scattered.  Lateral : rows of cells towards Vc |
| Posterior zone of dorsal telencephalic area-**Dp** | + | +  including medial cells | + | Few | + |
| Nucleus taeniae-**NT** | + | - | - | - | - |
|  |  |  |  |  |  |
| Endopeduncular nucleus, dorsal part **ENd** | + | + | + | + | + |
| Endopeduncular nucleus, ventral part **ENv** | + | - | - | - | + |

| miRNA | ***miR-128***  **Ad** | *miR-137*Y-Ad, Ad | *miR-181b*Ad | *miR-181a*Ad | *miR-153a*Y-Ad, Ad |
| --- | --- | --- | --- | --- | --- |
| DIENCEPHALON | + | + | + | + | + |
| Preoptic area | + | + | Weak | Weak | + |
| Parvocellular preoptic nucleus, anterior part-**PPa** | Weak | + | Few | Few | + |
| Parvocellular preoptic nucleus, posterior part-**Ppp** | Weak | Weak | Few | Few | + |
| Magnocellular preopic nucleus **PM** | - | + | - | - | + |
| Suprachiasmatic nucleus **SC** | - | Weak | Few | Few | + |
| Epithalamus | - | + | + | + | + |
| Pineal/epiphysis-**E** | - | - | - | - | - |
| Dorsal Habenular nucleus-**Had** | + | Groups of cells | Few weak | Few weak | + |
| Ventral Habenular nucleus-**Hav** | some cells | - | - | - | + |
| **Thalamus** | + | + | - | - | + |
| Anterior thalamic nucleus-**A** | - | - | - | - | - |
| Ventromedial thalamic nucleus-**VM** | - | + | - | - | - |
| Ventrolateral thalamic nucleus-**VL** | + | - | - | - | + |
| Central posterior thalamic nucleus-**CP** | - | + | - | - | + |
| Dorsal posterior thalamic nucleus-**DP** | - | Some | - | - | - |
| Intermediate thalamic nucleus-**I** | - | - | - | - | - |
| Hypothalamus | + | + | - | - | + |
| Anterior tuberal nucleus-**ATN** | + | + | + | Few | Weak |
| Caudal zone of periventricular hypothalamus-**Hc** | + | + | + | + | + |
| Central nucleus of the inferior lobe-**CIL** | + | - | Few | Few | Weak |
| Diffuse nucleus of the inferior lobe-**DIL** | + | + | Few | Few | + |
| Dorsal zone of periventricular hypothalamus-**Hd** | + | + | - | - | + |
| Dorsal zone of periventricular hypothalamus- Lateral recess –**Hd-lr** | + | Rostral | Few | Caudal | + |
| Lateral hypothalamic nucleus-**LH** | + | + | Few | Few | + |
| Lateral torus-**TLa** | + | + | - | - | + |
| Mammilary body-**CM** | + | - | Few | + | - |
| Ventral zone of periventricular hypothalamus-**Hv** | + | + | Few | Few | + |

| miRNA | ***miR-128***  **Ad** | *miR-137*Y-Ad, Ad | *miR-181b*Ad | *miR-181a*Ad | *miR-153a*Y-Ad, Ad |
| --- | --- | --- | --- | --- | --- |
| Posterior tuberculum | + | + | - | - | + |
| Anterior preglomerular nucleus-**PGa** | + | + | - | - | + |
| Caudal preglomerular nucleus-**PGc** | - | - | - | - | - |
| Lateral preglomerular nucleus-**PGl** | + | Few | - | + | + |
| Medial preglomerular nucleus-**PGm** | + | Weak | - | - | Rostral |
| Nucleus of Paraventricular organ-**nPVO** | + | Few | - | - | + |
| Periventricular nucleus of posterior tuberculum-**TPp** | Weak | Ventral | - | - | rostral |
| Posterior thalamic nucleus-**Pt** | + | + | - | - | + |
| Posterior tuberal nucleus-**PTN** | + | + | - | - | + |
| Subglomerular nucleus-**SG** | + | + | - | - | - |
| Tertiary gustatory nucleus-**TGN** | + | - | - | - | - |
| Pretectum | + | - | + | + | - |
| Accessory pretectal nucleus-**APN** | - | - | + | + | - |
| Central pretectal nucleus-**CPN** | + | - | + | + | - |
| Magnocellular superficial pretectal nucleus-**PSm** | + | - | + | + | - |
| Parvocellular superficial pretectal nucleus-**PSp** | + | - | + | + | - |
| Periventricular pretectal nucleus, dorsal part-**PPd** | - | - | - | - | - |
| Periventricular pretectal nucleus, ventral part-**PPv** | - | - | - | - | - |
| Dorsal accessory optic nucleus-**DAO** | - | - | + | + | - |
| Ventral accessory optic nucleus-**VAO** | - | - | - | - | - |
| Posterior preoptic nucleus-**PO** | Weak | - | Few weak | Few weak | - |
| **MIDBRAIN** | + | + | + | + | + |
| **Optic tectum-TeO** | + | + | Scattered cells throughout all layers | Scattered cells throughout all layers | + |
| Superficial white zone-**swz** | - | - | + | + | - |
| Superficial grey zone-**sgz** | + | - | + | + | - |
| Central zone-**cz** | Few | - | + | + | - |
| Deep white zone-**dwz** | - | - | - | - | - |
| Periventricular gray zone of optic tectum-**pgz (layers 1,2,3)** | 3 | Some weak | + | + | Y-Ad :3  A : less cells, caudal |
| Proliferative zone-**m** | - | - | - | - | - |
| Longitudinal torus-**TL** | Dorsal | - | Few | - | - |
| Tegmentum | + | + | - | - | + |
| Central nucleus of semicircular torus-**TSc** | + | Y-Ad :+ | - | - | Y-Ad:+ |
| Ventrolateral nucleus of semicircular torus-**TSvl** | Rostral | Y-Ad :+ | - | - | Y-Ad :+ |
| Dorsal tegmental nucleus-**DTN** | - | + | - | - | - |
| Mesencephalic nucleus of trigeminal nerve-**MNV** | - | - | - | - | - |
| Nucleus of the medial longitudinal fascicle-**nMLF** | - | - | - | - | - |
| Oculomotor nucleus-**NIII** | - | - | - | - | - |
| Rostral tegmental nucleus-**RT** | - | - | - | - | - |
| Interpeduncular nucleus-**NIn** | - | - | Few | Few | Y-Ad :+ |

| miRNA | ***miR-128***  **Ad** | *miR-137*Y-Ad, Ad | *miR-181b*Ad | *miR-181a*Ad | *miR-153a*Y-Ad, Ad |
| --- | --- | --- | --- | --- | --- |
| Mauthner cell-**MAC** | - | - | - | - | - |
| Medial funicular nucleus-**MFN** | + | - | - | - | - |
| Secondary gustatory nucleus-**SGN** | + | - | - | - | - |
| **SPINAL CORD** | + | + | - | - | - |
| Dorsal horn-**DH** | + | + | - | - | - |
| Ventral horn-**VH** | - | - | - | - | - |
| **COMMISSURES** | - | - | - | - | - |

| miRNA | ***miR-128***  **Ad** | *miR-137*Y-Ad, Ad | *miR-181b*Ad | *miR-181a*Ad | *miR-153a*Y-Ad, Ad |
| --- | --- | --- | --- | --- | --- |
| **TRACTS** | - | + |  |  |  |
| Lateral olfactory tract-**LOT** |  | Cells at level of posterior dorsal telencephalon (Dp) |  |  |  |
| Medial olfactory tract-**MOT** |  | Cells at level of the olfactory bulb (OB) and posterior dorsal telencephalon(Dp) |  |  |  |

**Table I. *miR-218a, miR-34, miR-222, miR-221, miR-7* and *miR-200a* expression in the adult zebrafish brain.**

| miRNA | ***miR-218a*** Y-Ad, Ad | ***miR-34*** Ad | ***miR-222*** Y-Ad, Ad | *miR-221* **Ad** | ***miR-7*** Ad | ***miR-200a***  **Ad** |
| --- | --- | --- | --- | --- | --- | --- |
| Periventricular cells | - | - | - | - | - | - |
| Proliferative zones | - | - | - | - | - | - |
| Differentiating cells | + | + | + | + | + | + |
| Specific areas | Yes | Yes | Yes | yes | Forebrain, area postrema | Primary olfactory fiber layer |
| Exprresion pattern conserved between embryo and adult | No | No | Mixed profile | Mixed profile | Mainly yes |  |

| miRNA | ***miR-218a*** Y-Ad, Ad | ***miR-34*** Ad | ***miR-222*** Y-Ad, Ad | *miR-221*Ad | ***miR-7*** Ad | ***miR-200a***  **Ad** |
| --- | --- | --- | --- | --- | --- | --- |
| **TELENCEPHALON-Te** | + | - | + | + | + | + |
| **Olfactory bulb- OB** | - | - | + | + | + | + |
| External cellular layer (including mitral cells)-**ECL** | - | - | - | - | + | - |
| Glomerular layer-**GL** | - | - | - | - | - | - |
| Primary olfactory fiber layer-**POF** | - | - | - | - | - | + |
| Internal cellular layer of olfactory bulb-**ICL** | - | - | Weak | Weak | + | - |
| **Ventral telencephalic area /Subpallium -V** | - | - | + | + | + | - |
| Central nucleus of ventral telencephalic area-**Vc** | - | - | + | + | + | - |
| --Dorsal nucleus of ventral telencephalic area-**Vd** | - | - | Weak | + | + | - |
| Lateral nucleus of ventral telencephalic area-**Vl** | - | - | - | - | - | - |
| Ventral nucleus of venral telencephalic area-**Vv** | + | - | + | + | + | - |
| Supracommissural nucleus of ventral telencephalic area-**Vs** | - | - | Weak | + | Weak | - |
| Postcommissural nucleus of ventral telencephalic area-**Vp** | - | - | - | + | Weak | - |
| **Dorsal telencephalic area /Pallium -P** | - | - | + | + | + | - |
| Central zone of dorsal telencephalic area-**Dc** | - | - | + | + | Rostral | - |
| Dorsal zone of dorsal telencephalic area-**Dd** | - | - | + | + | + | - |
| Lateral zone of dorsal telencephalic area-**Dl** | - | - | + | + | +  (caudal :weak) | - |
| Medial zone of dorsal telencephalic area-**Dm** | - | - | + | + | + | - |
| Posterior zone of dorsal telencephalic area-**Dp** | - | - | Few | + | Weak | - |
| Nucleus taeniae-**NT** | - | - | - | - | - | - |
|  |  |  |  |  |  | - |
| Endopeduncular nucleus, dorsal part **ENd** | - | - | + | - | + | - |
| Endopeduncular nucleus, ventral part **ENv** | - | - | - | - | - | - |

| miRNA | ***miR-218a*** Y-Ad, Ad | ***miR-34*** Ad | ***miR-222*** Y-Ad, Ad | *miR-221*Ad | ***miR-7*** Ad | ***miR-200a***  **Ad** |
| --- | --- | --- | --- | --- | --- | --- |
| DIENCEPHALON | + | + | + | + | + | - |
| Preoptic area | + | - | + | + | + | - |
| Parvocellular preoptic nucleus, anterior part-**PPa** | few | - | + | + | + | - |
| Parvocellular preoptic nucleus, posterior part-**PPp** | few | - | - | + | + | - |
| Magnocellular preopic nucleus-**PM** | + | - | - | Few | + | - |
| Suprachiasmatic nucleus-**SC** | - | - | - | + | + | - |
| Epithalamus | - | + | - | - | - | - |
| Pineal/epiphysis-**E** | - | Few weak | - | - | - | - |
| Dorsal Habenular nucleus-**Had** | - | Group of cells | - | - | - | - |
| Ventral Habenular nucleus-**Hav** | - | - | - | - | - | - |
| **Thalamus** | - | + | - | + | - | - |
| Anterior thalamic nucleus-**A** | - | - | - | - | - | - |
| Ventromedial thalamic nucleus-**VM** | - | - | - | Few | - | - |
| Ventrolateral thalamic nucleus-**VL** | - | Few | - | + | - | - |
| Central posterior thalamic nucleus-**CP** | - | - | - | Weak | - | - |
| Dorsal posterior thalamic nucleus-**DP** | - | - | - | Weak | - | - |
| Intermediate thalamic nucleus-**I** | - | - | - | - | - | - |
| Hypothalamus | + | - | + | + | + | - |
| Anterior tuberal nucleus-**ATN** | - | Few | Some | + | - | - |
| Caudal zone of periventricular hypothalamus-**Hc** | - | - | Some | Few | Ventral | - |
| Central nucleus of the inferior lobe-**CIL** | - | - | + | + | - | - |
| Diffuse nucleus of the inferior lobe-**DIL** | - | - | + | + | Rostral | - |
| Dorsal zone of periventricular hypothalamus-**Hd** | - | Caudal | Some | + | - | - |
| Zone of hypothalamic ventricular lateral recess –**Hd-lr** | - | - | Some | Few | Rostral | - |
| Lateral hypothalamic nucleus-**LH** | + | - | + | + | + | - |
| Lateral torus-**TLa** | - | - | + | - | Few | - |
| Mammilary body-**CM** | - | - | + | + | - | - |
| Ventral zone of periventricular hypothalamus-**Hv** | Ventral rostral | - | + | + | + | - |

| miRNA | ***miR-218a*** Y-Ad, Ad | ***miR-34*** Ad | ***miR-222*** Y-Ad, Ad | *miR-221*Ad | ***miR-7*** Ad | ***miR-200a***  **Ad** |
| --- | --- | --- | --- | --- | --- | --- |
| Posterior tuberculum | - | + | + | + | - | - |
| Anterior preglomerular nucleus-**PGa** | - | - | - | + | - | - |
| Caudal preglomerular nucleus-**PGc** | - | - | - | - | - | - |
| Lateral preglomerular nucleus-**PGl** | - | - | - | + | - | - |
| Medial preglomerular nucleus-**PGm** | - | Few | - | + | - | - |
| Nucleus of paraventricular organ-**nPVO** | - | + | + | + | - | - |
| Periventricular nucleus of posterior tuberculum-**TPp** | - | + including group of periventri-cular cells | Few | + | - | - |
| Posterior thalamic nucleus-**Pt** | - | - | + | + | - | - |
| Posterior tuberal nucleus-**PTN** | - | - | - | Few | - | - |
| Subglomerular nucleus- **SG** | - | - | - | Weak | - | - |
| Tertiary gustatory nucleus-**TGN** | - | Few | - | Weak | - | - |
| Pretectum | - | + | - | + | - | - |
| Accessory pretectal nucleus-**APN** | - | Some | - | - | - | - |
| Central pretectal nucleus-**CPN** | - | - | - | - | - | - |
| Magnocellular superficial pretectal nucleus-**PSm** | - | Few | - | + | - | - |
| Parvocellular superficial pretectal nucleus-**PSp** | - | - | - | - | - | - |
| Periventricular pretectal nucleus, dorsal part-**PPd** | - | - | - | - | - | - |
| Periventricular pretectal nucleus, ventral part-**PPv** | - | - | - | - | - | - |
| Dorsal accessory optic nucleus-**DAO** | - | + | - | + | - | - |
| Ventral accessory optic nucleus-**VAO** | - | + | - | Few | - | - |
| Posterior pretectal nucleus-**PO** | - | - | - | - | - | - |
| **MIDBRAIN** | - | + | + | + | - | - |
| **Optic tectum-TeO** | + | + | + | + | - | - |
| Superficial white zone-**swz** | - | - | - | - | - | - |
| Superficial grey zone-**sgz** | - | + | - | - | - | - |
| Central zone-**cz** | - | + | - | - | - | - |
| Deep white zone-**dwz** | - | - | - | + | - | - |
| Periventricular gray zone -**pgz (layers 1, 2, 3)** | 3 | 3 | 3 :some | Rows of cells | - | - |
| Proliferative zone-**m** | - | - | - | - | - | - |
| Longitudinal torus-**TL** | - | - | - | - | - | - |
| Tegmentum | + | + | - | + | - | - |
| Central nucleus of semicircular torus-**TSc** | - | Few | - | + | - | - |
| Ventrolateral nucleus of semicircular torus-**TSvl** | - | Few | - | + | - | - |
| Dorsal tegmental nucleus-**DTN** | - | - | - | - | - | - |
| Interpeduncular nucleus-**NIn** | - | - | - | - | - | - |
| Mesencephalic nucleus of trigeminal nerve-**MNV** | - | Weak | - | - | - | - |
| Nucleus of the medial longitudinal fascicle-**nMLF** | - | + | - | - | - | - |
| Oculomotor nucleus-**NIII** | + | Weak | - | - | - | - |
| Rostral tegmental nucleus-**RT** | - | Weak | - | - | - | - |

| miRNA | ***miR-218a*** Y-Ad, Ad | ***miR-34*** Ad | ***miR-222*** Y-Ad, Ad | ***miR-221*** Ad | ***miR-7*** Ad | ***miR-200a***  **Ad** |
| --- | --- | --- | --- | --- | --- | --- |
| **HINDBRAIN** | + | + | + | + | + | - |
| Isthmus | - | + | - | + | - | - |
| Isthmic nucleus-**NI** | - | + | - | + | - | - |
| Nucleus of lateral valvula-**NLV** | - | Weak group | - | Weak | - | - |
| Locus coeruleus-**LC** | - | + | - | - | - | - |
| Nucleus of the lateral lemniscus-**NLL** | - | - | - | - | - | - |
| Perilemniscal nucleus-**PL** | - | Weak | - | - | - | - |
| Superior raphe-**SR** | - | - | - | - | - | - |
| Superior reticular formation-**SRF** | - | + | - | - | - | - |
| Cerebellum-Ce | - | + | - | - | - | - |
| Caudal lobe of cerebellum-**Lca** | - | - | - | - | - | - |
| Cerebellar corpus-**CCe** | - | Granular layer (CeGL) | - | - | - | - |
| Cerebellar crest-**CC** | - | - | - | - | - | - |
| Granular eminence-**EG** | - | Weak | - | - | - | - |
| Lateral division of cerebellar valvula -**Val** | - | - | - | - |  | - |
| Medial division of cerebellar valvula-**Vam** | - | - | - | - | - | - |
| **Cranial nerve nuclei** | + | + | - | - | - | - |
| Abducens nuclei -**NVI** | Rostral and caudal | Caudal | - | - | - | - |
| Glossopharyngeal motor nucleus-**NIXm** | + | - | - | - | - | - |
| Facial motor nucleus-**NVIIm** | + | - | - | - | - | - |
| Nucleus of the descending trigeminal root-**NDV** | - | - | - | - | - | - |
| Primary sensory trigeminal nucleus-**NVs** | - | + | - | - | - | - |
| Trigeminal motor nucleus, dorsal part-**NVmd** |  | + | - | - | - | - |
| Trigeminal motor nucleus, ventral part-**NVmv** | + | + | - | - | - | - |
| Trochlear nucleus-**NIV** | - | Weak | - | - | - | - |
| Vagal motor nucleus-**NXm** | + | - | - | - | - | - |
| Octavolateral nuclei | - | + | - | - | - | - |
| Anterior octaval nucleus-**AON** | - | + | - | - | - | - |
| Caudal octavolateral nucleus-**CON** | - | + | - | - | - | - |
| Descending octaval nucleus-**DON** | - | + | - | - | - | - |
| Magnocellular octaval nucleus-**MaON** | - | + | - | - | - | - |
| Medial octavolateral nucleus-**MON** | - | + | - | - | - | - |
| Posterior octaval nucleus-**PON** | - | + | - | - | - | - |
| Secondary octaval population-**SO** | - | Few | - | - | - | - |
| Lobes | - | + | + | + | - | - |
| Facial lobe-**LVII** | - | Caudal | A :Dorsal  Y-Ad : 3-4 weak cells | + | - | - |
| Glossopharyngeal lobe-**LIX** | - | Weak | - | - | - | - |
| Vagal lobe-**LX** | - | + | A :+  Y-Ad :- | Dorsal/lateral | - | - |
|  |  |  |  |  |  | - |
| Area postrema-**AP** | - | - | - | - | + | - |
| Central gray-**GC** | - | - | - | - | - | - |
| Commissular nucleus of Cajal-**NC** | Weak | - | - | - | - | - |
| Inferior olive-**IO** | + | - | - | - | - | - |
| Inferior raphe-**IR** | - | - | - | - | - | - |
| Inferior reticular formation-**IRF** | - | + | - | - | - | - |
| Intermediate reticular formation-**IMRF** | - | + | - | - | - | - |
| Lateral reticular nucleus-**LRN** | - | - | - | - | - | - |

| miRNA | ***miR-218a*** Y-Ad, Ad | ***miR-34*** Ad | ***miR-222*** Y-Ad, Ad | *miR-221*Ad | ***miR-7*** Ad | ***miR-200a***  **Ad** |
| --- | --- | --- | --- | --- | --- | --- |
| Mauthner cell-**MAC** | - | + | - | - | - | - |
| Medial funicular nucleus-**MFN** | - | + | - | - | - | - |
| Secondary gustatory nucleus-**SGN** | - | Lateral | - | Few lateral | - | - |
| **SPINAL CORD** | + | + | - | - | - | - |
| Dorsal horn-**DH** | - | + | - | - | - | - |
| -Ventral horn-**VH** | +(moto- -neurons) | + | - | - | - | - |
| **COMMISSURES** | - | - | - | - | - | - |

| miRNA | ***miR-218a*** Y-Ad, Ad | ***miR-34*** Ad | ***miR-222*** Y-Ad, Ad | *miR-221*Ad | ***miR-7*** Ad | ***miR-200a***  **Ad** |
| --- | --- | --- | --- | --- | --- | --- |
| **NERVES** |  |  |  |  | Cells associated with | - |
| Facial nerve-**VII** |  |  |  |  | + |  |
| Octaval nerve-**VIII** |  |  |  |  | + |  |
