# Supplementary material for: MicroRNAs show a wide diversity of expression profiles in the developing and mature central nervous system
Source: Genome Biol. 2007 Aug 21;8(8):R173. doi: 10.1186/gb-2007-8-8-r173 (PMC2375003; doi:10.1186/gb-2007-8-8-r173)
Supplement: Additional data file 28 — Tables J-L, listing the miRNAs analyzed, the family/cluster they belong to and the mismatch locked nucleic acid (LNA) probe sequences used to test specificity of corresponding fully matching LNA probes. [file gb-2007-8-8-r173-S28.doc]

Table J. miRNA probe sequences and their hybridisation temperatures.

| miRNA name | probe sequence | Hybridisation Temperature | Exiqon ID |
| --- | --- | --- | --- |
| miR7 | AACAAAATCACTAGTCTTCCA | 42 | 17224 |
| miR9 | TCATACAGCTAGATAACCAAAGA | 53 | 17067 |
| miR16 | CCAATATTTACGTGCTGCTA | 47 | 17338 |
| miR34 | AACAACCAGCTAAGACACTGCCA | 59 | 17223 |
| miR34b | CAATCAGCTAACAACACTGCCTA | 53 | 17321 |
| miR92b | GGAGGCCGGGACGAGTGCAATAT | 60 | 17801 |
| miR96 | AGCAAAAATGTGCTAGTGCCAAA | 53 | 17275 |
| miR98 | AACAACACAACTTACTACCTCA | 47 | 17330 |
| miR99 | CACAAGATCGGATCTACGGGTT | 53 | 17276 |
| miR100 | CACAAGTTCGGATCTACGGGTT | 53 | 17222 |
| miR103 | TCATAGCCCTGTACAATGCTGCT | 59 | 17249 |
| miR124 | TGGCATTCACCGCGTGCCTTAA | 59 | 17290 |
| miR125a | CACAGGTTAAGGGTCTCAGGGA | 59 | 17324 |
| miR125b | TCACAAGTTAGGGTCTCAGGGA | 53 | 17225 |
| miR128 | AAAAGAGACCGGTTCACTGTGA | 53 | 17245 |
| miR132 | CGACCATGGCTGTAGACTGTTA | 53 | 17253 |
| miR135c | CACATAGGAATAGAAAGCCATA | 47 | 19205 |
| miR137 | CTACGCGTATTCTTAAGCAATA | 47 | 17255 |
| miR138 | GATTCACAACACCAGCT | 50 | 17538 |
| miR139 | AGACACATGCACTGTAGA | 47 | 17327 |
| miR153a | TCACTTTTGTGACTATGCAA | 47 | 17230 |
| miR181a | ACTCACCGACAGCGTTGAATGTT | 59 | 17063 |
| miR181b | AACCCACCGACAGCAATGAATGTT | 59 | 17259 |
| miR182 | TGTGAGTTCTACCATTGCCAAA | 47 | 17342 |
| miR183 | CAGTGAATTCTACCAGTGCCATA | 53 | 17260 |
| miR187 | GTTCCACTGGCTGCAACACAAG | 59 | 19206 |
| miR200a | ACATCGTTACCAGACAGTGTTA | 47 | 17256 |
| miR200b | CATCATTACCAGGCAGTATTAGA | 47 | 17326 |
| miR218a | ACATGGTTAGATCAAGCACAA | 47 | 17216 |
| miR219 | AGAATTGCGTTTGGACAATCA | 47 | 17238 |
| miR221 | GAAACCCAGCAGACAATGTAGCT | 59 | 17267 |
| miR222 | GAGACCCAGTAGCCAGATGTAGCT | 59 | 17217 |
| mir375 | TAACGCGAGCCGAACGAACAAA | 60 | 17543 |
| miR429 | ACGGCATTACCAGACAGTATTA | 51 | 17832 |
| miR454a | CCCTATTAGCAATATTGCACTA | 45 | 19211 |
| let7a | AACTATACAACCTACTACCTCA | 53 | 17066 |
| let7b | AACCACACAACCTACTACCTCA | 53 | 17248 |
| let7c | AACCATACAACCTACTACCTCA | 53 | 17247 |

Table K. *Danio rerio* miRNAs belonging to the same family or cluster. Differences between family members are highlighted in red.

| **miRNA families** | |
| --- | --- |
| **miRNA name** | **miRNA sequence** |
| dre-miR-153a | UUGCAUAGUCACAAAAGUGAUC |
| dre-miR-153b | UUGCAUAGUCACAAAAAUGAGC |
| dre-miR-153c | UUGCAUAGUCACAAAAAUGAUC |
|  |  |
| dre-miR-181a | AACAUUCAACGCUGUCGGUGAGU |
| dre-miR-181b | AACAUUCAUUGCUGUCGGUGGG |
| dre-miR-181c | CACAUUCAUUGCUGUCGGUGGG |
|  |  |
| dre-miR-34 | UGGCAGUGU CUUAGCUGGUUGU |
| dre-miR-34b | UAGGCAGUGUUGUUAGCUGAUUG |
| dre-miR-34c | AGGCAGUGCAGUUAGUUGAUUAC |
|  |  |
| dre-miR-135a | UAUGGCUUUUUAUUCCUAUGUGA |
| dre-miR-135b | UAUGGCUUUUUAUUCCUAUCUG |
| dre-miR-135c | UAUGGCUUUCUAUUCCUAUGUG |
|  |  |
| dre-miR-92a | UAUUGCACUUGUCCCGGCCUGU |
| dre-miR-92b | UAUUGCACUCGUCCCGGCCUCC |
|  |  |
| dre-let-7a | UGAGGUAGUAGGUUGUAUAGUU |
| dre-let-7b | UGAGGUAGUAGGUUGUGUGGUU |
| dre-let-7c | UGAGGUAGUAGGUUGUAUGGUU |
| dre-let-7d | UGAGGUAGUUGGUUGUAUGGUU |
| dre-let-7e | UGAGGUAGUAGAUUGAAUAGUU |
| dre-let-7f | UGAGGUAGUAGAUUGUAUAGUU |
| dre-let-7g | UGAGGUAGUAGUUUGUAUAGUU |
| dre-let-7h | UGAGGUAGUAAGUUGUGUUGUU |
| dre-let-7i | UGAGGUAGUAGUUUGUGCUGUU |
| dre-let-7j | UGAGGUAGUUGUUUGUACAGUU |
|  |  |
| dre-miR-125a | UCCCUGAGACCCUUAACCUGUG |
| dre-miR-125b | UCCCUGAGACCCU AACUUGUGA |
| dre-miR-125c | UCCCUGAGACCCU AACUCGUGA |
|  |  |
| dre-miR-218a | UUGUGCUUGAUCUAACCAUGUG |
| dre-miR-218b | UUGUGCUUGAUCUAACCAUGCA |
|  |  |
| dre-mir-454a | UAGUGCAAUAUUGCUAAUAGGG |
| dre-mir-454b | UAGUGCAAUAUUGCUUAUAGGG |
|  |  |
| **miRNA clusters** | |
| **miRNA name** | **miRNA sequence** |
| dre-miR-96 | UUUGGCACUAGCACAUUUUUGCU |
| dre-miR-182 | UUUGGCAAUGGUAGAACUCACA |
| dre-miR-183 | UAUGGCACUGGUAGAAUUCACUG |
|  |  |
| dre-miR-221 | AGCUACAUU GUCUGCUGGGUUUC |
| dre-miR-222 | AGCUACAUCUGGCUACUGGGUCUC |
|  |  |
| dre-miR-99 | AACCCGUAGAUCCGAUCUUGUG |
| dre-let-7c | UGAGGUAGUAGGUUGUAUGGUU |
| dre-miR-125b | UCCCUGAGACCCUAACUUGUGA |
|  |  |
| dre-let-7a | UGAGGUAGUAGGUUGUAUAGUU |
| dre-let-7b | UGAGGUAGUAGGUUGUGUGGUU |
|  |  |
| dre-miR-100 | AACCCGUAGAUCCGAACUUGUG |
| dre-miR-125b | UCCCUGAGACCCUAACUUGUGA |
| dre-let-7a | UGAGGUAGUAGGUUGUAUAGUU |
|  |  |
| dre-miR-200a | UAACACUGUCUGGUAACGAUGU |
| dre-miR-200b | UAAUACUGCCUGGUAAUGAUGA |
| dre-miR-200c | UAAUACUGCCUGGUAAUGAUGC |
| dre-miR-429 | UAAUACUGUCUGGUAAUGCCGU |

Table L. Mismatch LNA probe sequences used to test the specificity of corresponding fully matching LNA probes.

| based on miRNA | based on Exiqon ID | probe name | mismatch probe | Exiqon ID |
| --- | --- | --- | --- | --- |
| dre-miR-92b | 17801 | MMmiR-92b | ggaggcccggacgagtgcaatat | 32275 |
| dre-miR-153a | 17230 | MMmiR-153a | tcagttttgtgactatgcaa | 32273 |
| dre-miR-181a | 17063 | MMmiR-181a | actcaccgacagccctgaatgtt | 32274 |
| dre-let-7a | 17066 | MMlet7a | aactatacaacctagtacctca | 32276 |
